# On the Reliability of the EEG Microstate Approach

**DOI:** 10.1007/s10548-023-00982-9

**Published:** 2023-07-06

**Authors:** Tobias Kleinert, Thomas Koenig, Kyle Nash, Edmund Wascher

**Affiliations:** 1 Department of Ergonomics, Leibniz Research Centre for Working Environment and Human Factors, Ardeystr. 67, 44139 Dortmund, Germany; 2 Department of Biological Psychology, Clinical Psychology, and Psychotherapy, University of Freiburg, Stefan-Meier Str. 8, 79104 Freiburg, Germany; 3 Translational Research Center, University Hospital of Psychiatry, University of Bern, 3000 Bern, Switzerland; 4 Department of Psychology, University of Alberta, Edmonton, AB T6G 2E9 Canada

**Keywords:** EEG microstates, Retest reliability, Stability, Clustering, Fitting

## Abstract

EEG microstates represent functional brain networks observable in resting EEG recordings that remain stable for 40–120ms before rapidly switching into another network. It is assumed that microstate characteristics (i.e., durations, occurrences, percentage coverage, and transitions) may serve as neural markers of mental and neurological disorders and psychosocial traits. However, robust data on their retest-reliability are needed to provide the basis for this assumption. Furthermore, researchers currently use different methodological approaches that need to be compared regarding their consistency and suitability to produce reliable results. Based on an extensive dataset largely representative of western societies (2 days with two resting EEG measures each; day one: *n* = 583; day two: *n* = 542) we found good to excellent short-term retest-reliability of microstate durations, occurrences, and coverages (average ICCs = 0.874-0.920). There was good overall long-term retest-reliability of these microstate characteristics (average ICCs = 0.671-0.852), even when the interval between measures was longer than half a year, supporting the longstanding notion that microstate durations, occurrences, and coverages represent stable neural traits. Findings were robust across different EEG systems (64 vs. 30 electrodes), recording lengths (3 vs. 2 min), and cognitive states (before vs. after experiment). However, we found poor retest-reliability of transitions. There was good to excellent consistency of microstate characteristics across clustering procedures (except for transitions), and both procedures produced reliable results. Grand-mean fitting yielded more reliable results compared to individual fitting. Overall, these findings provide robust evidence for the reliability of the microstate approach.

**Supplementary Information:**

The online version contains supplementary material available at 10.1007/s10548-023-00982-9.

## Introduction

**Fig. 1:**
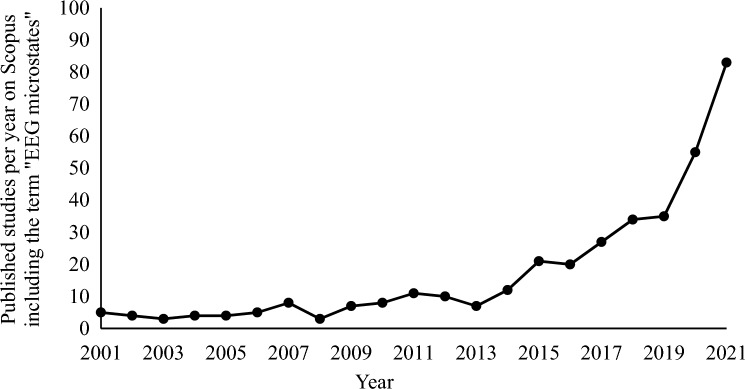
Number of newly published studies per year on Scopus including the term “EEG microstates”

Microstate analysis is a popular method used to investigate the temporal dynamics of large-scale brain networks on a millisecond scale using data obtained from multichannel electroencephalography (EEG; for a review, see Michel and Koenig 2018). Microstate networks show a temporal stability of approximately 40–120 ms before rapidly transitioning into other network types and can be reliably identified in resting EEG recordings. Typically, a small number of microstate types (often four to seven) explains the bulk of variance in the EEG (> 70%; e.g., Koenig et al. 2002). Microstate characteristics that are typically analyzed include the average duration, the average number of occurrences, and the percentage coverage of each microstate type, and transition probabilities between microstate types (for details, see methods). Even though the phenomenon of short-term stable topographic brain maps in the EEG has already been described more than 50 years ago by Lehmann (1971), the interest in this research field has recently increased significantly. E.g., the number of newly published studies per year including the term “EEG microstates” in their title, abstract, or keywords has increased by 137% within only two years (i.e., 2019–2021; see Fig. 1).

The figure shows the number of newly published studies per year on the online scientific search platform Scopus for the term “EEG microstates” in their title, abstract, or keywords for the years 2001 to 2021. Note that there is a substantial increase over the years, especially from 2019 onwards (2019: 35, 2020: 55, 2021: 83).

There are several reasons for this sudden success of the microstate approach. Recent publications demonstrate the potential of microstate research to contribute to a more sophisticated diagnosis, monitoring, prognosis, and prevention of mental disorders in clinical psychology and psychiatry. Microstate characteristics may serve as biomarkers of schizophrenia (da Cruz et al. 2020; de Bock et al. 2020), affective disorders (Al Zoubi et al. 2019; Damborská et al. 2019b; Murphy et al. 2020), anxiety disorders (Al Zoubi et al. 2019), ADHD (Férat et al. 2022a), and autism (D’Croz-Baron et al. 2019; Bochet et al. 2021). Facilitating the usage of microstate analysis in clinical settings, EEG systems are relatively cheap and easy to use (e.g., compared to fMRI) and increasingly mobile and quick to implement (e.g., Gargiulo et al. 2008; Askamp and van Putten 2014; Lau-Zhu et al. 2019). Moreover, microstates can be reliably identified in easy to measure, task-free resting EEG recordings of only three minutes (Liu et al. 2020), which is useful in clinical samples with physical or cognitive limitations, and in clinical settings where time is a valuable resource. Similarly, microstate characteristics associated with Parkinson’s disease (Chu et al. 2020), Alzheimer’s disease (Nishida et al. 2013; Tait et al. 2020), dementia (Nishida et al. 2013), stroke (Zappasodi et al. 2017), or multiple sclerosis (Gschwind et al. 2016) may serve as biomarkers in neurology.

Another issue in neuroscience is how stable human traits are represented in the brain (for a review, see DeYoung 2010). Research has demonstrated associations of microstate characteristics with the Big 5 personality traits (Zanesco et al. 2020), intelligence (Zappasodi et al. 2019; Liu et al. 2020), self-control (Kleinert et al. 2022), aggression (Kleinert and Nash 2022), religious belief (Nash et al. 2022a), and prosocial attitudes (Schiller et al. 2020). Additionally, microstate characteristics seem to be heritable as they were found to be similar in siblings (da Cruz et al. 2020), further supporting the notion that they may relate to interindividual differences. Thus, microstate analysis has emerged as a promising tool to study trait-related functions of the human brain. Another advance in microstate research is the increasing number of freely available tools to conduct microstate analyses, including the microstate toolbox for EEGLAB (Koenig 2017), CARTOOL (Brunet et al. 2011), RAGU (Koenig et al. 2011), or the Python library Pycrostates (Férat et al. 2022b), enabling standardized microstate analyses based on both resting state and event-related EEG data (for examples of event-related microstate analysis, see Schiller et al. 2016; Nash et al. 2022b).

Despite the widespread success of the microstate approach, there are two important shortcomings limiting its validity, both from a theoretical and methodological perspective. First, both clinical and basic research builds on the theoretical assumption that individuals show similar microstate characteristics over time, i.e., that microstates represent neural traits. To date, three studies have shown moderate to excellent retest-reliability of microstate characteristics (Khanna et al. 2014; Liu et al. 2020; Antonova et al. 2022). However, these studies were characterized by relatively small and non-representative samples of young participants and demonstrated retest-reliability only across short intervals (Antonova et al. 2022: *n* = 20, age: *M* = 31.5 years, *SD* = 12.5, interval: < 1 h; Khanna et al. 2014: *n* = 10, age: *M* = 30 years, *SD* = 10, interval: ≥ 48 h; Liu et al. 2020: *n* = 53, age: *M* = 23, *SD* = 2.4, interval: 1 day). During the revision stage of the current article, another study on the retest-reliability of microstate characteristics was published (Popov et al. 2023), showing mostly poor retest-reliability in 95 young adults and 93 older adults (interval: 7–9 days), calling into question previous results. Thus, robust data on the long-term retest-reliability of microstate characteristics over weeks and months are needed that confirm the theory that microstate characteristics represent stable neural traits. Second, microstate analyses are often applied differently across studies and labs, limiting the comparability of results. More specifically, varied clustering and fitting procedures (for details, see Methods) are used to obtain microstate characteristics, and there is little evidence that these methods produce consistent results.

In the current study, we aim to close these research gaps using a large dataset collected in the *Dortmund Vital Study* (DVS; Gajewski et al. 2022). In the DVS, data were collected on two days (day one [*n* = 583] and day two [*n* = 542]; average interval of 63 days), with two resting EEG measures on each day (i.e., a pre-measure at the beginning of each experimental session, and a post-measure after each session; average interval of 138 min on day one, and 99 min on day two). These data enable us to evaluate the short- and long-term retest-reliability of microstate characteristics in a sample that is largely representative of western societies (for details, see Gajewski et al. 2022). Based on previous studies in smaller samples (Khanna et al. 2014; Liu et al. 2020; Antonova et al. 2022), we derive five hypotheses: First, we expect to find at least moderate short-term retest-reliability of microstate durations, occurrences, and coverages. Although Popov and colleagues (2023) found poor retest-reliability of these characteristics, they used a different software for microstate analysis, leading to reduced comparability with our own study. Second, based on two studies in which transitions were analyzed (Liu et al. 2020; Antonova et al. 2022), we expect to find lower but still acceptable retest-reliability of transitions. Third, we expect at least moderate long-term retest-reliability of microstate durations, occurrences, coverages, and transitions, although it might be lower than the short-term retest-reliability. Based on considerable variability of the interval between day one and day two, we will also separately analyze the long-term retest-reliability of microstate characteristics in five groups with different intervals between measures (i.e., 1 week, 1 month, 1 to 3 months, 3 to 6 months, and more than six months). Khanna and colleagues (2014) systematically compared the retest-reliability of microstate characteristics obtained from different clustering procedures (k-means/AAHC) and fitting procedures (grand-mean fitting/individual fitting). Fourth, based on their findings, we expect to find equally good retest-reliability of microstate characteristics obtained from k-means clustering and AAHC, but superior retest-reliability of microstate characteristics obtained from grand-mean fitting compared to individual fitting (for details on these different methodologies, see methods). Relatedly, we will analyze the consistency of microstate characteristics across different methodologies. We will investigate the consistency of clustering procedures by testing for associations of microstate characteristics obtained from k-means clustering and AAHC. In an analogous fashion, we will investigate the consistency of fitting procedures by testing for associations of microstate characteristics obtained from grand-mean fitting and individual fitting. Fifth, based on Khanna and colleagues (2014), we expect high consistency of clustering procedures, but only moderate consistency of fitting procedures (due to the lower reliability of individual fitting compared to grand-mean fitting, especially concerning microstate coverages).

## Materials and Methods

### Data and Sample

The data for this study were collected in the context of the Dortmund Vital Study (DVS), an interdisciplinary, cross-sectional, and longitudinal study conducted by the Departments of Ergonomics, Psychology and Neurosciences, Immunology, and Toxicology of the Leibniz Research Centre for Working Environment and Human Factors (IfADo) in Dortmund, Germany (for a detailed description of the study, see Gajewski et al. 2022). The dataset of the study is largely representative of the German working population (20–70 years) regarding age, genetics, cognitive abilities, and employment (Gajewski et al. 2022). Compared to the population, there was a higher percentage of women in the DVS (61.5% vs 49.6%), and there were more participants with a university degree (41.6% vs 18.5%). The study was approved by the local ethics committee of the Leibniz Research Centre for Working Environment and Human Factors and conducted with the informed written consent of participants according to the principles expressed in the Declaration of Helsinki. The data and code of this study are freely available in the OSF repository (https://osf.io/hy8v7/).

Exclusion criteria of the DVS were neurological, cardiovascular, and oncological diseases, mental disorders (schizophrenia, severe depression, anxiety disorders), head injuries, severe eye diseases, accidents that limit physical fitness and mobility, usage of psychoactive drugs and medication, and limited vision and hearing after correction. At the time of this study, 609 participants completed the first experimental session on day one. For all analyses including only measures from day one, 26 participants were excluded because of problems during the measurement or bad EEG quality (as indicated by more than 50% data loss due to artifacts). resulting in a sample size of *n* = 583 (363 women, 220 men; age: *M* = 43.83 years, *SD* = 14.30). For all analyses including only measures from day two, a sample of *n* = 542 was available (334 women, 208 men; age: *M* = 43.85 years, *SD* = 14.30). Dropouts were due to canceled experimental sessions, individual changes related to exclusion criteria, pregnancy, and public restrictions or worries of participants related to the Covid 19 pandemic. The shared sample for analyses of the long-term retest-reliability of microstate characteristics across day one and day two was *n* = 525 (325 women, 200 men; age: *M* = 43.87 years, *SD* = 14.24).

### Procedure

Participants were recruited using online social media, flyers, newspaper advertisements, local print and radio media, and announcements during public events. Furthermore, several companies in the region informed their employees about the study. After registering for the DVS with a contact form, telephone interviews were conducted to inform participants about the study, check potential exclusion criteria, and assess demographic data. Prior to the first experimental session, participants completed a battery of questionnaires at home that are not relevant to this study.

Experimental sessions were run by professional laboratory staff members. In the first experimental session (day one), participants were seated in an electrically shielded EEG cabin, where they were equipped with a 64-electrode EEG system (Brain Products, Gilching, Germany). Three minutes of resting EEG were recorded with closed eyes, followed by three minutes of resting EEG with open eyes. Participants then completed a series of computerized tasks that are evaluated elsewhere (for details, see Gajewski et al. 2022). At the end of the experimental session, another, analogous resting EEG was recorded (average interval between measures: 138 min). Day two involved a procedure similar to day one (except for the fact that resting measures were recorded for two minutes instead of three minutes, and the eyes-open measures were conducted first, followed by the eyes-closed measures), using a 30-electrode EEG system (BioSemi B. V., Amsterdam, Netherlands). In accordance with standard procedures (Newson and Thiagarajan 2019) only eyes-closed periods were used for further analysis to avoid neural activation associated with subjective processing of visual cues. The average interval between the first and the second resting EEG measure on day two was 99 min. Only eyes-closed periods were used for further analyses. In total, participants received €160 for their participation (€100 for day one and €60 for day two).

#### EEG Recording and Preprocessing

On each day, resting EEG with closed eyes was recorded before and after completion of the test-battery. On day one, a 64-channel EEG system with Ag–AgCI active electrodes was used (actiCap; Brain Products, Gilching, Germany). The online sampling rate was 1000 Hz, the reference electrode was placed on position FCz, and the grounding electrode on position AFz. On day two, we used a 30-channel EEG system with Ag–AgCI active electrodes (BioSemi B. V., Amsterdam, Netherlands) with an online sampling rate of 2048 Hz. For grounding and online referencing, a common mode sense active electrode and a driven right leg passive electrode were used, together forming a feedback loop that drives the average potential. The reason for the usage of a different EEG system was to achieve comparability of day two measures with an earlier study using the same EEG setup. Both EEG systems were arranged on the scalp according to the extended 10–20 system, and impedances were below 10 kΩ.

EEG preprocessing was conducted in EEGLAB (Delorme and Makeig 2004). First, the data was down-sampled to 500 Hz (512 Hz on day two), and a band-pass filter of 2 to 20 Hz was applied (a frequency range commonly used in microstate research; e.g., Koenig et al. 2002). Second, large artifacts were removed automatically based on spectrum thresholding (EEGLAB function: pop_rejcont; recommended settings; frequency range: 15–30 Hz). Third, the EEG was re-derived to average reference (day one only, as this step is not necessary using the BioSemi EEG system). Fourth, we used the PrepPipeline to exclude noisy EEG channels (Bigdely-Shamlo et al. 2015). Fifth, we applied an additional artifact correction method (EEGLAB function: pop_autorej; recommended settings; threshold limit for detection of extremely large artifacts: 500 µV). Finally, an independent component analysis (ICA) was used to identify regular artifacts in the EEG data (EEGLAB function: pop_runica; recommended settings), followed by the rejection of components with a probability of more than 70% to reflect eye-movements or muscle artifacts (EEGLAB function: ICLabel; Pion-Tonachini et al. 2019).

#### EEG Microstate Analysis

Microstate characteristics were obtained using the microstate toolbox for EEGLAB by Koenig (2017; version 1.2). First, electric potential field maps were extracted from peaks of global field power for optimal signal-to-noise ratio. Second, these *individual maps* were submitted to a clustering procedure for the identification of *mean individual maps* using modified k-means clustering (k-means; Pascual-Marqui et al. 1995; Murray et al. 2008) or atomize and agglomerate hierarchical clustering (AAHC; Murray et al. 2008). In the modified k-means clustering procedure, a pre-defined number of *k* individual maps are randomly picked as cluster template maps. These template maps are then optimized to fit the data in an iterative process by repeatedly assigning all individual maps to the most similar template map, and then updating the template maps by the first principal component of the assigned individual maps, until a convergence criterion has been reached. Another option is the atomize and agglomerate hierarchical clustering procedure (AAHC; Murray et al. 2008), which starts with all individual maps being separate clusters, and then repeatedly dissolving the cluster that contributes least to the global explained variance, assigning the resulting unassigned individual maps to the most similar cluster map, and updating the cluster maps by computing the first principal component of their members. This process is repeated in an iterative way until a pre-defined number of maps has been reached. We used both methods to enable a systematic comparison of their consistency, and to compare their suitability to produce reliable results.

Third, mean individual maps were submitted to a second cluster analysis for the identification of the most predominant *grand-mean microstate maps* across the whole available sample in each of the eight conditions (i.e., day one/pre/k-means, day one/post/k-means, day two/pre/k-means, day two/post/k-means, day one/pre/AAHC, day one/post/ AAHC, day two/pre/ AAHC, day two/post/ AAHC), with the constraint that there is a one-to-one relationship among grand-mean microstate maps and mean individual map on the subject level. Note that there is an ongoing debate on the appropriate number of clusters that should be extracted in the second level clustering (e.g., Murray et al. 2008; Michel and Koenig 2018). As the focus of the current study was to investigate the reliability of microstate characteristics across different measures and methodologies, the main criterion for choosing a cluster number was the consistency of grand-mean microstate maps across conditions. We extracted four, five, six, and seven clusters in each condition. To assess the consistency of grand-means across conditions, we computed grand-grand-mean microstate maps across the eight grand-means for each cluster number. Then, we compared the average variance that could be explained by grand-mean microstates in grand-grand-mean microstates. On average, grand-means with five clusters showed the highest explained variance compared to four, six, and seven clusters (97.03% vs 93.16%, 89.42%, and 91.56%), attesting almost perfect consistency across conditions (see Table 1 for grand-mean microstate maps, and Table 2 for spatial correlations among microstate maps of the same type across conditions). Furthermore, all maps could be clearly assigned to previously identified microstate types (e.g., Custo et al. 2017; Zanesco et al. 2020; Férat et al. 2022c), which was not the case for four, six, and seven clusters (see Table S2, Table S3, and Table S4 in the supplementary material). Thus, we decided to conduct all further analyses using five microstate maps only.

In the fitting procedure, individual maps were assigned to a microstate type based on spatial correlations with grand-mean microstate maps. Time-points between GFP peaks were assigned to a microstate type using a nearest neighbor interpolation. We will refer to this procedure as *grand-mean fitting* (GM fitting). In an alternative procedure, mean individual maps (instead of individual maps) were assigned to a microstate type based on spatial correlations with grand-mean microstate maps. Then, these mean individual maps (instead of grand-mean microstate maps) were used to assign individual maps to a microstate type. We will refer to this procedure as *individual fitting* (Ind fitting). In both fitting procedures, temporal smoothing was performed according to the standard parameters of the EEGLAB microstate plugin (window size of 20 ms; non-smoothness penalty of one; Koenig 2017). Again, we used both methods to enable a systematic comparison of their consistency, and to compare their suitability to produce reliable results. The fitting procedure results in a continuous sequence of microstate maps for each individual. From these sequences, individual microstate characteristics were derived. *Durations* refer to the average duration of each microstate type in milliseconds, *occurrences* refer to the average number of occurrences of each microstate type per second, *coverages* refer to the percentage of the EEG covered by each microstate type, and *transitions* refer to transition probabilities from each microstate type to each other microstate type, which were computed as the percentage of observed transitions from one microstate type to another relative to expected transitions (transitions = [observed transitions per second − expected transitions per second]/expected transitions per second × 100; i.e., a value of 10 indicates that transitions from one microstate type to another occurred 10% more frequently than expected based on a microstate’s occurrence; also see Schiller et al. 2020). Additionally, type-independent mean microstate characteristics include the percentage of explained variance in individual EEG measures by all microstate types combined, mean durations across microstate types, mean occurrences across microstate types, and the mean global field power across microstate types (mean standard deviation of all channels from zero across the EEG).

### Statistical Analyses

As a prerequisite for the following analyses, we tested for the consistency of topographic microstate maps of the five different types (A, B, C, C′, D) by computing spatial correlations between maps of the same type obtained from eight different conditions (i.e., day one/pre/k-means, day one/post/k-means, day two/pre/k-means, day two/post/k-means, day one/pre/AAHC, day one/post/AAHC, day two/pre/AAHC, day two/post/AAHC). Furthermore, we analyzed the short- and long-term retest-reliability of microstate maps, and their methodological consistency across clustering procedures (k-means/AAHC). When correlating maps across day one and day two with different electrode configurations (see Figure S1 in the supplementary material), the 64-channel data was spatially resampled to the 30-channel data using spherical spline interpolation as implemented in EEGLAB.

In the following analyses, we calculated Intraclass Correlation Coefficients (ICCs; Gamer et al. 2012) to test for the retest-reliability and methodological consistency of microstate characteristics. ICCs smaller than .50, between .50 and .75, between .75 and .90, and larger than .90 were considered to reflect poor, moderate, good, and excellent reliability, respectively (Koo and Li 2016). For the sake of compactness and understandability, we also calculated average ICCs of microstate durations, occurrences, and coverages across microstate types, and average ICCs of microstate transition across transition types. To this end, we z-transformed ICCs using the algorithm by Fisher (1915), averaged the values, and back-transformed the z-values to average correlations. This is necessary, as correlation coefficients are not normally distributed. We then tested for significant differences between average ICCs obtained from different measures, clustering-, and fitting procedures using one-sided z-tests as described by Eid and colleagues (2011; 547–548). Briefly, this procedure z-transforms two correlation coefficients and tests their difference against the null hypothesis, also taking into account different sample sizes.

To test our hypotheses, we first analyzed the short-term retest-reliability of microstate characteristics by computing ICCs between microstate characteristics obtained from the day one pre-measure and the day one post-measure (average interval of 138 min) in each condition (i.e., k-means/GM fitting, k-means/Ind fitting, AAHC/GM fitting, AAHC/Ind fitting). We repeated this procedure using pre- and post-measures from day two (average interval of 99 min). Second, we analyzed the long-term retest-reliability of microstate characteristics across an average interval of 63 days by computing ICCs between microstate characteristics obtained from the day one pre-measure and the day two pre-measure in each condition. We repeated this procedure using post-measures from day one and day two. Note that we also used the results from both the short- and long-term retest-reliability analyses to assess the suitability of the different clustering procedures (k-means/AAHC) and fitting procedures (GM fitting/Ind fitting) to produce reliable results. Based on the large variability of intervals between day one and day two (1–996 days), we also conducted these analyses separately in five different groups (i.e., group 1: interval of 1–7 days [*n* = 143], group 2: interval of 8–30 days [*n* = 129], group 3: interval of 31–90 days [*n* = 142], group 4: interval of 91–180 days [*n* = 70], group 5: interval of 181 days and more [*n* = 41]). Third, we analyzed the methodological consistency of microstate characteristics across clustering procedures (k-means/AAHC) in each measurement (day one/pre, day one/post, day two/pre, day two/post; each for both fitting procedures). Fourth, we analyzed the methodological consistency of microstate characteristics across fitting procedures (GM fitting/Ind fitting) in each measurement (day one/pre, day one/post, day two/pre, day two/post; each for both clustering procedures). Note that age- and sex-differences of microstate characteristics are evaluated in another study.

## Results

### Descriptive Statistics

We computed EEG microstate characteristics in 16 conditions (all combinations of 2 days [day one/day two], two measurements [pre-measure/post-measure], two clustering procedures [kmeans/AAHC], and two fitting procedures [GM fitting/Ind fitting]). The grand-average of variance explained by all microstate types in the EEG across participants and conditions was 81.45%, which is closely in line with previous literature (e.g., Koenig et al. 2002). The grand-average of the total time available for analyses was 141.62 s on day one and 90.95 s on day two. The grand-average mean duration, mean occurrence, and mean global field power of microstates across microstate types, participants, and conditions was 63.25 ms, 16.95 occurrences per second, and 4.81 standard deviations, respectively (see Table S1 in the supplementary material for descriptive statistics of microstate characteristics in each condition).

### Grand-Mean Microstate Maps

We computed sets of grand-mean microstate maps with five clusters for each day (day one/day two), measurement (pre-measure/post-measure), and clustering procedure (k-means/AAHC). All sets of maps included a map with a left occipital to right frontal orientation, representing microstate type A (exception: left occipital to right temporal orientation in day two/post/k-means), a map with a right occipital to left frontal orientation, representing microstate type B, a map with an occipital to frontal orientation, representing microstate type C, a map with a central occipital to frontal orientation, representing microstate type C′, and a map with an occipital to frontocentral orientation, representing microstate type D (Table 1). Thus, all grand-mean microstate maps could be clearly assigned to normative microstate types known from the literature (e.g., Koenig et al. 2002; Custo et al. 2017; Zanesco et al. 2020; Férat et al. 2022c).

**Table 1.**
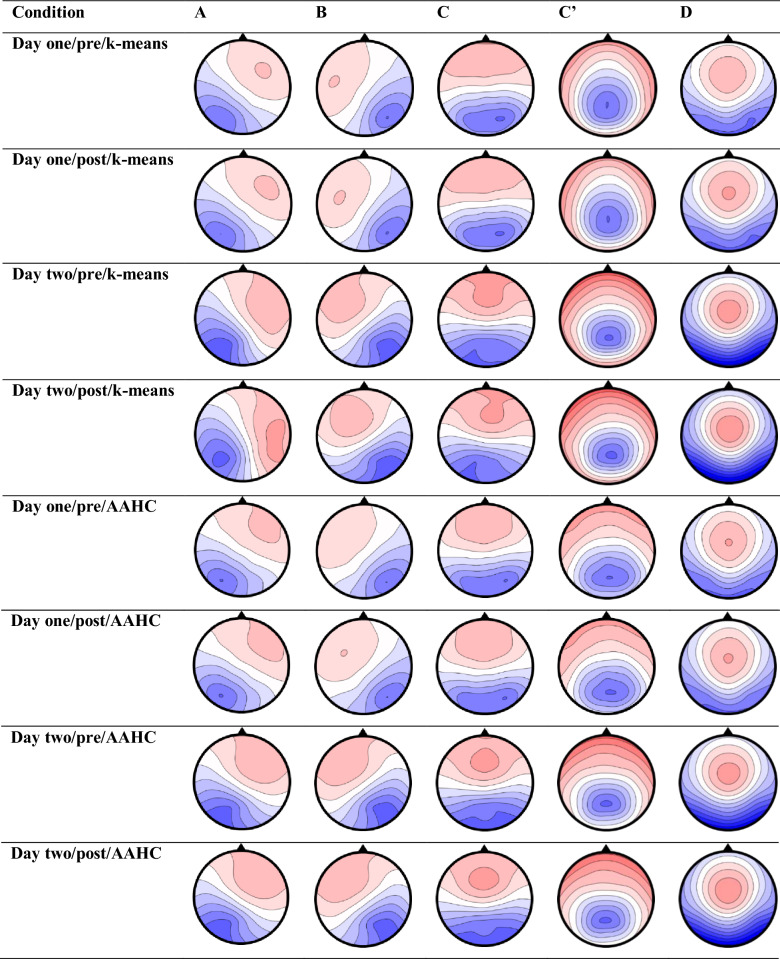
Grand-mean microstate maps

Grand-mean microstate maps (five clusters) for each day (day one/day two), measurement (pre/post), and clustering procedure (k-means/AAHC; eight conditions in total).

**Table 2.**
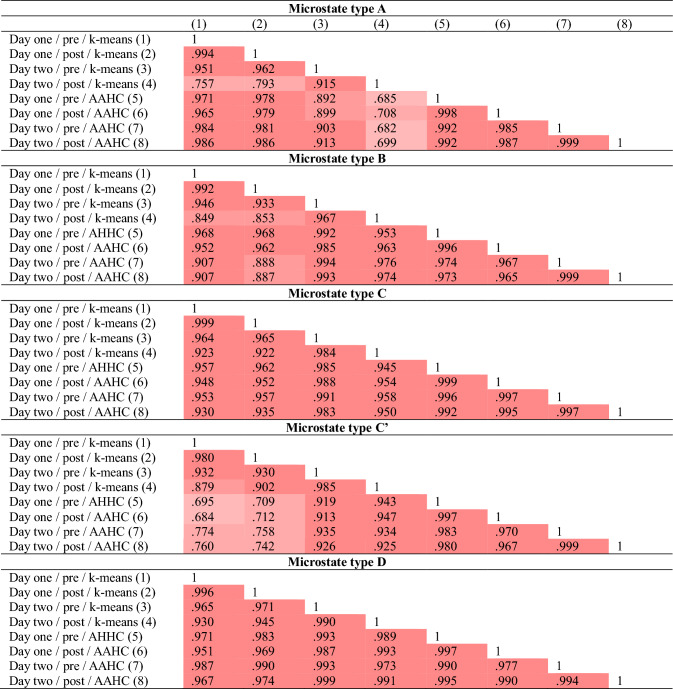
Spatial correlations of grand-mean microstate maps within each microstate type

Spatial correlations of grand-mean microstate maps within different microstate types (A, B, C, C′, D). Different shades of red indicate strengths of correlations (lighter shades indicate weaker correlations). On average, grand-mean microstate maps of type A, B, C, C′, and D correlated with *r* = .912, *r* = .953, *r* = .967, *r* = .885, and *r* = .980, respectively, across days (day one/day two), measurements (pre/post), and clustering procedures (k-means/AAHC)

### Short-Term Retest-Reliability

**Fig. 2:**
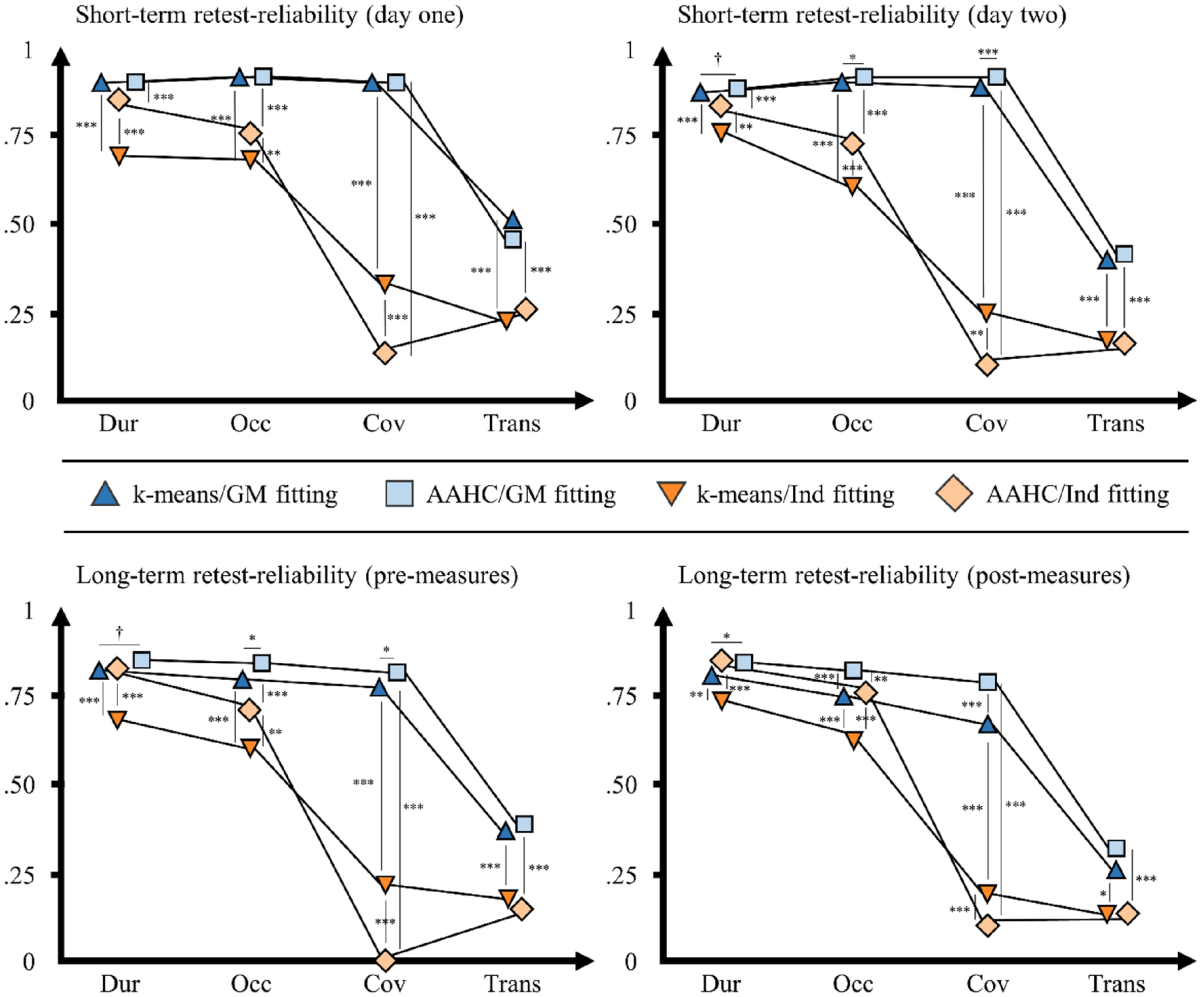
Average short- and long-term retest-reliability of microstate characteristics ***p < .001, **p < .010, *p < .050, †*p* < .10. y-axis: Intraclass correlation coefficient (ICC) scale ranging from zero to one. x-axis: Microstate characteristics (*Dur* durations, *Occ* occurrences, *Cov* coverages, *Trans* transitions). Legend: k-means = k-means clustering, *AAHC* atomize and agglomerate hierarchical clustering, GM fitting = GM fitting procedure, Ind fitting = Ind fitting procedure. Top: Average ICCs of microstate characteristics across microstate types (A, B, C, C′, D) showing their short-term retest-reliability on day one (top left; *N* = 583) and day two (top right; *N* = 542). Bottom: Average ICCs of microstate characteristics across microstate types (A, B, C, C′, D) showing their long-term retest-reliability using pre-measures (bottom left; *N* = 525) and post-measures (bottom right; *N* = 525). Stars (and crosses) indicate significant (and marginally significant) differences between average ICCs obtained from different methodologies as shown by z-tests. We analyzed differences of average ICCs between clustering procedures (k-means/GM fitting vs AAHC/GM fitting and k-means/Ind fitting vs AAHC/Ind fitting) and fitting procedures (k-means/GM fitting vs k-means/Ind fitting and AAHC/GM fitting vs AAHC/Ind fitting)

On day one, the short-term retest-reliability of microstate characteristics was calculated across an average interval of 137.69 min (*SD* = 8.24, range: 112.98–147.27). Microstate maps of all five microstate types showed excellent spatial retest-reliability across both clustering procedures (k-means/AAHC) as indicated by high spatial correlations between pre- and post-measures (see Table S5 in the supplementary material). Regarding temporal microstate characteristics, durations, occurrences, and coverages obtained from k-means clustering and GM fitting showed good to excellent average ICCs (see Fig. 2, see Table 3 for average ICCs and Table S6 in the supplementary material for type-specific ICCs). Compared to k-means clustering, AAHC yielded highly comparable results (all z-tests: *p* > .260). However, Ind fitting resulted in clearly inferior (poor to good) average ICCs compared to GM fitting both in k-means clustering and AAHC (all z-tests: *p* < .001). Notably, type-specific ICCs of coverages across all five microstate types and both clustering procedures were unacceptable using Ind fitting. Mean microstate characteristics (explained variance, mean duration, mean occurrence, mean GFP) showed good to excellent average ICCs across all clustering and fitting procedures. Microstate transitions showed similar (z-test: *p* = .138), poor to moderate average ICCs in both clustering procedures when using GM fitting, and similar (z-test: *p* = .306), poor, even lower average ICCs in both clustering procedures when using Ind fitting (both z-tests: *p* < .001).

On day two, the short-term retest-reliability was calculated across an average interval of 99.10 min (*SD* = 9.33, range: 55.10–136.47). Confirming results from day one, microstate maps showed excellent spatial retest-reliability (see Table S5 in the supplementary material). Durations, occurrences, and coverages obtained from k-means clustering and GM fitting showed good to excellent average ICCs (see Fig. 2, see Table 3 for average ICCs and Table S7 in the supplementary material for type-specific ICCs). Using AAHC instead of k-means clustering yielded comparable, marginally higher average ICCs compared to k-means clustering (z-tests for durations, occurrences, and coverages: *p* = .090, *p* = .023, and *p* = .002, respectively), whereas Ind fitting instead of GM fitting resulted in clearly inferior (poor to good) average ICCs both in k-means clustering and AAHC (all z-tests: *p* < .001). Mean microstate characteristics showed good to excellent retest-reliability across all clustering and fitting procedures. Microstate transitions showed similar (z-test: *p* = .325), poor to moderate retest-reliability in both clustering procedures when using GM fitting, and similar (z-test: *p* = .406), poor, even lower average ICCs in both clustering procedures when using Ind fitting (both z-tests: *p* < .001).

**Table 3.**
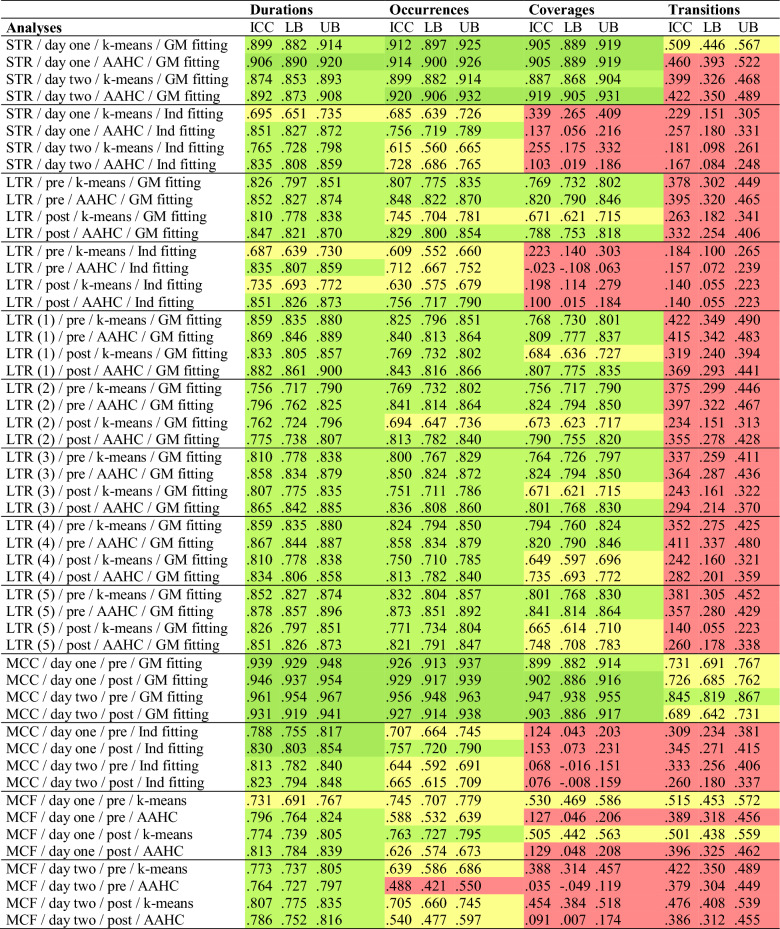
Average ICCs of durations, occurrences, coverages, and transitions for all analyses

*STR* Short-term retest-reliability (*n* = 583 for day one; *n* = 542 for day two), *LTR* long-term retest-reliability (*n* = 525), *MCC *methodological consistency of clustering procedures (*n* = 583 for day one; *n* = 542 for day two), *MCF* methodological consistency of fitting procedures (*n* = 583 for day one; *n* = 542 for day two). k-means = k-means clustering, *AAHC *atomize and agglomerate hierarchical clustering; GM fitting = GM fitting, Ind fitting = Ind fitting. Shown are average intraclass correlation coefficients (ICCs; model = two-way, type = agreement, alpha = .05; averaging according to Fisher’s (1915) algorithm) of durations, occurrences, and coverages across the five microstate types (A, B, C, C’, D), and average ICCs of transitions across the 20 transition types. Red (.00 < *ICC* < .50) = poor reliability; yellow (.50 < *ICC *< .75) = moderate reliability, light green (.75 < *ICC* < .90) = good reliability, dark green (*ICC* > .90) = excellent reliability

### Long-Term Retest-Reliability

The long-term retest-reliability of microstate characteristics was calculated across an average interval of 62.60 days (*SD* = 105.63, range: 1–996 days). Microstate maps of all five microstate types showed excellent spatial long-term retest-reliability across both clustering procedures (kmeans/AAHC) as indicated by high spatial correlations between day one and day two pre-measures (see Table S5 in the supplementary material). Regarding temporal microstate characteristics, durations, occurrences, and coverages obtained from k-means clustering and GM fitting showed moderate to good average ICCs (see Fig. 2, see Table 3 for average ICCs and Table S8 in the supplementary material for type-specific ICCs). Using AAHC instead of k-means clustering yielded somewhat higher, but comparable average ICCs (z-tests for durations, occurrences, and coverages: *p* = .078, *p* = .017, and *p* = .012, respectively). However, Ind fitting instead of GM fitting resulted in clearly inferior (poor to good) average ICCs in k-means clustering (all z-tests: *p* < .001) and AAHC (except for durations; z-tests for durations, occurrences, and coverages: *p* = .170, *p* < .001, and *p* < .001, respectively). Again, the type-specific ICCs of coverages across all five microstate types and both clustering procedures were unacceptable using Ind fitting. Mean microstate characteristics (explained variance, mean duration, mean occurrence, mean GFP) showed good to excellent retest-reliability across all clustering and fitting procedures. Microstate transitions showed similar (z-test: *p* = .373), mostly poor average ICCs in both clustering procedures when using GM fitting, and similar (z-test: *p* = .327), even lower average ICCs in both clustering procedures when using Ind fitting (both z-tests: *p* < .001).

Analogous analyses were calculated using post-measures of day one and day two. Supporting results from pre-measures, microstate maps showed excellent spatial retest-reliability (see Table S5 in the supplementary material). Durations, occurrences, and coverages obtained from k-means clustering and GM fitting showed mostly moderate to good average ICCs (see Fig. 2, see Table 3 for average ICCs and Table S9 in the supplementary material for type-specific ICCs). Using AAHC instead of k-means clustering yielded even higher average ICCs (z-tests for durations, occurrences, and coverages: *p* = .028, *p* < .001, and *p* < .001, respectively), whereas Ind fitting instead of GM fitting resulted in clearly inferior (poor to good) average ICCs both in k-means clustering (all z-tests: *p* ≤ .001) and AAHC (except for durations; z-tests of durations, occurrences, and coverages: *p* = .408, *p* = .001, and *p* < .001, respectively). Mean microstate characteristics showed good to excellent retest-reliability across all clustering and fitting procedures. Again, microstate transitions showed similar (z-test: *p* = .111), mostly poor retest-reliability in both clustering procedures when using GM fitting, and similar (z-test: *p* = .500), even lower retest-reliability in both clustering procedures when using Ind fitting (z-tests for k-means and AAHC: *p* = .019 and *p* < .001).

**Fig. 3:**
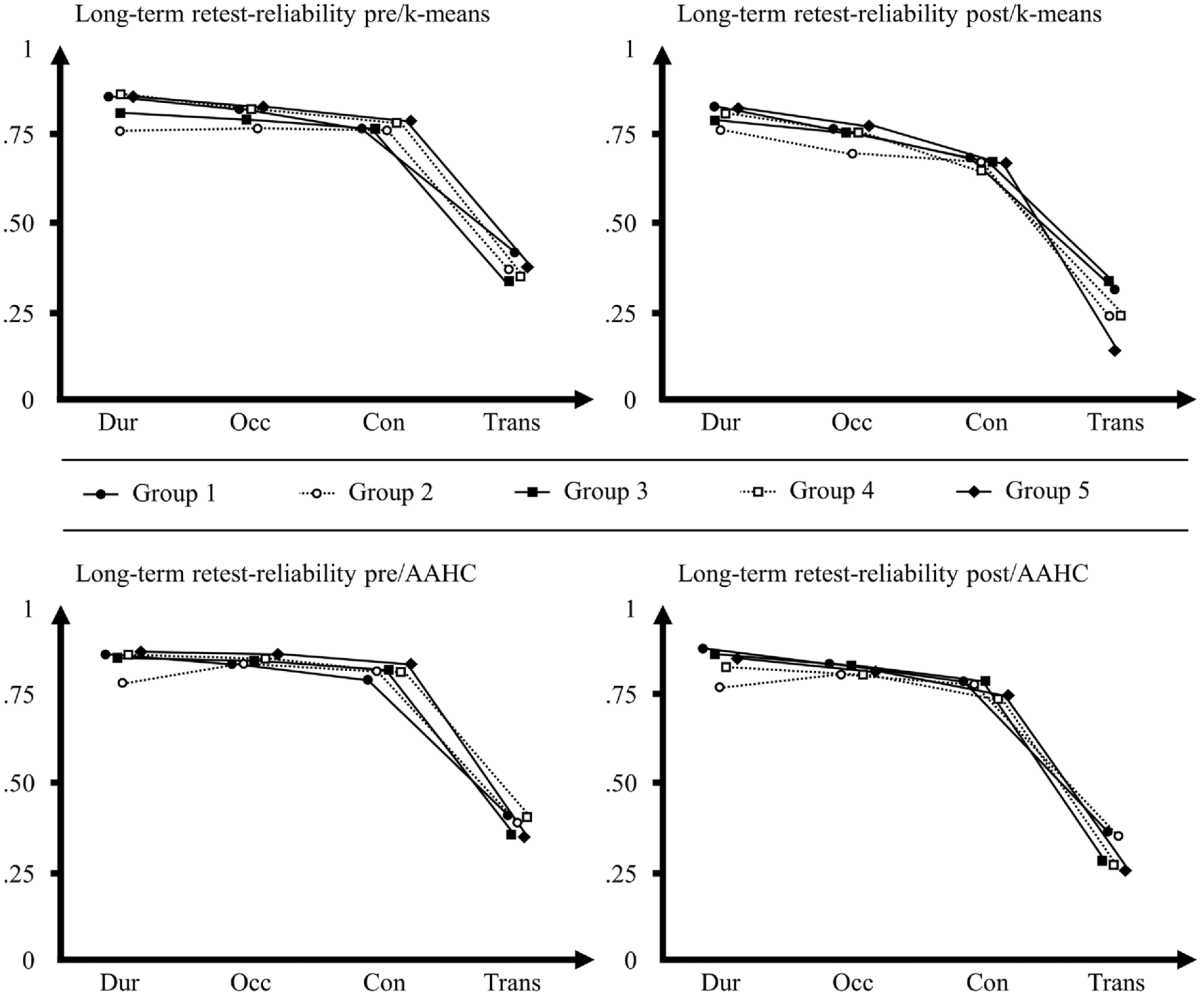
Average long-term retest-reliability of microstate characteristics in groups with different intervals between measures *n* = 525. y-axis: Intraclass correlation coefficient (ICC) scale ranging from zero to one. x-axis: Microstate characteristics (*Dur* durations, *Occ* occurrences, *Cov* coverages, *Trans* transitions). pre = pre-measures, post = post-measures, k-means = k-means clustering, *AAHC* atomize and agglomerate hierarchical clustering; Legend: Group 1 = interval of 1–7 days, Group 2: interval of 8–30 days, Group 3: interval of 31–90 days, Group 4: interval of 91–180 days, Group 5: interval of 181 days and more. Top: Average ICCs of microstate characteristics across types for each group, showing their long-term retest-reliability for pre-measures and k-means clustering (top left) and post-measures and k-means clustering (top right). Bottom: Average ICCs of microstate characteristics across types showing their long-term retest-reliability for pre-measures and AAHC (top left) and post-measures and AAHC (top right; see Table S15 in the supplementary material for information on group differences between average ICCs as indicated by z-tests). Notably, there was no systematic decrease of the retest-reliability with increasing intervals between day one and day two across all four conditions

Due to the considerable variability of the interval between day one and day two (1-996 days), we assigned participants to one out of five groups (group 1: interval of 1–7 days, group 2: interval of 8–30 days, group 3: interval of 31–90 days, group 4: interval of 91–180 days, group 5: interval of 181 days and more). Then, we separately analyzed the long-term retest-reliability of microstate characteristics in each of these groups (see Table 3 for average ICCs and Table S10, Table S11, Table S12, Table S13, and Table S14 in the supplementary material for type-specific ICCs in each group). Based on the clearly inferior retest-reliability of microstate characteristics obtained from Ind fitting compared to GM fitting, we only used GM fitting in these analyses. With just a few exceptions (6 out of 300 ICCs with poor reliability), ICCs of durations, occurrences, and coverages were moderate (72 out of 300 ICCs), good (210 out of 300 ICCs), or excellent (12 out of 320 ICCs) across all conditions (pre/k-means, post/k-means, pre/AAHC, post/AAHC), even in participants with an interval of more than 6 months between day one and day two. As in the whole sample, the retest-reliability of transitions was poor (329 out of 400 ICCs) to moderate (71 out of 400 ICCs) across groups and conditions. Notably, there was no systematic decrease of the long-term retest-reliability with increasing intervals between day one and day two (see Fig. 3; see Table S15 in the supplementary material for group-differences between average ICCs).

### Consistency Across Clustering Procedures

The consistency of microstate characteristics across the two clustering procedures (k-means/AAHC) was tested in each measurement (day one/pre, day one/post, day two/pre, day two/post). Microstate maps of all five microstate types showed good overall consistency across clustering procedures as indicated by high spatial correlations between maps obtained from k-means clustering and AAHC (see Table S5 in the supplementary material). As mentioned before, k-means clustering resulted in central to frontal orientations of the microstate type C′ map compared to a central occipital to frontal orientation in AAHC. For this reason, the C′ map from AAHC showed a stronger association with the C map from k-means clustering than the C′ map from k-means clustering in the day one/pre-measure (*r* = .695 vs *r* = .834) and the day one/post-measure (*r* = .712 vs *r* = .818). Furthermore, the A map of the day two/post-measure showed a left occipital to right temporal orientation in k-means clustering compared to a left occipital to right frontal orientation in all other measures, which is why the A map of this measure obtained from AAHC showed a stronger association with the C map from k-means clustering (*r* = .699 vs *r* = .837). Despite these minor inconsistencies, 17 out of 20 maps obtained from k-means clustering showed the strongest correlations with their counterparts obtained from AAHC (all correlations > 0.903).

Regarding temporal microstate characteristics, durations, occurrences, and coverages obtained from k-means clustering and GM fitting showed mostly excellent average ICCs compared to AAHC across days and measures (see Table 3 for average ICCs and Table S16 and Table S17 in the supplementary material for type-specific ICCs on day one and day two). However, Ind fitting resulted in clearly inferior (poor to good) average ICCs of clustering procedures compared to GM fitting in all measures (all z-tests: *p* < .001). The type-specific ICCs of coverages across all five microstate types and both clustering procedures were unacceptable using Ind fitting. Mean microstate characteristics (explained variance, mean duration, mean occurrence, mean GFP) showed good to excellent average ICCs across clustering procedures regardless of the fitting procedure used. Microstate transitions showed moderate to good average ICCs when using GM fitting and poor, significantly lower consistency when using Ind fitting (all z-tests: *p* < .001).

### Consistency Across Fitting Procedures

Durations, occurrences, coverages, and transitions obtained from GM fitting and Ind fitting after k-means clustering showed poor to good average ICCs across days and measures (see Table 3 for average ICCs and Table S18 and Table S19 for type-specific ICCs). Analogous analyses after AAHC resulted in comparable average ICCs regarding durations (z-tests for the day one/pre-measure, the day one/post-measure, the day two/pre-measure, and the day two/post-measure: *p* = .004, *p* = .036, *p* = .354, and *p* = .164, respectively), but inferior average ICCs regarding occurrences, coverages, and transitions (z-tests for occurrences, coverages, and transitions for the day one/pre-measure: *p* < .001, *p* < .001, and *p* = .003, respectively; z-tests for occurrences, coverages, and transitions for the day one/post-measure: *p* < .001, *p* < .001, and *p* = .012, respectively; z-tests for occurrences, coverages, and transitions for the day two/pre-measure: *p* < .001, *p* < .001, and *p* = .191, respectively; and z-tests for occurrences, coverages, and transitions for the day two/post-measure: *p* < .001, *p* < .001, and *p* = .030, respectively).

## Discussion

Our results show excellent consistency of topographic maps that can be assigned to the five microstate types A, B, C, C′, and D across four independent measures (day one/pre, day one/post, day two/pre, day two/post) and two clustering procedures (k-means/AAHC). Conversely, extracting four, six, or seven clusters yielded inconsistent combinations of microstate maps. This finding is in line with a recent study, which found that extracting five clusters that were very similar to ours, resulted in highly comparable microstate maps across different EEG frequency bands and behavioral conditions in a large sample of *n* = 203 (Férat et al. 2022c). Furthermore, previous studies that used an objective meta-criterion (available in the software CARTOOL; Brunet et al. 2011) often identified the same five clusters to optimally fit their data (e.g., Pascual-Marqui et al. 1995; Damborská et al. 2019a; Bréchet et al. 2020; Murphy et al. 2020; Zanesco et al. 2020; Bochet et al. 2021; D’Croz-Baron et al. 2021; Artoni et al. 2022). Based on earlier research (e.g., Koenig et al. 1999), four microstate maps (A, B, C, D) have been extracted from resting EEG data in most studies (for a review, see Michel and Koenig 2018). However, extracting four maps solely based on this tradition without considering objective criteria might not be the best choice, as a different number of clusters might explain the data better (Michel and Koenig 2018). In support of this view, Custo and colleagues (2017) found a considerable spatial correlation between microstate C and C′ (named F in that study) despite different neural generators, and argue that this might have caused previous studies to collapse these two maps into one, or mislabel them. Indeed, our study shows that C and C′ can be reliably identified as independent maps across measures, clustering procedures, and fitting procedures. Considering our own and the abovementioned findings, we recommend that data-driven criteria should be used to assess the optimal number of microstate maps. Regarding their spatial reliability, extracting five instead of four microstate maps seems to be superior. The grand-mean microstate maps identified in this study may be used as templates in future research.

The main goal of this study was to assess the short- and long-term retest-reliability of EEG microstate characteristics. As predicted, there was good to excellent short-term retest-reliability of microstate durations, occurrences, and coverages across an average interval of 138 min. This finding could be confirmed using two independent EEG measures from day two with an average interval of 99 min. Furthermore, there were mostly good long-term retest-reliability coefficients across an average interval of 63 days. Again, this finding could be confirmed using two independent EEG measures recorded after the test-batteries on each day. We also tested for the long-term retest-reliability of microstate characteristics in five groups with varying intervals between measures. Surprisingly, there were no systematic decreases in the retest-reliability with increasing intervals. Indeed, even participants with an interval of at least half a year between measures showed moderate to excellent retest-reliability of microstate durations, occurrences, and coverages. These results provide the first evidence of its kind for the longstanding notion that microstate dynamics represent stable neural traits (e.g., da Cruz et al. 2020; Murphy et al. 2020; Zanesco et al. 2020; Kleinert et al. 2022). The fact that microstate characteristics were as reliable after one week as they were after half a year but showed even higher within-session reliability on the same day, suggests that millisecond brain dynamics might change to a certain degree on a day-to-day basis, but remain highly stable in their basic structure over long time periods. Note that we found high retest-reliability across different EEG systems (64-electrode Brain Products system vs. 30-electrode BioSemi system), recording lengths (three minutes on day one vs. two minutes on day two), and cognitive states (before vs. after experimental sessions), further highlighting the robustness of our findings.

Contrary to our hypotheses, there was only poor to moderate retest-reliability of microstate transitions across all measures, clustering procedures, and fitting procedures (ICCs mostly < 0.500). Although previous studies also found lower retest-reliability of microstate transitions compared to durations, occurrences, and coverages (Liu et al. 2020; Antonova et al. 2022), these values were still within an acceptable range (ICCs mostly > 0.600). As these studies used different software to analyze microstates, differences might result from different computation models. Antonova and colleagues (2022) speculate that the poor retest-reliability of transition probabilities (even after less than 1 h) might be due to the low reliability of occurrences, which are used to compute transitions. However, this explanation does not hold in our study, as occurrences showed good reliability. Compared to durations, occurrences, and coverages, transitions represent more complex temporal dynamics of microstate syntax that involve (at least) two different microstate types (for an example, see Lehmann et al. 2005). Given that complexity naturally hinders reproducibility, the lower reliability of microstate transitions seems a logical consequence. Research focusing on microstate transitions should thus aim to use large samples. However moderate, it should be noted that specific transition types showed acceptable short- and long-term retest-reliability, even after more than half a year. Thus, some transition types might have a trait-like quality as well, even if their reliability was lower compared to more simple microstate dynamics such as durations, occurrences, and coverages.

As hypothesized, individual fitting produced less reliable results compared to grand-mean fitting in all conditions, especially regarding coverages (see Fig. 2). Thus, our study strongly supports prior findings by Khanna and colleagues (2014) but extends them to a much larger sample. These findings provide evidence that using predominant individual maps instead of grand-mean microstate maps as templates to assign individual maps to a microstate type results in considerable inconsistencies across individuals, and eventually lower reliability of microstate characteristics. Nevertheless, it should be noted that durations and occurrences obtained from individual fitting mostly showed moderate to good short- and long-term retest-reliability. Relatedly, there was mixed consistency of microstate characteristics across fitting procedures, especially regarding coverages, which was probably due to the lower reliability of individual fitting compared to grand-mean fitting. The main problem with the individual fitting procedure is that the mean individual maps used as templates show substantial differences between individuals, leading to inconsistencies in assigning individual EEG maps to microstate types and consequently low reliability. This problem also exists on a lower scale in the current study because different grand-means were used as templates in the different conditions. However, this problem can be considered to be relatively minor, as grand-means were almost perfectly consistent. If anything, inconsistencies might lead to an underestimation of the true retest-reliability of microstate characteristics. In sum, we therefore recommend using grand-mean fitting and not individual fitting to obtain reliable microstate characteristics in future studies, especially when microstate coverages are to be analyzed.

As expected, both k-means clustering and AAHC yielded good overall short- and long-term retest-reliability of microstate characteristics. Although k-means clustering is the more popular method, AAHC appeared to yield somewhat superior short- and long-term retest-reliability of microstate durations, occurrences, and coverages. However, there was excellent consistency of microstate durations, occurrences, and coverages across clustering procedures (also see Khanna et al. 2014), supporting the view that both methods are valid options.

A limitation of this study is that pre-measures were conducted in the morning, and post-measures in the early afternoon, after participants completed a cognitively demanding test battery. Thus, this study might underestimate the true short-term retest-reliability of microstate characteristics as post-measures were likely affected by cognitive depletion and tiredness. Another limitation is that we used different EEG systems (64 vs. 30 electrodes) and recording times (3 vs. 2 min) on day one and day two. Again, this means that this study might underestimate the true long-term retest-reliability of microstate characteristics due to systematic differences between EEG recordings. On the other hand, these differences attest to the reliability of our findings across different cognitive states, EEG systems, and recording times. Different recording lengths might be related to different levels of arousal, since participants may become drowsy even after short resting state measurements (e.g., Tagliazucchi and Laufs 2014). As we did not assess arousal levels, we could not control for this possible covariate. In addition, it should be noted that we performed microstate analysis using the EEGLAB plugin for microstates by Koenig (2017; version 1.2), so our findings may not generalize to microstate characteristics obtained with other software. In conclusion, this study provides robust evidence for the short-and long-term retest-reliability of EEG microstate characteristics, even after more than 6 months. Moreover, both k-means clustering and AAHC yielded reliable results, whereas grand-mean fitting yielded superior reliability compared to individual fitting. This is a crucial step forward for standardized microstate research and ultimately for the use of microstates as biomarkers in basic research as well as clinical settings.

### Supplementary Information

Below is the link to the electronic supplementary material.

Supplementary material 1 (DOCX 2332.1 kb)

## Data Availability

The data and code of this study are freely available in the OSF repository (https://osf.io/hy8v7/).
