# Supplementary material for: On the Reliability of the EEG Microstate Approach
Source: Brain Topogr. 2023 Jul 6;37(2):271–86. doi: 10.1007/s10548-023-00982-9 (PMC10884204; doi:10.1007/s10548-023-00982-9)
Supplement: Supplementary file 1 — Supplementary material 1 (DOCX 2332.1 kb) [file 10548_2023_982_MOESM1_ESM.docx]

**Supplementary Material**

**Table S1**

*Descriptive statistics of microstate characteristics*

|  | **Durations** | | | | | | | | | | | |
| --- | --- | --- | --- | --- | --- | --- | --- | --- | --- | --- | --- | --- |
|  | Duration A | | Duration B | | Duration C | | Duration C’ | | Duration D | | Mean Duration | |
|  | M | SD | M | SD | M | SD | M | SD | M | SD | M | SD |
| Day one / pre / k-means / GM fitting | 59.03 | 8.04 | 57.98 | 7.99 | 67.69 | 20.77 | 54.27 | 7.48 | 60.53 | 12.24 | 63.15 | 10.76 |
| Day one / pre / k-means / Ind fitting | 59.75 | 9.06 | 58.74 | 9.43 | 64.42 | 11.10 | 55.82 | 8.50 | 62.66 | 29.64 | 62.51 | 9.79 |
| Day one / pre / AAHC / GM fitting | 60.47 | 8.80 | 59.97 | 8.87 | 61.69 | 16.44 | 58.70 | 10.87 | 58.23 | 9.63 | 62.61 | 9.94 |
| Day one/pre/ AAHC/Ind fitting | 60.41 | 8.61 | 59.56 | 8.68 | 61.77 | 9.19 | 58.35 | 8.70 | 58.21 | 9.01 | 61.39 | 9.07 |
| Day one / post / k-means / GM fitting | 60.45 | 8.45 | 59.20 | 8.41 | 68.72 | 19.78 | 56.07 | 8.01 | 62.55 | 12.68 | 64.79 | 10.96 |
| Day one / post / k-means / Ind fitting | 61.24 | 9.39 | 60.65 | 14.78 | 66.36 | 11.89 | 57.32 | 8.58 | 62.75 | 10.89 | 64.15 | 10.05 |
| Day one / post / AAHC / GM fitting | 61.78 | 9.13 | 61.19 | 9.13 | 63.17 | 16.55 | 60.53 | 11.18 | 60.36 | 10.42 | 64.35 | 10.21 |
| Day one / post / AAHC / Ind fitting | 61.62 | 8.87 | 61.19 | 9.14 | 63.32 | 9.35 | 59.83 | 8.89 | 59.67 | 8.89 | 63.01 | 9.27 |
| Day two / pre / k-means / GM fitting | 57.10 | 7.40 | 58.72 | 8.51 | 70.53 | 18.95 | 55.81 | 9.74 | 57.96 | 10.31 | 63.55 | 10.44 |
| Day two / pre / k-means / Ind fitting | 58.04 | 8.65 | 60.14 | 10.17 | 66.27 | 10.80 | 56.87 | 8.98 | 58.73 | 10.50 | 62.57 | 9.53 |
| Day two / pre / AAHC / GM fitting | 60.35 | 8.86 | 60.02 | 8.70 | 60.95 | 13.64 | 59.10 | 12.04 | 58.94 | 10.61 | 62.76 | 9.42 |
| Day two / pre / AAHC / Ind fitting | 60.47 | 8.77 | 59.60 | 8.16 | 61.16 | 8.23 | 58.23 | 8.09 | 58.13 | 8.62 | 61.05 | 8.37 |
| Day two / post / k-means / GM fitting | 56.74 | 7.50 | 63.01 | 10.72 | 69.99 | 16.77 | 58.00 | 10.64 | 59.30 | 10.23 | 64.79 | 10.34 |
| Day two / post / k-means / Ind fitting | 57.84 | 8.44 | 63.84 | 10.50 | 67.50 | 11.52 | 58.86 | 9.94 | 59.69 | 10.31 | 64.23 | 9.76 |
| Day two / post / AAHC / GM fitting | 61.64 | 8.94 | 61.34 | 9.02 | 62.42 | 13.79 | 61.14 | 13.49 | 60.56 | 10.61 | 64.28 | 9.62 |
| Day two / post / AAHC / Ind fitting | 61.67 | 8.75 | 61.08 | 8.52 | 63.21 | 9.01 | 59.85 | 8.60 | 59.54 | 9.32 | 62.75 | 8.64 |
|  | **Occurrences** | | | | | | | | | | | |
|  | Occurrence A | | Occurrence B | | Occurrence C | | Occurrence C’ | | Occurrence D | | Mean Occurrence | |
|  | M | SD | M | SD | M | SD | M | SD | M | SD | M | SD |
|  | 3.37 | .795 | 3.28 | .738 | 3.75 | .549 | 3.06 | .829 | 3.63 | .769 | 17.09 | 2.26 |
| Day one / pre / k-means / GM fitting | 3.35 | .650 | 3.24 | .701 | 3.80 | .524 | 3.11 | .681 | 3.66 | .652 | 17.16 | 2.22 |
| Day one / pre / k-means / Ind fitting | 3.51 | .801 | 3.47 | .751 | 3.10 | .618 | 3.46 | .743 | 3.58 | .895 | 17.12 | 2.19 |
| Day one / pre / AAHC / GM fitting | 3.44 | .567 | 3.40 | .538 | 3.55 | .532 | 3.44 | .539 | 3.50 | .550 | 17.34 | 2.18 |
| Day one/pre/ AAHC/Ind fitting | 3.31 | .749 | 3.20 | .711 | 3.63 | .552 | 3.07 | .805 | 3.54 | .696 | 16.74 | 2.24 |
| Day one / post / k-means / GM fitting | 3.29 | .616 | 3.18 | .686 | 3.68 | .514 | 3.09 | .593 | 3.56 | .604 | 16.80 | 2.21 |
| Day one / post / k-means / Ind fitting | 3.44 | .762 | 3.39 | .712 | 3.01 | .591 | 3.40 | .706 | 3.50 | .850 | 16.75 | 2.18 |
| Day one / post / AAHC / GM fitting | 3.36 | .527 | 3.33 | .535 | 3.47 | .518 | 3.38 | .526 | 3.43 | .522 | 16.97 | 2.15 |
| Day one / post / AAHC / Ind fitting | 3.11 | .935 | 3.27 | .733 | 3.94 | .577 | 3.18 | .860 | 3.45 | .881 | 16.94 | 2.19 |
| Day two / pre / k-means / GM fitting | 3.11 | .700 | 3.32 | .648 | 3.97 | .514 | 3.24 | .719 | 3.44 | .759 | 17.08 | 2.14 |
| Day two / pre / k-means / Ind fitting | 3.45 | .904 | 3.44 | .778 | 3.08 | .614 | 3.47 | .847 | 3.58 | .916 | 17.01 | 2.06 |
| Day two / pre / AAHC / GM fitting | 3.42 | .538 | 3.40 | .536 | 3.54 | .532 | 3.48 | .537 | 3.49 | .503 | 17.33 | 2.04 |
| Day two / pre / AAHC / Ind fitting | 2.88 | .894 | 3.40 | .679 | 3.82 | .573 | 3.17 | .828 | 3.36 | .868 | 16.62 | 2.18 |
| Day two / post / k-means / GM fitting | 2.89 | .658 | 3.46 | .641 | 3.80 | .530 | 3.22 | .650 | 3.34 | .675 | 16.71 | 2.15 |
| Day two / post / k-means / Ind fitting | 3.37 | .856 | 3.39 | .761 | 3.03 | .605 | 3.36 | .813 | 3.51 | .900 | 16.65 | 2.08 |
| Day two / post / AAHC / GM fitting | 3.35 | .529 | 3.33 | .513 | 3.48 | .519 | 3.40 | .506 | 3.39 | .504 | 16.94 | 2.06 |
| Day two / post / AAHC / Ind fitting | 3.37 | .795 | 3.28 | .738 | 3.75 | .549 | 3.06 | .829 | 3.63 | .769 | 17.09 | 2.26 |
|  | **Coverages** | | | | | | | | | | | |
|  | Coverage A | | Coverage B | | Coverage C | | Coverage C’ | | Coverage D | | | |
|  | M | SD | M | SD | M | SD | M | SD | M | SD | | |
| Day one / pre / k-means / GM fitting | 19.40 | 4.68 | 18.54 | 4.23 | 24.32 | 7.38 | 16.35 | 4.85 | 21.39 | 5.92 | | |
| Day one / pre / k-means / Ind fitting | 19.44 | 3.70 | 18.44 | 3.79 | 23.47 | 3.14 | 16.93 | 3.63 | 21.72 | 4.19 | | |
| Day one / pre / AAHC / GM fitting | 20.60 | 4.80 | 20.24 | 4.51 | 18.97 | 7.01 | 19.90 | 5.67 | 20.29 | 5.61 | | |
| Day one/pre/ AAHC/Ind fitting | 20.08 | 2.45 | 19.60 | 2.47 | 21.21 | 2.71 | 19.40 | 2.46 | 19.71 | 2.74 | | |
| Day one / post / k-means / GM fitting | 19.42 | 4.46 | 18.42 | 4.07 | 23.89 | 7.12 | 16.89 | 4.88 | 21.37 | 5.44 | | |
| Day one / post / k-means / Ind fitting | 19.45 | 3.44 | 18.52 | 3.59 | 23.37 | 3.30 | 17.21 | 3.25 | 21.45 | 3.50 | | |
| Day one / post / AAHC / GM fitting | 20.62 | 4.61 | 20.07 | 4.22 | 18.76 | 6.67 | 20.07 | 5.36 | 20.48 | 5.53 | | |
| Day one / post / AAHC / Ind fitting | 19.99 | 2.39 | 19.68 | 2.43 | 21.16 | 2.45 | 19.51 | 2.41 | 19.67 | 2.18 | | |
| Day two / pre / k-means / GM fitting | 17.50 | 5.53 | 18.83 | 4.69 | 26.51 | 7.29 | 17.62 | 6.02 | 19.54 | 5.95 | | |
| Day two / pre / k-means / Ind fitting | 17.70 | 4.29 | 19.45 | 4.39 | 25.16 | 2.92 | 17.99 | 4.37 | 19.69 | 4.91 | | |
| Day two / pre / AAHC / GM fitting | 20.37 | 5.77 | 20.13 | 4.92 | 18.66 | 6.36 | 20.26 | 6.80 | 20.59 | 6.19 | | |
| Day two / pre / AAHC / Ind fitting | 20.04 | 2.72 | 19.65 | 2.50 | 20.97 | 2.56 | 19.64 | 2.56 | 19.70 | 2.58 | | |
| Day two / post / k-means / GM fitting | 16.05 | 5.09 | 20.83 | 5.03 | 25.51 | 6.31 | 18.14 | 6.12 | 19.46 | 5.83 | | |
| Day two / post / k-means / Ind fitting | 16.35 | 3.78 | 21.36 | 4.28 | 24.51 | 3.25 | 18.41 | 4.17 | 19.37 | 4.48 | | |
| Day two / post / AAHC / GM fitting | 20.27 | 5.49 | 20.19 | 4.79 | 18.69 | 6.13 | 20.16 | 6.83 | 20.69 | 6.10 | | |
| Day two / post / AAHC / Ind fitting | 19.96 | 2.54 | 19.66 | 2.45 | 21.22 | 2.48 | 19.69 | 2.49 | 19.47 | 2.55 | | |
|  | **Mean microstate characteristics** | | | | | | | | | | | |
|  | Explained variance | | Mean Duration | | Mean Occurrence | | Mean GFP | |  |  | | |
|  | M | SD | M | SD | M | SD | M | SD |  |  | | |
| Day one / pre / k-means / GM fitting | 79.50 | 5.50 | 63.15 | 10.76 | 17.09 | 2.26 | 4.67 | 1.75 |  |  | | |
| Day one / pre / k-means / Ind fitting | 81.99 | 4.78 | 62.51 | 9.79 | 17.16 | 2.22 | 4.70 | 1.79 |  |  | | |
| Day one / pre / AAHC / GM fitting | 78.48 | 5.87 | 62.61 | 9.94 | 17.12 | 2.19 | 4.72 | 1.78 |  |  | | |
| Day one/pre/ AAHC/Ind fitting | 81.13 | 4.68 | 61.39 | 9.07 | 17.34 | 2.18 | 4.82 | 2.37 |  |  | | |
| Day one / post / k-means / GM fitting | 81.10 | 4.30 | 64.79 | 10.96 | 16.74 | 2.24 | 4.77 | 1.89 |  |  | | |
| Day one / post / k-means / Ind fitting | 83.43 | 3.77 | 64.15 | 10.05 | 16.80 | 2.21 | 4.80 | 1.94 |  |  | | |
| Day one / post / AAHC / GM fitting | 80.06 | 4.73 | 64.35 | 10.21 | 16.75 | 2.18 | 4.82 | 1.92 |  |  | | |
| Day one / post / AAHC / Ind fitting | 82.57 | 3.68 | 63.01 | 9.27 | 16.97 | 2.15 | 4.86 | 1.98 |  |  | | |
| Day two / pre / k-means / GM fitting | 80.78 | 4.72 | 63.55 | 10.44 | 16.94 | 2.19 | 4.74 | 1.81 |  |  | | |
| Day two / pre / k-means / Ind fitting | 83.44 | 4.11 | 62.57 | 9.53 | 17.08 | 2.14 | 4.77 | 1.85 |  |  | | |
| Day two / pre / AAHC / GM fitting | 79.54 | 5.24 | 62.76 | 9.42 | 17.01 | 2.06 | 4.81 | 1.86 |  |  | | |
| Day two / pre / AAHC / Ind fitting | 82.46 | 4.04 | 61.05 | 8.37 | 17.33 | 2.04 | 4.86 | 1.93 |  |  | | |
| Day two / post / k-means / GM fitting | 81.45 | 4.03 | 64.79 | 10.34 | 16.62 | 2.18 | 4.87 | 1.91 |  |  | | |
| Day two / post / k-means / Ind fitting | 83.98 | 3.66 | 64.23 | 9.76 | 16.79 | 2.15 | 4.89 | 1.95 |  |  | | |
| Day two / post / AAHC / GM fitting | 80.20 | 4.62 | 64.28 | 9.62 | 16.65 | 2.08 | 4.94 | 1.96 |  |  | | |
| Day two / post / AAHC / Ind fitting | 83.02 | 3.60 | 62.75 | 8.64 | 16.94 | 2.06 | 4.98 | 2.01 |  |  | | |
|  | **Transitions from Microstate A to other microstate types** | | | | | | | | | | | |
|  | A to B | | A to C | | A to C‘ | | A to D | |  |  |  |  |
|  | M | SD | M | SD | M | SD | M | SD |  |  |  |  |
| Day one / pre / k-means / GM fitting | 4.07 | 1.07 | -6.41 | 9.75 | -.487 | 9.92 | 1.95 | 9.50 |  |  |  |  |
| Day one / pre / k-means / Ind fitting | .234 | 1.99 | -1.26 | 1.88 | .001 | 1.18 | 1.30 | 1.26 |  |  |  |  |
| Day one / pre / AAHC / GM fitting | 4.08 | 11.82 | -1.30 | 9.11 | -.535 | 11.27 | -1.14 | 1.49 |  |  |  |  |
| Day one/pre/ AAHC/Ind fitting | .551 | 12.14 | .599 | 9.36 | .185 | 12.92 | .151 | 9.72 |  |  |  |  |
| Day one / post / k-means / GM fitting | 3.73 | 9.57 | -5.40 | 1.73 | -.789 | 8.74 | 2.57 | 1.03 |  |  |  |  |
| Day one / post / k-means / Ind fitting | -.167 | 11.73 | -.358 | 11.69 | -.152 | 1.60 | 1.09 | 1.36 |  |  |  |  |
| Day one / post / AAHC / GM fitting | 4.66 | 1.68 | -.800 | 1.66 | -1.96 | 9.85 | -.067 | 1.11 |  |  |  |  |
| Day one / post / AAHC / Ind fitting | 1.47 | 11.89 | -.082 | 1.49 | -1.20 | 1.13 | 1.70 | 1.16 |  |  |  |  |
| Day two / pre / k-means / GM fitting | 2.39 | 11.83 | -6.85 | 11.98 | 1.04 | 11.70 | 2.16 | 11.15 |  |  |  |  |
| Day two / pre / k-means / Ind fitting | -2.15 | 12.34 | -.064 | 12.32 | .744 | 11.84 | .428 | 11.08 |  |  |  |  |
| Day two / pre / AAHC / GM fitting | 3.81 | 12.91 | 1.75 | 11.82 | -2.39 | 12.92 | -.042 | 13.10 |  |  |  |  |
| Day two / pre / AAHC / Ind fitting | 2.24 | 13.88 | 1.27 | 11.44 | -1.21 | 13.10 | .567 | 12.44 |  |  |  |  |
| Day two / post / k-means / GM fitting | 1.35 | 12.34 | -5.75 | 12.01 | 1.73 | 11.32 | 1.61 | 12.45 |  |  |  |  |
| Day two / post / k-means / Ind fitting | -1.88 | 11.83 | -1.03 | 11.52 | 2.30 | 11.52 | .334 | 11.51 |  |  |  |  |
| Day two / post / AAHC / GM fitting | 2.93 | 14.61 | 2.92 | 12.05 | -2.24 | 13.53 | 1.29 | 15.12 |  |  |  |  |
| Day two / post / AAHC / Ind fitting | 2.38 | 13.53 | 2.14 | 12.62 | -1.68 | 13.98 | 1.25 | 14.79 |  |  |  |  |
|  | **Transitions from Microstate B to other microstate types** | | | | | | | | | | | |
|  | B to A | | B to C | | B to C‘ | | B to D | |  |  |  |  |
|  | M | SD | M | SD | M | SD | M | SD |  |  |  |  |
| Day one / pre / k-means / GM fitting | 3.89 | 9.64 | -4.43 | 1.22 | -.015 | 9.30 | .024 | 9.18 |  |  |  |  |
| Day one / pre / k-means / Ind fitting | .817 | 1.46 | -.436 | 11.17 | -.151 | 9.53 | -.082 | 1.19 |  |  |  |  |
| Day one / pre / AAHC / GM fitting | 4.58 | 11.27 | .349 | 1.58 | -2.63 | 11.13 | -.680 | 9.96 |  |  |  |  |
| Day one/pre/ AAHC/Ind fitting | 2.79 | 11.81 | 1.10 | 1.27 | -2.59 | 11.34 | .982 | 1.71 |  |  |  |  |
| Day one / post / k-means / GM fitting | 4.41 | 9.63 | -5.22 | 1.08 | -.002 | 9.82 | .300 | 9.40 |  |  |  |  |
| Day one / post / k-means / Ind fitting | .767 | 11.45 | -1.35 | 1.45 | 1.02 | 9.78 | -.112 | 1.45 |  |  |  |  |
| Day one / post / AAHC / GM fitting | 5.62 | 11.24 | -.373 | 9.79 | -1.84 | 11.05 | -.947 | 9.82 |  |  |  |  |
| Day one / post / AAHC / Ind fitting | 2.87 | 12.74 | .891 | 1.86 | -.998 | 12.11 | -.367 | 1.45 |  |  |  |  |
| Day two / pre / k-means / GM fitting | 1.18 | 12.48 | -4.15 | 11.63 | -.763 | 1.95 | 1.19 | 1.87 |  |  |  |  |
| Day two / pre / k-means / Ind fitting | -2.17 | 12.53 | -.394 | 11.70 | .332 | 11.77 | .911 | 11.81 |  |  |  |  |
| Day two / pre / AAHC / GM fitting | 2.22 | 13.39 | 1.01 | 1.38 | -3.75 | 11.44 | -1.52 | 11.90 |  |  |  |  |
| Day two / pre / AAHC / Ind fitting | -.119 | 13.33 | 1.76 | 1.49 | -2.11 | 12.20 | -.029 | 11.65 |  |  |  |  |
| Day two / post / k-means / GM fitting | 1.05 | 12.26 | -4.01 | 11.59 | -.866 | 11.62 | 1.62 | 1.47 |  |  |  |  |
| Day two / post / k-means / Ind fitting | -.393 | 12.67 | -.004 | 12.20 | -.365 | 11.83 | .355 | 12.01 |  |  |  |  |
| Day two / post / AAHC / GM fitting | -.283 | 12.99 | -.083 | 1.11 | -1.43 | 12.02 | -2.01 | 11.96 |  |  |  |  |
| Day two / post / AAHC / Ind fitting | -1.74 | 13.39 | 1.35 | 1.65 | -.612 | 12.19 | -1.30 | 1.98 |  |  |  |  |
|  | **Transitions from Microstate C to other microstate types** | | | | | | | | | | | |
|  | C to A | | C to B | | C to C‘ | | C to D | |  |  |  |  |
|  | M | SD | M | SD | M | SD | M | SD |  |  |  |  |
| Day one / pre / k-means / GM fitting | -5.36 | 9.83 | -2.88 | 9.18 | -.531 | 11.00 | 1.14 | 1.79 |  |  |  |  |
| Day one / pre / k-means / Ind fitting | -2.48 | 11.41 | -1.35 | 1.82 | -1.30 | 1.59 | .384 | 9.09 |  |  |  |  |
| Day one / pre / AAHC / GM fitting | -4.07 | 9.62 | -3.62 | 9.32 | -2.69 | 11.32 | 3.65 | 9.15 |  |  |  |  |
| Day one/pre/ AAHC/Ind fitting | -2.42 | 9.69 | -3.52 | 9.46 | -2.79 | 9.49 | 2.28 | 1.85 |  |  |  |  |
| Day one / post / k-means / GM fitting | -3.56 | 9.72 | -3.45 | 9.97 | .005 | 1.36 | .238 | 1.65 |  |  |  |  |
| Day one / post / k-means / Ind fitting | -2.33 | 1.39 | -1.60 | 1.29 | -.815 | 1.02 | -.353 | 9.86 |  |  |  |  |
| Day one / post / AAHC / GM fitting | -4.04 | 8.80 | -3.14 | 9.63 | -.582 | 1.74 | 1.78 | 8.88 |  |  |  |  |
| Day one / post / AAHC / Ind fitting | -2.82 | 9.45 | -2.44 | 9.21 | -1.36 | 9.82 | .637 | 8.59 |  |  |  |  |
| Day two / pre / k-means / GM fitting | -4.77 | 12.55 | -3.93 | 11.65 | -.109 | 12.35 | -.438 | 12.67 |  |  |  |  |
| Day two / pre / k-means / Ind fitting | -1.77 | 11.98 | -1.66 | 11.93 | -.456 | 11.09 | -.471 | 11.18 |  |  |  |  |
| Day two / pre / AAHC / GM fitting | -3.94 | 1.79 | -3.93 | 9.81 | -1.11 | 1.39 | 1.03 | 1.83 |  |  |  |  |
| Day two / pre / AAHC / Ind fitting | -3.33 | 11.42 | -2.78 | 1.62 | -.692 | 11.00 | -.354 | 11.56 |  |  |  |  |
| Day two / post / k-means / GM fitting | -4.29 | 12.74 | -2.82 | 11.20 | .520 | 12.66 | -1.49 | 12.58 |  |  |  |  |
| Day two / post / k-means / Ind fitting | -2.51 | 11.95 | -.413 | 11.48 | -.645 | 11.48 | -1.71 | 11.42 |  |  |  |  |
| Day two / post / AAHC / GM fitting | -2.34 | 11.71 | -3.51 | 1.09 | -.212 | 1.88 | -1.13 | 1.89 |  |  |  |  |
| Day two / post / AAHC / Ind fitting | -2.72 | 12.71 | -1.36 | 1.32 | -.352 | 11.00 | -1.61 | 1.89 |  |  |  |  |
|  | **Transitions from Microstate C‘to other microstate types** | | | | | | | | | | | |
|  | C‘ to A | | C‘ to B | | C‘ to C | | C‘ to D | |  |  |  |  |
|  | M | SD | M | SD | M | SD | M | SD |  |  |  |  |
| Day one / pre / k-means / GM fitting | -.485 | 9.37 | -.894 | 8.86 | -3.24 | 1.42 | 1.11 | 11.39 |  |  |  |  |
| Day one / pre / k-means / Ind fitting | -.095 | 1.61 | -.510 | 1.49 | -.466 | 9.97 | 1.07 | 9.38 |  |  |  |  |
| Day one / pre / AAHC / GM fitting | 1.33 | 11.68 | -1.41 | 1.89 | .675 | 11.44 | .756 | 9.76 |  |  |  |  |
| Day one/pre/ AAHC/Ind fitting | 1.17 | 12.86 | -.046 | 12.15 | -.140 | 1.85 | -1.84 | 11.53 |  |  |  |  |
| Day one / post / k-means / GM fitting | -.792 | 8.78 | -.320 | 8.88 | -3.85 | 1.72 | .190 | 1.39 |  |  |  |  |
| Day one / post / k-means / Ind fitting | -.403 | 1.00 | .166 | 1.96 | -1.55 | 1.02 | -.985 | 1.96 |  |  |  |  |
| Day one / post / AAHC / GM fitting | .510 | 11.41 | -1.20 | 11.34 | .869 | 11.80 | -.138 | 11.76 |  |  |  |  |
| Day one / post / AAHC / Ind fitting | .974 | 11.48 | -.263 | 11.73 | .860 | 9.43 | 1.31 | 11.62 |  |  |  |  |
| Day two / pre / k-means / GM fitting | 1.70 | 11.08 | -1.72 | 1.72 | -4.37 | 12.00 | 2.09 | 11.83 |  |  |  |  |
| Day two / pre / k-means / Ind fitting | 1.30 | 11.13 | .142 | 12.15 | -1.65 | 12.03 | .295 | 11.00 |  |  |  |  |
| Day two / pre / AAHC / GM fitting | -.607 | 14.06 | -4.16 | 12.53 | 1.94 | 11.61 | .964 | 12.36 |  |  |  |  |
| Day two / pre / AAHC / Ind fitting | -1.46 | 13.31 | -1.04 | 18.43 | 2.30 | 11.36 | 1.03 | 1.89 |  |  |  |  |
| Day two / post / k-means / GM fitting | .647 | 11.83 | -1.25 | 1.79 | -5.10 | 12.33 | 2.17 | 12.79 |  |  |  |  |
| Day two / post / k-means / Ind fitting | .459 | 11.76 | .069 | 11.62 | -2.48 | 11.99 | -.571 | 12.32 |  |  |  |  |
| Day two / post / AAHC / GM fitting | -2.75 | 14.31 | -2.54 | 12.02 | 1.29 | 11.13 | 1.26 | 14.67 |  |  |  |  |
| Day two / post / AAHC / Ind fitting | -1.27 | 14.25 | -1.19 | 12.19 | 1.23 | 1.65 | .006 | 13.10 |  |  |  |  |
|  | **Transitions from Microstate D to other microstate types** | | | | | | | | | | | |
|  | D to A | | D to B | | D to C | | D to C‘ | |  |  |  |  |
|  | M | SD | M | SD | M | SD | M | SD |  |  |  |  |
| Day one / pre / k-means / GM fitting | 3.63 | 13.38 | .562 | 11.11 | .155 | 11.54 | 2.99 | 1.81 |  |  |  |  |
| Day one / pre / k-means / Ind fitting | 1.38 | 12.97 | -2.55 | 13.56 | 1.67 | 12.04 | -1.57 | 1.90 |  |  |  |  |
| Day one / pre / AAHC / GM fitting | 2.33 | 15.05 | -3.35 | 12.91 | .696 | 1.20 | -3.81 | 11.98 |  |  |  |  |
| Day one/pre/ AAHC/Ind fitting | 1.58 | 13.43 | .238 | 1.05 | 4.34 | 9.10 | -2.61 | 11.76 |  |  |  |  |
| Day one / post / k-means / GM fitting | .534 | 9.39 | -1.87 | 9.91 | 2.89 | 9.32 | .125 | 9.96 |  |  |  |  |
| Day one / post / k-means / Ind fitting | -3.11 | 1.02 | -.774 | 1.01 | -.825 | 1.79 | -1.91 | 1.30 |  |  |  |  |
| Day one / post / AAHC / GM fitting | -2.54 | 1.91 | .087 | 9.06 | 1.02 | 9.77 | -3.09 | 12.06 |  |  |  |  |
| Day one / post / AAHC / Ind fitting | 1.93 | 9.25 | .334 | 1.00 | 3.80 | 9.41 | -2.34 | 11.11 |  |  |  |  |
| Day two / pre / k-means / GM fitting | .798 | 9.81 | -2.81 | 9.37 | 2.54 | 9.23 | .194 | 13.26 |  |  |  |  |
| Day two / pre / k-means / Ind fitting | -1.93 | 9.56 | -1.64 | 9.96 | -2.78 | 12.41 | -1.47 | 11.78 |  |  |  |  |
| Day two / pre / AAHC / GM fitting | -1.22 | 9.74 | 1.40 | 11.18 | -.366 | 11.67 | -2.20 | 15.07 |  |  |  |  |
| Day two / pre / AAHC / Ind fitting | .541 | 1.66 | 1.47 | 12.15 | 4.40 | 11.24 | -1.97 | 15.29 |  |  |  |  |
| Day two / post / k-means / GM fitting | .493 | 11.84 | -.922 | 12.11 | 3.39 | 1.94 | -1.45 | 12.91 |  |  |  |  |
| Day two / post / k-means / Ind fitting | -3.29 | 12.53 | -.266 | 12.67 | -2.22 | 11.53 | -1.55 | 11.53 |  |  |  |  |
| Day two / post / AAHC / GM fitting | -1.81 | 13.08 | 1.75 | 11.69 | .970 | 11.52 | -2.27 | 13.19 |  |  |  |  |
| Day two / post / AAHC / Ind fitting | 1.03 | 11.21 | .585 | 12.48 | 3.65 | 9.56 | -1.66 | 12.75 |  |  |  |  |

*Note.* Day one: *n* = 583; Day two: *n* = 542. Shown are means (M) and standard deviations (SD) of microstate durations (in ms), occurrences (/s), coverages (%), and transitions (% of transitions that occurred more frequently than expected based on occurrences).

**Table S2**

*Grand-mean microstate maps (four clusters)*

| **Day one/pre/k-means** | 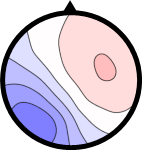 | 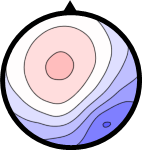 | 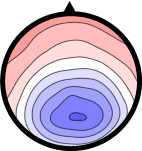 | 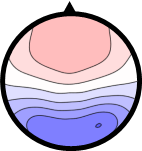 |
| --- | --- | --- | --- | --- |
| **Day one/post/k-means** | 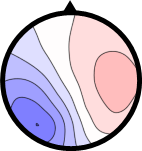 | 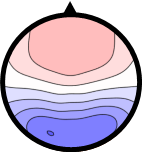 | 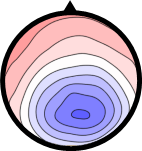 | 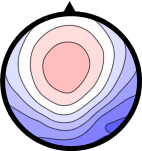 |
| **Day two/pre/k-means** | 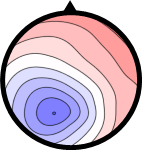 | 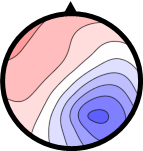 | 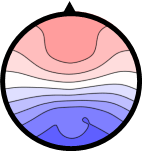 | 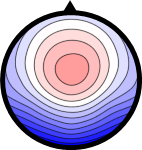 |
| **Day two/post/k-means** | 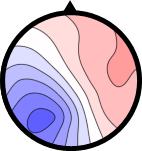 | 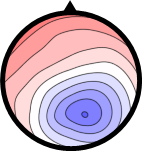 | 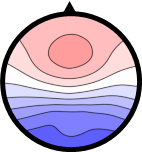 | 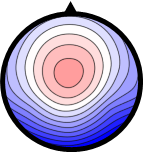 |
| **Day one/pre/AAHC** | 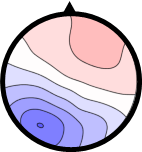 | 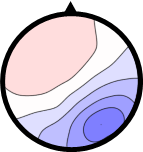 | 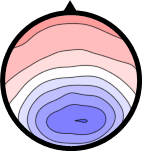 | 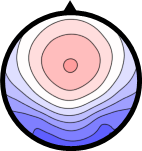 |
| **Day one/post/AAHC** | 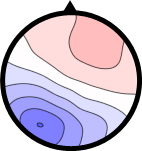 | 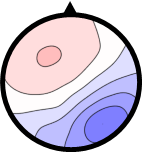 | 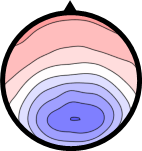 | 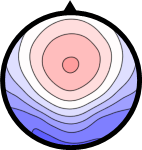 |
| **Day two/pre/AAHC** | 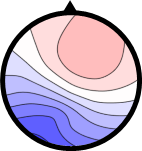 | 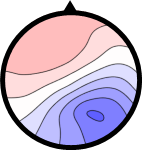 | 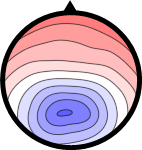 | 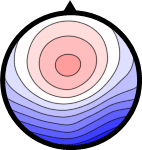 |
| **Day two/post/AAHC** | 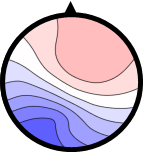 | 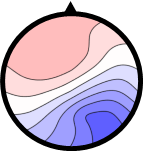 | 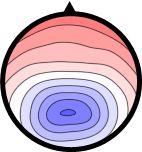 | 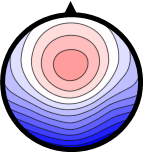 |

*Note.* Grand-mean microstate maps (four clusters) for each day (day one/day two), measurement (pre/post), and clustering procedure (k-means/AAHC; eight conditions in total). Note that there are inconsistencies in the topographies across conditions.

**Table S3**

*Grand-mean microstate maps (six clusters)*

| **Day one / pre / k-means** | | | | | |
| --- | --- | --- | --- | --- | --- |
| 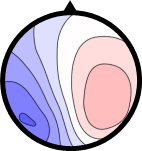 | 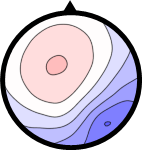 | 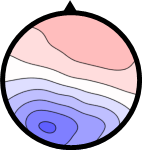 | 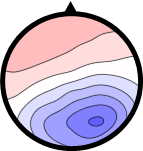 | 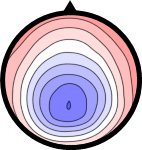 | 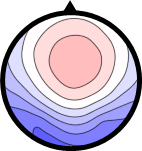 |
| **Day one / post / k-means** | | | | | |
| 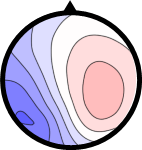 | 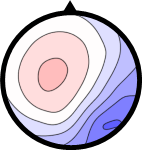 | 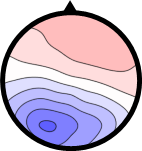 | 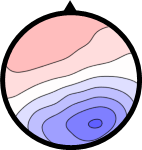 | 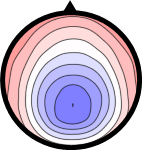 | 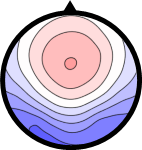 |
| **Day two / pre / k-means** | | | | | |
| 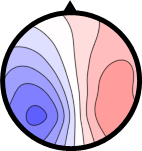 | 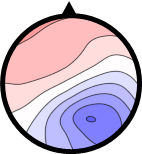 | 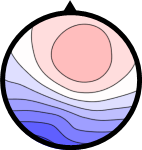 | 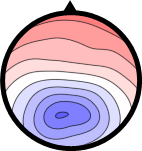 | 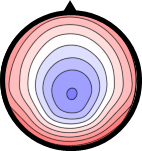 | 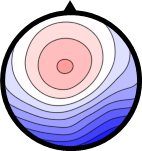 |
| **Day two / post / k-means** | | | | | |
| 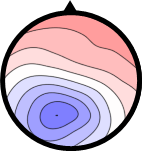 | 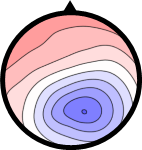 | 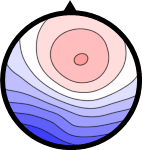 | 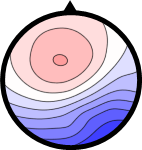 | 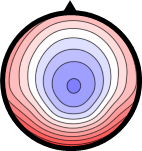 | 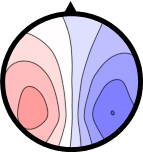 |
| **Day one / pre / AAHC** | | | | | |
| 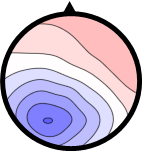 | 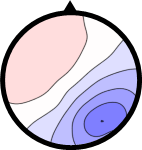 | 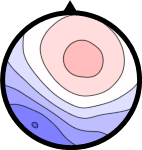 | 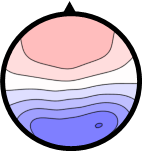 | 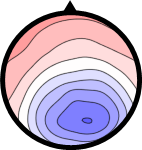 | 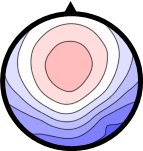 |
| **Day one /post / AAHC** | | | | | |
| 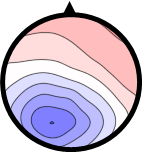 | 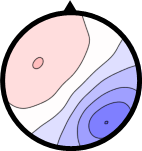 | 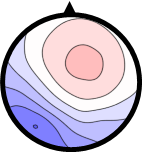 | 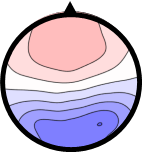 | 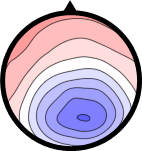 | 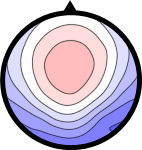 |
| **Day two / pre / AAHC** | | | | | |
| 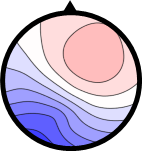 | 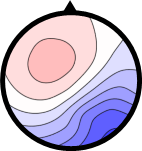 | 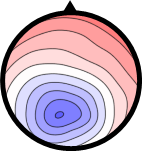 | 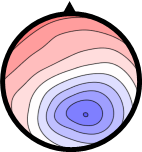 | 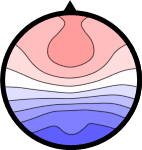 | 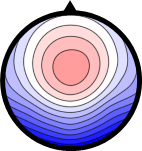 |
| **Day two / post / AAHC** | | | | | |
| 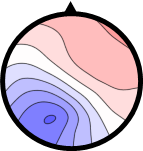 | 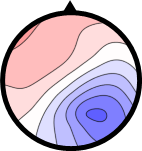 | 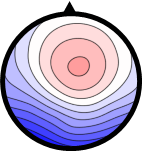 | 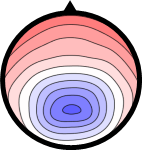 | 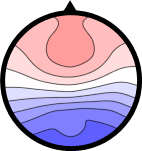 | 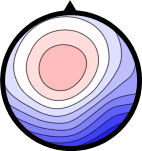 |

*Note.* Grand-mean microstate maps (six clusters) for each day (day one/day two), measurement (pre/post), and clustering procedure (k-means/AAHC; eight conditions in total). Note that there are inconsistencies in the topographies across conditions.

**Table S4**

*Grand-mean microstate maps (seven clusters)*

| **Day one / pre / k-means** | | | | | | |
| --- | --- | --- | --- | --- | --- | --- |
| 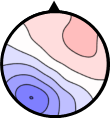 | 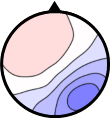 | 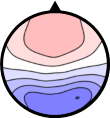 | 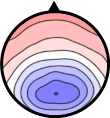 | 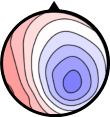 | 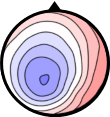 | 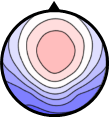 |
| **Day one / post / k-means** | | | | | | |
| 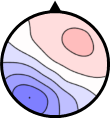 | 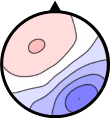 | 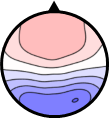 | 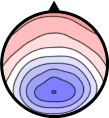 | 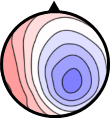 | 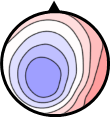 | 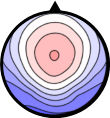 |
| **Day two / pre / k-means** | | | | | | |
| 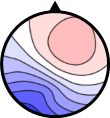 | 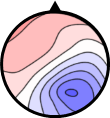 | 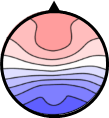 | 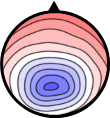 | 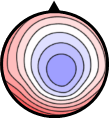 | 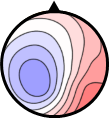 |  |
| **Day two / post / k-means** | | | | | | |
|  |  |  |  |  |  |  |
| **Day one / pre / AAHC** | | | | | | |
|  |  |  |  |  |  |  |
| **Day one /post / AAHC** | | | | | | |
|  |  |  |  |  |  |  |
| **Day two / pre / AAHC** | | | | | | |
|  |  |  |  |  |  |  |
| **Day two / post / AAHC** | | | | | | |
|  |  |  |  |  |  |  |

*Note.* Grand-mean microstate maps (seven clusters) for each day (day one/day two), measurement (pre/post), and clustering procedure (k-means/AAHC; eight conditions in total). Note that there are inconsistencies in the topographies across conditions.

**Table S5**

*Short- and long-term spatial retest-reliability and methodological consistency of microstate maps*

| **Short-term retest-reliability of microstate maps** | | | | | |
| --- | --- | --- | --- | --- | --- |
|  | MS A (post) | MS B (post) | MS C (post) | MS C’ (post) | MS D (post) |
| **Day one / pre / k-means and Day one / post / k-means** | | | | | |
| MS A (pre) | .994 | .023 | .430 | .102 | .350 |
| MS B (pre) | .013 | .992 | .357 | .003 | .368 |
| MS C (pre) | .423 | .398 | .999 | .285 | .606 |
| MS C’ (pre) | .024 | .063 | .301 | .980 | .012 |
| MS D (pre) | .448 | .272 | .566 | .017 | .996 |
| **Day one / pre / AAHC and Day one / post / AAHC** | | | | | |
| MS A (pre) | .998 | .017 | .553 | .409 | .353 |
| MS B (pre) | .031 | .996 | .592 | .334 | .397 |
| MS C (pre) | .609 | .547 | .999 | .633 | .668 |
| MS C’ (pre) | .353 | .400 | .646 | .997 | .085 |
| MS D (pre) | .396 | .301 | .603 | .069 | .997 |
| **Day two / pre / k-means and Day two / post / k-means** | | | | | |
| MS A (pre) | .915 | .059 | .199 | .115 | .051 |
| MS B (pre) | .033 | .967 | .713 | .264 | .571 |
| MS C (pre) | .570 | .452 | .984 | .463 | .559 |
| MS C’ (pre) | .115 | .389 | .516 | .985 | .013 |
| MS D (pre) | .263 | .312 | .512 | .000 | .990 |
| **Day two / pre / AAHC and Day two / post / AAHC** | | | | | |
| MS A (pre) | .999 | .149 | .669 | .502 | .474 |
| MS B (pre) | .159 | 1.00 | .711 | .475 | .464 |
| MS C (pre) | .681 | .710 | .997 | .624 | .756 |
| MS C’ (pre) | .526 | .475 | .685 | .999 | .161 |
| MS D (pre) | .446 | .412 | .641 | .099 | .994 |
| **Long-term retest-reliability of microstate maps** | | | | | |
|  | MS A (day two) | MS B (day two) | MS C (day two) | MS C’ (day two) | MS D (day two) |
| **Day one / pre / k-means and Day two / pre / k-means** | | | | | |
| MS A (day one) | .951 | .054 | .645 | .211 | .410 |
| MS B (day one) | .041 | .946 | .348 | .171 | .273 |
| MS C (day one) | .333 | .646 | .964 | .630 | .426 |
| MS C’ (day one) | .117 | .107 | .257 | .932 | .030 |
| MS D (day one) | .274 | .529 | .736 | .056 | .965 |
| **Day one / pre / AAHC and Day two / pre / AAHC** | | | | | |
| MS A (day one) | .992 | .167 | .685 | .570 | .435 |
| MS B (day one) | .082 | .974 | .597 | .322 | .458 |
| MS C (day one) | .658 | .737 | .996 | .680 | .696 |
| MS C’ (day one) | .438 | .564 | .688 | .983 | .164 |
| MS D (day one) | .485 | .392 | .650 | .108 | .990 |
| **Day one / post / k-means and Day two / post / k-means** | | | | | |
| MS A (day one) | .793 | .130 | .748 | .213 | .353 |
| MS B (day one) | .221 | .853 | .224 | .202 | .247 |
| MS C (day one) | .126 | .754 | .922 | .669 | .364 |
| MS C’ (day one) | .055 | .119 | .228 | .902 | .062 |
| MS D (day one) | .084 | .655 | .704 | .070 | .945 |
| **Day two / post / AAHC and Day two / post / AAHC** | | | | | |
| MS A (day one) | .987 | .150 | .647 | .600 | .347 |
| MS B (day one) | .082 | .965 | .620 | .302 | .461 |
| MS C (day one) | .666 | .712 | .995 | .681 | .642 |
| MS C’ (day one) | .392 | .602 | .635 | .967 | .108 |
| MS D (day one) | .465 | .338 | .656 | .096 | .990 |
| **Methodological consistency of microstate maps across clustering procedures** | | | | | |
|  | MS A (AAHC) | MS B (AAHC) | MS C (AAHC) | MS C’ (AAHC) | MS D (AAHC) |
| **Day one / pre / k-means and Day one / pre / AAHC** | | | | | |
| MS A (k-means) | .971 | .003 | .473 | .244 | .427 |
| MS B (k-means) | .000 | .968 | .403 | .279 | .206 |
| MS C (k-means) | .577 | .533 | .957 | .834 | .441 |
| MS C’ (k-means) | .158 | .024 | .131 | .695 | .061 |
| MS D (k-means) | .420 | .486 | .785 | .198 | .971 |
| **Day one / post / k-means and Day one / post / AAHC** | | | | | |
| MS A (k-means) | .979 | .002 | .477 | .226 | .364 |
| MS B (k-means) | .002 | .962 | .374 | .266 | .216 |
| MS C (k-means) | .546 | .552 | .952 | .818 | .409 |
| MS C’ (k-means) | .112 | .042 | .118 | .712 | .089 |
| MS D (k-means) | .402 | .480 | .780 | .169 | .969 |
| **Day two / pre / k-means and Day two / pre / AAHC** | | | | | |
| MS A (k-means) | .903 | .009 | .374 | .303 | .268 |
| MS B (k-means) | .112 | .994 | .649 | .397 | .453 |
| MS C (k-means) | .749 | .648 | .991 | .727 | .656 |
| MS C’ (k-means) | .296 | .341 | .425 | .935 | .021 |
| MS D (k-means) | .434 | .417 | .636 | .095 | .993 |
| **Day two / post / k-means and Day two / post / AAHC** | | | | | |
| MS A (k-means) | .699 | .029 | .134 | .194 | .057 |
| MS B (k-means) | .214 | .974 | .792 | .432 | .567 |
| MS C (k-means) | .837 | .523 | .950 | .730 | .568 |
| MS C’ (k-means) | .258 | .455 | .425 | .925 | .014 |
| MS D (k-means) | .422 | .317 | .613 | .069 | .991 |

*Note.* Top section (short-term retest-reliability): Spatial correlations of microstate maps obtained from pre- and post-measures, indicating excellent short-term retest-reliability of microstate maps across days (day one/day two) and clustering procedures (k-means/AAHC). Middle section (long-term retest-reliability): Spatial correlations of microstate maps obtained from day one and day two, indicating excellent long-term retest-reliability of microstate maps across measures (pre/post) and clustering procedures (k-means/AAHC). Bottom section (methodological consistency of clustering procedures): Spatial correlations of microstate maps obtained from k-means and AAHC clustering, indicating excellent methodological consistency of clustering procedures in each measurement (day one/pre, day one/post, day two/pre, day two/post).

**Table S6**

*Short-term retest-reliability of microstate characteristics across 138 minutes (day one)*

|  | **ICCs of day one pre-measures and day one post-measures** | | | | | | | | | | | |
| --- | --- | --- | --- | --- | --- | --- | --- | --- | --- | --- | --- | --- |
|  | **k-means** | | | | | | **AAHC** | | | | | |
| **Parameter** | **GM fitting** | | | **Ind fitting** | | | **GM fitting** | | | **Ind fitting** | | |
|  | ICC | LB | UB | ICC | LB | UB | ICC | LB | UB | ICC | LB | UB |
| Dur. A | .895^***^ | .863 | .918 | .800^***^ | .759 | .834 | .906^***^ | .881 | .924 | .847^***^ | .816 | .873 |
| Dur. B | .902^***^ | .877 | .922 | .465^***^ | .370 | .546 | .905^***^ | .882 | .922 | .840^***^ | .800 | .871 |
| Dur. C | .920^***^ | .906 | .932 | .866^***^ | .833 | .892 | .927^***^ | .913 | .939 | .851^***^ | .816 | .878 |
| Dur. C’ | .875^***^ | .820 | .909 | .800^***^ | .756 | .835 | .903^***^ | .873 | .924 | .862^***^ | .828 | .889 |
| Dur. D | .899^***^ | .871 | .921 | .261^***^ | .131 | .372 | .886^***^ | .841 | .916 | .852^***^ | .818 | .879 |
| Occ. A | .924^***^ | .910 | .936 | .697^***^ | .643 | .742 | .922^***^ | .907 | .934 | .784^***^ | .742 | .819 |
| Occ. B | .921^***^ | .904 | .934 | .658^***^ | .598 | .710 | .919^***^ | .901 | .934 | .702^***^ | .648 | .747 |
| Occ. C | .885^***^ | .834 | .917 | .635^***^ | .560 | .696 | .898^***^ | .870 | .919 | .775^***^ | .730 | .812 |
| Occ. C’ | .916^***^ | .901 | .928 | .725^***^ | .676 | .766 | .898^***^ | .879 | .913 | .735^***^ | .687 | .775 |
| Occ. D | .911^***^ | .890 | .927 | .701^***^ | .644 | .748 | .928^***^ | .914 | .940 | .775^***^ | .732 | .811 |
| Cov. A | .909^***^ | .893 | .922 | .201^**^ | .060 | .321 | .912^***^ | .897 | .925 | .132^*^ | -.021 | .263 |
| Cov. B | .898^***^ | .880 | .913 | .234^***^ | .098 | .349 | .892^***^ | .873 | .908 | .077 | -.086 | .216 |
| Cov. C | .921^***^ | .906 | .933 | .420^***^ | .317 | .507 | .920^***^ | .906 | .932 | .128^*^ | -.026 | .259 |
| Cov. C’ | .899^***^ | .878 | .915 | .463^***^ | .369 | .544 | .898^***^ | .880 | .913 | .196^**^ | .054 | .317 |
| Cov. D | .894^***^ | .875 | .910 | .356^***^ | .243 | .453 | .898^***^ | .880 | .914 | .152^*^ | .002 | .279 |
| Exp. Var. | .835^***^ | .724 | .892 | .838^***^ | .718 | .897 | .857^***^ | .765 | .905 | .833^***^ | .707 | .893 |
| Mean Dur. | .936^***^ | .913 | .952 | .939^***^ | .912 | .956 | .934^***^ | .904 | .953 | .927^***^ | .896 | .947 |
| Mean Occ. | .943^***^ | .919 | .958 | .943^***^ | .916 | .959 | .940^***^ | .910 | .957 | .936^***^ | .905 | .954 |
| Mean GFP | .966^***^ | .960 | .972 | .963^***^ | .956 | .969 | .967^***^ | .960 | .972 | .853^***^ | .827 | .875 |
| A to B | .490^***^ | .400 | .566 | .255^***^ | .123 | .367 | .466^***^ | .372 | .546 | .405^***^ | .300 | .495 |
| A to C | .588^***^ | .515 | .650 | .215^**^ | .077 | .333 | .537^***^ | .455 | .607 | .136^*^ | -.016 | .265 |
| A to C’ | .337^***^ | .220 | .436 | .146^*^ | -.005 | .273 | .411^***^ | .307 | .499 | .212^**^ | .072 | .330 |
| A to D | .518^***^ | .433 | .590 | .213^**^ | .076 | .330 | .354^***^ | .240 | .451 | .322^***^ | .203 | .424 |
| B to A | .473^***^ | .381 | .552 | .277^***^ | .149 | .386 | .456^***^ | .360 | .538 | .276^***^ | .148 | .385 |
| B to C | .646^***^ | .584 | .699 | .264^***^ | .134 | .374 | .560^***^ | .483 | .626 | .316^***^ | .195 | .418 |
| B to C’ | .299^***^ | .176 | .404 | -.029 | -.210 | .124 | .328^***^ | .210 | .429 | .054 | -.112 | .195 |
| B to D | .406^***^ | .301 | .495 | .160^*^ | .012 | .285 | .326^***^ | .207 | .427 | .354^***^ | .239 | .451 |
| C to A | .577^***^ | .502 | .641 | .201^**^ | .060 | .321 | .431^***^ | .329 | .517 | .269^***^ | .139 | .379 |
| C to B | .474^***^ | .381 | .553 | .100 | -.059 | .235 | .362^***^ | .249 | .458 | .249^***^ | .116 | .361 |
| C to C’ | .600^***^ | .528 | .661 | .421^***^ | .318 | .507 | .544^***^ | .463 | .612 | .273^***^ | .145 | .383 |
| C to D | .612^***^ | .533 | .677 | .314^***^ | .194 | .417 | .525^***^ | .441 | .596 | .208^**^ | .069 | .327 |
| C’ to A | .332^***^ | .215 | .432 | .237^***^ | .103 | .352 | .363^***^ | .250 | .459 | .151^*^ | .001 | .279 |
| C’ to B | .392^***^ | .284 | .483 | .156^*^ | .006 | .283 | .385^***^ | .277 | .478 | .155^*^ | .006 | .282 |
| C’ to C | .703^***^ | .650 | .747 | .267^***^ | .138 | .377 | .582^***^ | .508 | .645 | .253^***^ | .121 | .365 |
| C’ to D | .595^***^ | .524 | .656 | .368^***^ | .256 | .463 | .617^***^ | .549 | .674 | .336^***^ | .219 | .436 |
| D to A | .355^***^ | .242 | .452 | .221^**^ | .085 | .337 | .261^***^ | .131 | .372 | .271^***^ | .142 | .380 |
| D to B | .360^***^ | .247 | .456 | .073 | -.090 | .213 | .408^***^ | .303 | .496 | .275^***^ | .146 | .384 |
| D to C | .646^***^ | .584 | .699 | .349^***^ | .234 | .447 | .588^***^ | .515 | .649 | .240^***^ | .105 | .354 |
| D to C’ | .580^***^ | .506 | .643 | .327^***^ | .208 | .428 | .570^***^ | .494 | .635 | .350^***^ | .235 | .448 |

*Note.* *n* = 583; ^***^ = *p* < .001, ^**^ = *p* < .010, ^*^ = *p* < .050, ^†^ = *p* < .10; k-means = k-means clustering, AAHC = atomize and agglomerate hierarchical clustering; GM fitting = grand-mean fitting, Ind fitting = individual fitting; ICC = Intraclass correlation coefficient (model = two-way, type = agreement, alpha = .05), LB = lower bound of 95% confidence interval, UB = upper bound of 95% confidence interval. Microstate characteristics: Dur. X = mean duration of microstate types, Occ. X = mean occurrences of microstate types, Cov. X = percentage coverages of microstate types, Exp. Var. = total explained variance by all microstate types, Mean Dur. = mean duration of all microstate types per second, Mean Occ. = mean occurrences of all microstate types per second, Mean GFP = mean global field power in standard deviations, X to X = transitions between microstate types. Shown are short-term retest-reliabilities of EEG microstate characteristics across two resting EEG measures on day one (ICCs of pre- and post-measures; average interval = 138 minutes). Red (.00 < *ICC* < .50) = poor reliability; yellow (.50 < *ICC* < .75) = moderate reliability, light green (.75 < *ICC* < .90) = good reliability, dark green (*ICC* > .90) = excellent reliability.

**Table S7**

*Short-term retest-reliability of microstate characteristics across 99 minutes (day two)*

|  | **ICCs of day two pre-measures and day two post-measures** | | | | | | | | | | | |
| --- | --- | --- | --- | --- | --- | --- | --- | --- | --- | --- | --- | --- |
|  | **k-means** | | | | | | **AAHC** | | | | | |
| **Parameter** | **GM fitting** | | | **Ind fitting** | | | **GM fitting** | | | **Ind fitting** | | |
|  | ICC | LB | UB | ICC | LB | UB | ICC | LB | UB | ICC | LB | UB |
| Dur. A | .859^***^ | .834 | .881 | .764^***^ | .721 | .801 | .879^***^ | .850 | .901 | .831^***^ | .797 | .860 |
| Dur. B | .821^***^ | .573 | .904 | .730^***^ | .602 | .807 | .887^***^ | .859 | .909 | .835^***^ | .794 | .866 |
| Dur. C | .908^***^ | .891 | .922 | .844^***^ | .814 | .869 | .918^***^ | .900 | .932 | .842^***^ | .783 | .881 |
| Dur. C’ | .882^***^ | .835 | .913 | .725^***^ | .663 | .774 | .884^***^ | .853 | .907 | .817^***^ | .771 | .853 |
| Dur. D | .887^***^ | .862 | .907 | .739^***^ | .691 | .780 | .888^***^ | .860 | .910 | .849^***^ | .815 | .877 |
| Occ. A | .909^***^ | .837 | .943 | .604^***^ | .491 | .687 | .933^***^ | .919 | .944 | .721^***^ | .668 | .765 |
| Occ. B | .879^***^ | .842 | .906 | .528^***^ | .436 | .604 | .928^***^ | .914 | .939 | .751^***^ | .704 | .791 |
| Occ. C | .837^***^ | .789 | .872 | .746^***^ | .649 | .810 | .873^***^ | .850 | .893 | .762^***^ | .717 | .799 |
| Occ. C’ | .927^***^ | .914 | .939 | .577^***^ | .499 | .642 | .927^***^ | .905 | .943 | .712^***^ | .656 | .758 |
| Occ. D | .921^***^ | .904 | .935 | .589^***^ | .512 | .653 | .926^***^ | .912 | .938 | .686^***^ | .617 | .741 |
| Cov. A | .893^***^ | .812 | .932 | .255^***^ | .113 | .373 | .927^***^ | .913 | .938 | -.040 | -.231 | .122 |
| Cov. B | .816^***^ | .616 | .894 | .142^*^ | -.013 | .273 | .915^***^ | .899 | .928 | .197^**^ | .049 | .322 |
| Cov. C | .874^***^ | .844 | .897 | .413^***^ | .304 | .506 | .912^***^ | .895 | .925 | .186^**^ | .038 | .312 |
| Cov. C’ | .915^***^ | .898 | .929 | .183^**^ | .034 | .310 | .922^***^ | .908 | .934 | .060 | -.113 | .206 |
| Cov. D | .915^***^ | .900 | .928 | .270^***^ | .136 | .384 | .919^***^ | .904 | .931 | .111^†^ | -.052 | .248 |
| Exp. Var. | .891^***^ | .863 | .912 | .890^***^ | .864 | .910 | .901^***^ | .878 | .920 | .880^***^ | .852 | .902 |
| Mean Dur. | .932^***^ | .914 | .945 | .919^***^ | .889 | .940 | .930^***^ | .904 | .947 | .927^***^ | .886 | .950 |
| Mean Occ. | .935^***^ | .912 | .950 | .932^***^ | .901 | .950 | .934^***^ | .903 | .953 | .934^***^ | .896 | .955 |
| Mean GFP | .968^***^ | .961 | .974 | .967^***^ | .961 | .973 | .969^***^ | .962 | .974 | .968^***^ | .962 | .973 |
| A to B | .360^***^ | .242 | .459 | .257^***^ | .120 | .372 | .407^***^ | .298 | .499 | .161^*^ | .006 | .291 |
| A to C | .353^***^ | .234 | .453 | .113^†^ | -.050 | .251 | .408^***^ | .299 | .499 | .066 | -.106 | .210 |
| A to C’ | .288^***^ | .157 | .398 | -.008 | -.193 | .149 | .407^***^ | .298 | .499 | .081 | -.087 | .223 |
| A to D | .484^***^ | .389 | .564 | .203^**^ | .057 | .327 | .357^***^ | .239 | .457 | .287^***^ | .155 | .398 |
| B to A | .336^***^ | .213 | .439 | .184^**^ | .035 | .310 | .459^***^ | .360 | .543 | .218^**^ | .075 | .338 |
| B to C | .400^***^ | .290 | .493 | .186^**^ | .036 | .312 | .361^***^ | .244 | .461 | .232^**^ | .091 | .352 |
| B to C’ | .261^***^ | .127 | .375 | .076 | -.092 | .219 | .406^***^ | .297 | .499 | .144^*^ | -.013 | .277 |
| B to D | .421^***^ | .315 | .511 | .081 | -.087 | .223 | .347^***^ | .228 | .449 | .162^*^ | .008 | .292 |
| C to A | .438^***^ | .333 | .526 | -.001 | -.185 | .154 | .490^***^ | .397 | .569 | .101 | -.064 | .241 |
| C to B | .409^***^ | .301 | .501 | .293^***^ | .164 | .402 | .415^***^ | .308 | .506 | .065 | -.106 | .210 |
| C to C’ | .439^***^ | .337 | .526 | .281^***^ | .148 | .392 | .434^***^ | .330 | .522 | .209^**^ | .063 | .332 |
| C to D | .462^***^ | .361 | .547 | .223^**^ | .081 | .342 | .473^***^ | .377 | .555 | .084 | -.083 | .226 |
| C’ to A | .235^***^ | .096 | .353 | .105^†^ | -.059 | .244 | .286^***^ | .156 | .397 | .145^*^ | -.012 | .278 |
| C’ to B | .297^***^ | .168 | .405 | .127^†^ | -.034 | .263 | .294^***^ | .164 | .404 | .103 | -.062 | .242 |
| C’ to C | .511^***^ | .422 | .587 | .287^**^ | .157 | .397 | .451^***^ | .350 | .536 | .232^**^ | .092 | .351 |
| C’ to D | .504^***^ | .412 | .580 | .335^***^ | .213 | .438 | .513^***^ | .423 | .588 | .269^***^ | .136 | .382 |
| D to A | .343^***^ | .222 | .445 | .131^*^ | -.027 | .266 | .481^***^ | .385 | .561 | .119^†^ | -.044 | .256 |
| D to B | .428^***^ | .323 | .517 | .207^**^ | .063 | .329 | .352^***^ | .232 | .452 | .239^***^ | .099 | .357 |
| D to C | .444^***^ | .342 | .530 | .134^*^ | -.025 | .268 | .506^***^ | .415 | .582 | .108^†^ | -.055 | .246 |
| D to C’ | .509^***^ | .419 | .585 | .370^***^ | .254 | .468 | .538^***^ | .452 | .610 | .297^***^ | .168 | .406 |

*Note.* *n* = 542; ^***^ = *p* < .001, ^**^ = *p* < .010, ^*^ = *p* < .050, ^†^ = *p* < .10; k-means = k-means clustering, AAHC = atomize and agglomerate hierarchical clustering; GM fitting = grand-mean fitting, Ind fitting = individual fitting; ICC = Intraclass correlation coefficient (model = two-way, type = agreement, alpha = .05), LB = lower bound of 95% confidence interval, UB = upper bound of 95% confidence interval. Microstate characteristics: Dur. X = mean duration of microstate types, Occ. X = mean occurrences of microstate types, Cov. X = percentage coverages of microstate types, Exp. Var. = total explained variance by all microstate types, Mean Dur. = mean duration of all microstate types per second, Mean Occ. = mean occurrences of all microstate types per second, Mean GFP = mean global field power in standard deviations, X to X = transitions between microstate types. Shown are short-term retest-reliabilities of EEG microstate characteristics across two resting EEG measures on day two (ICCs of pre- and post-measures; average interval = 99 minutes). Red (.00 < *ICC* < .50) = poor reliability; yellow (.50 < *ICC* < .75) = moderate reliability, light green (.75 < *ICC* < .90) = good reliability, dark green (*ICC* > .90) = excellent reliability.

**Table S8**

*Long-term retest-reliability of microstate characteristics across 63 days (pre-measures)*

|  | **ICCs of day one pre-measures and day two pre-measures** | | | | | | | | | | | |
| --- | --- | --- | --- | --- | --- | --- | --- | --- | --- | --- | --- | --- |
|  | **k-means** | | | | | | **AAHC** | | | | | |
| **Parameter** | **GM fitting** | | | **Ind fitting** | | | **GM fitting** | | | **Ind fitting** | | |
|  | ICC | LB | UB | ICC | LB | UB | ICC | LB | UB | ICC | LB | UB |
| Dur. A | .829^***^ | .766 | .871 | .753^***^ | .694 | .799 | .850^***^ | .822 | .873 | .849^***^ | .820 | .872 |
| Dur. B | .847^***^ | .818 | .871 | .713^***^ | .657 | .759 | .851^***^ | .823 | .874 | .839^***^ | .808 | .864 |
| Dur. C | .826^***^ | .791 | .856 | .843^***^ | .807 | .872 | .839^***^ | .809 | .864 | .830^***^ | .798 | .857 |
| Dur. C’ | .770^***^ | .719 | .810 | .723^***^ | .670 | .767 | .814^***^ | .779 | .843 | .822^***^ | .788 | .850 |
| Dur. D | .848^***^ | .789 | .887 | .190^**^ | .041 | .317 | .894^***^ | .874 | .911 | .835^***^ | .804 | .861 |
| Occ. A | .860^***^ | .768 | .907 | .582^***^ | .452 | .675 | .877^***^ | .854 | .897 | .703^***^ | .647 | .749 |
| Occ. B | .834^***^ | .803 | .860 | .597^***^ | .521 | .661 | .857^***^ | .830 | .879 | .718^***^ | .665 | .762 |
| Occ. C | .790^***^ | .672 | .856 | .674^***^ | .567 | .749 | .833^***^ | .802 | .859 | .752^***^ | .706 | .791 |
| Occ. C’ | .673^***^ | .610 | .725 | .656^***^ | .588 | .712 | .719^***^ | .666 | .763 | .723^***^ | .671 | .766 |
| Occ. D | .838^***^ | .782 | .876 | .520^***^ | .408 | .608 | .904^***^ | .886 | .919 | .655^***^ | .590 | .709 |
| Cov. A | .802^***^ | .661 | .872 | .178^*^ | .019 | .311 | .826^***^ | .794 | .853 | -.035 | -.229 | .128 |
| Cov. B | .744^***^ | .696 | .784 | .228^**^ | .085 | .348 | .770^***^ | .727 | .806 | -.050 | -.247 | .116 |
| Cov. C | .836^***^ | .746 | .887 | .264^***^ | .075 | .409 | .853^***^ | .826 | .876 | .024 | -.158 | .177 |
| Cov. C’ | .624^***^ | .542 | .690 | .320^***^ | .191 | .428 | .725^***^ | .674 | .768 | .043 | -.135 | .193 |
| Cov. D | .794^***^ | .691 | .855 | .119^†^ | -.039 | .253 | .886^***^ | .864 | .904 | -.097 | -.303 | .076 |
| Exp. Var. | .782^***^ | .716 | .830 | .827^***^ | .724 | .883 | .814^***^ | .769 | .849 | .819^***^ | .730 | .871 |
| Mean Dur. | .922^***^ | .907 | .934 | .932^***^ | .919 | .942 | .931^***^ | .918 | .942 | .928^***^ | .915 | .940 |
| Mean Occ. | .936^***^ | .923 | .946 | .936^***^ | .925 | .947 | .934^***^ | .921 | .944 | .938^***^ | .926 | .947 |
| Mean GFP | .965^***^ | .959 | .971 | .964^***^ | .958 | .970 | .966^***^ | .959 | .971 | .817^***^ | .782 | .846 |
| A to B | .361^***^ | .242 | .462 | .227^**^ | .083 | .348 | .378^***^ | .262 | .476 | .317^***^ | .189 | .424 |
| A to C | .294^***^ | .161 | .406 | .154^*^ | -.004 | .287 | .321^***^ | .194 | .428 | .137^*^ | -.023 | .272 |
| A to C’ | .361^***^ | .242 | .461 | .109^†^ | -.057 | .249 | .267^***^ | .131 | .381 | .120^†^ | -.044 | .259 |
| A to D | .353^***^ | .232 | .455 | .028 | -.154 | .182 | .281^***^ | .146 | .394 | .005 | -.181 | .162 |
| B to A | .399^***^ | .286 | .494 | .306^***^ | .177 | .415 | .396^***^ | .282 | .491 | .291^***^ | .157 | .404 |
| B to C | .456^***^ | .354 | .541 | .122^†^ | -.042 | .260 | .316^***^ | .188 | .424 | .066 | -.109 | .214 |
| B to C’ | .229^**^ | .085 | .351 | .188^**^ | .036 | .316 | .375^***^ | .258 | .473 | .060 | -.116 | .207 |
| B to D | .391^***^ | .277 | .487 | .120^†^ | -.044 | .259 | .330^***^ | .205 | .435 | -.013 | -.202 | .146 |
| C to A | .184^*^ | .031 | .313 | .157^*^ | -.001 | .290 | .266^***^ | .130 | .381 | .058 | -.118 | .206 |
| C to B | .258^***^ | .119 | .375 | .080 | -.092 | .225 | .462^***^ | .361 | .547 | .015 | -.170 | .170 |
| C to C’ | .513^***^ | .422 | .589 | .242^***^ | .102 | .360 | .517^***^ | .427 | .593 | .260^***^ | .122 | .377 |
| C to D | .480^***^ | .371 | .569 | .279^***^ | .145 | .392 | .554^***^ | .470 | .624 | .097 | -.072 | .239 |
| C’ to A | .278^***^ | .144 | .391 | .036 | -.140 | .185 | .218^**^ | .074 | .340 | .079 | -.092 | .223 |
| C’ to B | .211^**^ | .065 | .334 | .148^*^ | -.011 | .281 | .270^***^ | .134 | .385 | .119^†^ | -.045 | .258 |
| C’ to C | .563^***^ | .479 | .634 | .313^***^ | .183 | .422 | .517^***^ | .426 | .593 | .281^***^ | .147 | .394 |
| C’ to D | .612^***^ | .539 | .673 | .279^***^ | .144 | .392 | .611^***^ | .538 | .672 | .365^***^ | .246 | .465 |
| D to A | .250^***^ | .110 | .368 | .095 | -.074 | .237 | .362^***^ | .243 | .463 | .107^†^ | -.061 | .248 |
| D to B | .090 | -.081 | .233 | .138^*^ | -.024 | .274 | .294^***^ | .163 | .405 | .080 | -.091 | .224 |
| D to C | .510^***^ | .418 | .587 | .351^***^ | .230 | .454 | .451^***^ | .349 | .537 | .200^**^ | .051 | .326 |
| D to C’ | .580^***^ | .496 | .649 | .282^***^ | .148 | .395 | .584^***^ | .506 | .649 | .430^***^ | .324 | .520 |

*Note.* *n* = 525; ^***^ = *p* < .001, ^**^ = *p* < .010, ^*^ = *p* < .050, ^†^ = *p* < .10; k-means = k-means clustering, AAHC = atomize and agglomerate hierarchical clustering; GM fitting = grand-mean fitting, Ind fitting = individual fitting; ICC = Intraclass correlation coefficient (model = two-way, type = agreement, alpha = .05), LB = lower bound of 95% confidence interval, UB = upper bound of 95% confidence interval. Microstate characteristics: Dur. X = mean duration of microstate types, Occ. X = mean occurrences of microstate types, Cov. X = percentage coverages of microstate types, Exp. Var. = total explained variance by all microstate types, Mean Dur. = mean duration of all microstate types per second, Mean Occ. = mean occurrences of all microstate types per second, Mean GFP = mean global field power in standard deviations, X to X = transitions between microstate types. Shown are long-term retest-reliabilities of EEG microstate characteristics the two pre-measures (ICCs of day one and day two; average interval = 63 days). Red (.00 < *ICC* < .50) = poor reliability; yellow (.50 < *ICC* < .75) = moderate reliability, light green (.75 < *ICC* < .90) = good reliability, dark green (*ICC* > .90) = excellent reliability.

**Table S9**

*Long-term retest-reliability of microstate characteristics across 63 days (post-measures)*

|  | **ICCs of day one post-measures and day two post-measures** | | | | | | | | | | | |
| --- | --- | --- | --- | --- | --- | --- | --- | --- | --- | --- | --- | --- |
|  | **k-means** | | | | | | **AAHC** | | | | | |
| **Parameter** | **GM fitting** | | | **Ind fitting** | | | **GM fitting** | | | **Ind fitting** | | |
|  | ICC | LB | UB | ICC | LB | UB | ICC | LB | UB | ICC | LB | UB |
| Dur. A | .805^***^ | .541 | .895 | .732^***^ | .580 | .816 | .847^***^ | .819 | .871 | .848^***^ | .820 | .872 |
| Dur. B | .753^***^ | .593 | .837 | .434^***^ | .324 | .526 | .842^***^ | .813 | .867 | .848^***^ | .819 | .872 |
| Dur. C | .840^***^ | .811 | .866 | .846^***^ | .816 | .870 | .846^***^ | .817 | .870 | .859^***^ | .833 | .881 |
| Dur. C’ | .811^***^ | .762 | .848 | .772^***^ | .726 | .810 | .838^***^ | .808 | .864 | .853^***^ | .826 | .877 |
| Dur. D | .830^***^ | .749 | .879 | .775^***^ | .691 | .831 | .862^***^ | .837 | .884 | .847^***^ | .818 | .871 |
| Occ. A | .806^**^ | .442 | .906 | .600^**^ | .242 | .759 | .865^***^ | .839 | .887 | .780^***^ | .739 | .815 |
| Occ. B | .743^***^ | .653 | .804 | .593^***^ | .423 | .701 | .852^***^ | .824 | .875 | .707^***^ | .652 | .753 |
| Occ. C | .665^***^ | .550 | .744 | .659^***^ | .579 | .721 | .794^***^ | .755 | .826 | .780^***^ | .739 | .814 |
| Occ. C’ | .678^***^ | .617 | .729 | .675^***^ | .608 | .730 | .742^***^ | .694 | .783 | .712^***^ | .658 | .757 |
| Occ. D | .803^***^ | .747 | .844 | .615^***^ | .499 | .699 | .865^***^ | .840 | .887 | .788^***^ | .748 | .822 |
| Cov. A | .690^*^ | .116 | .853 | .151^†^ | -.082 | .329 | .778^***^ | .736 | .813 | .092 | -.078 | .236 |
| Cov. B | .490^***^ | .257 | .634 | .100 | -.070 | .243 | .738^***^ | .689 | .779 | .039 | -.141 | .191 |
| Cov. C | .755^***^ | .693 | .803 | .285^***^ | .143 | .402 | .836^***^ | .805 | .862 | .174^*^ | .020 | .305 |
| Cov. C’ | .631^***^ | .551 | .695 | .333^***^ | .201 | .442 | .754^***^ | .708 | .793 | .122^†^ | -.042 | .260 |
| Cov. D | .741^***^ | .632 | .811 | .113^†^ | -.046 | .249 | .820^***^ | .786 | .848 | .071 | -.102 | .217 |
| Exp. Var. | .835^***^ | .804 | .861 | .879^***^ | .852 | .901 | .857^***^ | .831 | .880 | .874^***^ | .848 | .895 |
| Mean Dur. | .928^***^ | .915 | .940 | .936^***^ | .925 | .946 | .926^***^ | .912 | .937 | .942^***^ | .931 | .951 |
| Mean Occ. | .938^***^ | .927 | .948 | .942^***^ | .931 | .951 | .935^***^ | .923 | .945 | .942^***^ | .931 | .951 |
| Mean GFP | .961^***^ | .953 | .967 | .958^***^ | .950 | .964 | .961^***^ | .953 | .967 | .959^***^ | .951 | .965 |
| A to B | .292^***^ | .159 | .404 | .269^***^ | .133 | .384 | .274^***^ | .139 | .387 | .179^*^ | .027 | .307 |
| A to C | .247^***^ | .106 | .366 | -.150 | -.363 | .030 | .370^***^ | .252 | .470 | .129^†^ | -.034 | .267 |
| A to C’ | .127^†^ | -.036 | .265 | .134^†^ | -.029 | .270 | .209^**^ | .063 | .332 | .000 | -.182 | .155 |
| A to D | .308^***^ | .179 | .417 | .094 | -.076 | .236 | .315^***^ | .188 | .423 | .130^†^ | -.033 | .267 |
| B to A | .156^*^ | -.005 | .292 | .139^*^ | -.017 | .271 | .293^***^ | .161 | .404 | .195^**^ | .045 | .322 |
| B to C | .299^***^ | .167 | .409 | .135^*^ | -.026 | .271 | .402^***^ | .291 | .497 | .167^*^ | .012 | .298 |
| B to C’ | .111^†^ | -.054 | .251 | .104 | -.064 | .245 | .265^***^ | .128 | .381 | .193^**^ | .044 | .320 |
| B to D | .148^*^ | -.009 | .282 | .028 | -.153 | .180 | .234^**^ | .092 | .354 | .001 | -.185 | .159 |
| C to A | .220^**^ | .076 | .342 | .096 | -.073 | .239 | .221^**^ | .075 | .344 | .141^*^ | -.020 | .276 |
| C to B | .197^**^ | .047 | .324 | .030 | -.150 | .182 | .201^**^ | .051 | .326 | .078 | -.093 | .223 |
| C to C’ | .205^**^ | .056 | .330 | .261^***^ | .124 | .377 | .414^***^ | .304 | .506 | .239^***^ | .097 | .359 |
| C to D | .296^***^ | .163 | .408 | .189^**^ | .040 | .316 | .434^***^ | .329 | .523 | .188^**^ | .038 | .315 |
| C’ to A | .129^†^ | -.028 | .263 | .299^***^ | .169 | .409 | .313^***^ | .185 | .421 | -.010 | -.199 | .149 |
| C’ to B | .028 | -.152 | .181 | .000 | -.187 | .157 | .218^**^ | .072 | .341 | .138^*^ | -.023 | .274 |
| C’ to C | .544^***^ | .459 | .616 | .229^**^ | .085 | .351 | .453^***^ | .351 | .539 | .224^**^ | .079 | .346 |
| C’ to D | .539^***^ | .452 | .612 | .313^***^ | .185 | .421 | .533^***^ | .445 | .607 | .270^***^ | .134 | .385 |
| D to A | .150^*^ | -.009 | .284 | -.001 | -.185 | .155 | .184^*^ | .031 | .312 | .015 | -.169 | .170 |
| D to B | .380^***^ | .249 | .486 | .147^*^ | -.008 | .279 | .245^***^ | .106 | .363 | .071 | -.104 | .217 |
| D to C | .363^***^ | .244 | .463 | .235^**^ | .092 | .355 | .501^***^ | .408 | .579 | .195^**^ | .044 | .322 |
| D to C’ | .392^***^ | .278 | .487 | .218^**^ | .072 | .341 | .468^***^ | .369 | .552 | .234^**^ | .090 | .354 |

*Note.* *n* = 525; ^***^ = *p* < .001, ^**^ = *p* < .010, ^*^ = *p* < .050, ^†^ = *p* < .10; k-means = k-means clustering, AAHC = atomize and agglomerate hierarchical clustering; GM fitting = grand-mean fitting, Ind fitting = individual fitting; ICC = Intraclass correlation coefficient (model = two-way, type = agreement, alpha = .05), LB = lower bound of 95% confidence interval, UB = upper bound of 95% confidence interval. Microstate characteristics: Dur. X = mean duration of microstate types, Occ. X = mean occurrences of microstate types, Cov. X = percentage coverages of microstate types, Exp. Var. = total explained variance by all microstate types, Mean Dur. = mean duration of all microstate types per second, Mean Occ. = mean occurrences of all microstate types per second, Mean GFP = mean global field power in standard deviations, X to X = transitions between microstate types. Shown are long-term retest-reliabilities of EEG microstate characteristics across the two post-measures (ICCs of day one and day two; average interval = 63 days). Red (.00 < *ICC* < .50) = poor reliability; yellow (.50 < *ICC* < .75) = moderate reliability, light green (.75 < *ICC* < .90) = good reliability, dark green (*ICC* > .90) = excellent reliability.

**Table S10**

*Long-term retest-reliability of microstate characteristics across 1-7 days*

|  | **ICCs of day one and day two (GM fitting only)** | | | | | | | | | | | |
| --- | --- | --- | --- | --- | --- | --- | --- | --- | --- | --- | --- | --- |
|  | **Pre-measures** | | | | | | **Post-measures** | | | | | |
| **Parameter** | **k-means** | | | **AAHC** | | | **k-means** | | | **AAHC** | | |
|  | ICC | LB | UB | ICC | LB | UB | ICC | LB | UB | ICC | LB | UB |
| Dur. A | .843^***^ | .755 | .896 | .885^***^ | .840 | .917 | .826^***^ | .585 | .910 | .876^***^ | .827 | .911 |
| Dur. B | .865^***^ | .812 | .903 | .856^***^ | .799 | .896 | .728^***^ | .525 | .832 | .842^***^ | .781 | .887 |
| Dur. C | .857^***^ | .797 | .899 | .878^***^ | .831 | .913 | .878^***^ | .830 | .912 | .902^***^ | .864 | .930 |
| Dur. C’ | .853^***^ | .783 | .898 | .837^***^ | .774 | .883 | .858^***^ | .792 | .902 | .909^***^ | .873 | .934 |
| Dur. D | .875^***^ | .820 | .912 | .885^***^ | .839 | .918 | .846^***^ | .735 | .904 | .870^***^ | .819 | .906 |
| Occ. A | .856^***^ | .754 | .909 | .862^***^ | .808 | .901 | .817^**^ | .420 | .917 | .872^***^ | .821 | .908 |
| Occ. B | .827^***^ | .759 | .875 | .846^***^ | .786 | .890 | .762^***^ | .645 | .837 | .859^***^ | .804 | .899 |
| Occ. C | .813^***^ | .719 | .872 | .774^***^ | .684 | .838 | .667^***^ | .513 | .769 | .784^***^ | .700 | .845 |
| Occ. C’ | .691^***^ | .571 | .778 | .713^***^ | .600 | .794 | .719^***^ | .609 | .798 | .765^***^ | .673 | .831 |
| Occ. D | .889^***^ | .844 | .921 | .928^***^ | .899 | .948 | .842^***^ | .776 | .888 | .899^***^ | .859 | .927 |
| Cov. A | .789^***^ | .620 | .872 | .808^***^ | .733 | .862 | .710^*^ | .120 | .869 | .796^***^ | .716 | .853 |
| Cov. B | .708^***^ | .594 | .790 | .736^***^ | .633 | .810 | .421^**^ | .125 | .608 | .704^***^ | .588 | .787 |
| Cov. C | .806^***^ | .705 | .869 | .823^***^ | .754 | .873 | .734^***^ | .618 | .814 | .826^***^ | .758 | .875 |
| Cov. C’ | .682^***^ | .549 | .774 | .742^***^ | .641 | .814 | .716^***^ | .603 | .797 | .834^***^ | .769 | .881 |
| Cov. D | .828^***^ | .751 | .880 | .893^***^ | .848 | .924 | .764^***^ | .644 | .840 | .849^***^ | .791 | .892 |
| Exp. Var. | .842^***^ | .776 | .888 | .860^***^ | .806 | .900 | .875^***^ | .826 | .910 | .890^***^ | .847 | .921 |
| Mean Dur. | .941^***^ | .918 | .958 | .944^***^ | .923 | .960 | .938^***^ | .914 | .956 | .942^***^ | .920 | .958 |
| Mean Occ. | .941^***^ | .918 | .958 | .941^***^ | .918 | .958 | .950^***^ | .930 | .964 | .949^***^ | .929 | .963 |
| Mean GFP | .949^***^ | .929 | .964 | .950^***^ | .931 | .964 | .964^***^ | .949 | .975 | .965^***^ | .949 | .975 |
| A to B | .405^**^ | .171 | .573 | .387^**^ | .150 | .558 | .399^**^ | .167 | .566 | .291^*^ | .021 | .487 |
| A to C | .321^*^ | .054 | .512 | .187 | -.133 | .416 | .307^*^ | .037 | .502 | .003 | -.391 | .284 |
| A to C’ | .215^†^ | -.094 | .437 | .351^**^ | .099 | .533 | .099 | -.255 | .353 | .292^*^ | .025 | .488 |
| A to D | .405^**^ | .173 | .573 | .383^**^ | .140 | .557 | .537^***^ | .356 | .667 | .437^***^ | .217 | .595 |
| B to A | .494^***^ | .298 | .636 | .387^**^ | .150 | .558 | .253^*^ | -.040 | .464 | .407^***^ | .177 | .573 |
| B to C | .590^***^ | .430 | .705 | .331^**^ | .068 | .520 | .149 | -.184 | .388 | .358^**^ | .109 | .538 |
| B to C’ | .373^**^ | .128 | .549 | .452^***^ | .238 | .605 | .254^*^ | -.038 | .464 | .452^***^ | .239 | .605 |
| B to D | .394^**^ | .160 | .564 | .387^**^ | .149 | .559 | .239^†^ | -.059 | .453 | .397^**^ | .164 | .565 |
| C to A | .454^***^ | .239 | .608 | .424^***^ | .200 | .586 | .216^†^ | -.073 | .430 | .425^***^ | .203 | .586 |
| C to B | .312^*^ | .041 | .506 | .528^***^ | .343 | .661 | .187 | -.126 | .414 | .287^*^ | .007 | .489 |
| C to C’ | .531^***^ | .346 | .663 | .639^***^ | .498 | .740 | .294^*^ | .016 | .493 | .420^***^ | .191 | .583 |
| C to D | .457^***^ | .247 | .608 | .701^***^ | .584 | .785 | .255^*^ | -.029 | .462 | .203^†^ | -.104 | .425 |
| C’ to A | .264^*^ | -.014 | .468 | .058 | -.309 | .322 | .253^*^ | -.027 | .459 | .376^**^ | .130 | .552 |
| C’ to B | .315^*^ | .055 | .505 | .203^†^ | -.102 | .425 | .134 | -.208 | .378 | .254^*^ | -.033 | .462 |
| C’ to C | .690^***^ | .567 | .778 | .560^***^ | .388 | .684 | .656^***^ | .521 | .752 | .571^***^ | .403 | .691 |
| C’ to D | .573^***^ | .407 | .693 | .666^***^ | .534 | .761 | .592^***^ | .431 | .707 | .619^***^ | .471 | .726 |
| D to A | .383^***^ | .142 | .557 | .457^***^ | .244 | .610 | .222^†^ | -.078 | .439 | .106 | -.240 | .356 |
| D to B | .049 | -.326 | .318 | .111 | -.236 | .361 | .319^*^ | .055 | .510 | .180 | -.116 | .401 |
| D to C | .510^***^ | .317 | .648 | .298^*^ | .023 | .495 | .392^**^ | .152 | .563 | .560^***^ | .389 | .684 |
| D to C’ | .508^***^ | .317 | .645 | .496^***^ | .298 | .638 | .419^***^ | .192 | .582 | .535^***^ | .353 | .666 |

*Note.* *n* = 143; ^***^ = *p* < .001, ^**^ = *p* < .01. ^*^ = *p* < .05. ^†^ = *p* < .10; k-means = k-means clustering, AAHC = atomize and agglomerate hierarchical clustering; ICC = Intraclass correlation coefficient (model = two-way, type = agreement, alpha = .05), LB = lower bound of 95% confidence interval, UB = upper bound of 95% confidence interval. Microstate characteristics: Dur. X = mean duration of microstate types, Occ. X = mean occurrences of microstate types, Cov. X = percentage coverages of microstate types, Exp. Var. = total explained variance by all microstate types, Mean Dur. = mean duration of all microstate types per second, Mean Occ. = mean occurrences of all microstate types per second, Mean GFP = mean global field power in standard deviations, X to X = transitions between microstate types. Shown are long-term retest-reliabilities of EEG microstate characteristics across pre- and post-measures (ICCs of day one and day two; interval = 1-7 days). Red (.00 < *ICC* < .50) = poor reliability; yellow (.50 < *ICC* < .75) = moderate reliability, light green (.75 < *ICC* < .90) = good reliability, dark green (*ICC* > .90) = excellent reliability.

**Table S11**

*Long-term retest-reliability of microstate characteristics across 8-30 days*

|  | **ICCs of day one and day two (GM fitting only)** | | | | | | | | | | | |
| --- | --- | --- | --- | --- | --- | --- | --- | --- | --- | --- | --- | --- |
|  | **Pre-measures** | | | | | | **Post-measures** | | | | | |
| **Parameter** | **k-means** | | | **AAHC** | | | **k-means** | | | **AAHC** | | |
|  | ICC | LB | UB | ICC | LB | UB | ICC | LB | UB | ICC | LB | UB |
| Dur. A | .766^***^ | .659 | .838 | .781^***^ | .690 | .845 | .724^***^ | .383 | .853 | .737^***^ | .627 | .814 |
| Dur. B | .776^***^ | .680 | .843 | .786^***^ | .697 | .849 | .737^***^ | .459 | .854 | .830^***^ | .759 | .880 |
| Dur. C | .821^***^ | .746 | .873 | .783^***^ | .693 | .847 | .835^***^ | .766 | .883 | .739^***^ | .631 | .816 |
| Dur. C’ | .590^***^ | .414 | .713 | .696^***^ | .570 | .785 | .694^***^ | .545 | .791 | .724^***^ | .609 | .805 |
| Dur. D | .780^***^ | .642 | .859 | .888^***^ | .841 | .921 | .797^***^ | .681 | .866 | .825^***^ | .753 | .877 |
| Occ. A | .873^***^ | .735 | .929 | .898^***^ | .855 | .928 | .800^**^ | .432 | .905 | .867^***^ | .811 | .906 |
| Occ. B | .829^***^ | .758 | .879 | .861^***^ | .804 | .902 | .792^***^ | .675 | .862 | .871^***^ | .817 | .909 |
| Occ. C | .769^***^ | .597 | .858 | .846^***^ | .782 | .891 | .590^***^ | .415 | .712 | .731^***^ | .619 | .810 |
| Occ. C’ | .569^***^ | .391 | .695 | .666^***^ | .527 | .765 | .469^***^ | .250 | .624 | .644^***^ | .495 | .749 |
| Occ. D | .715^***^ | .540 | .816 | .867^***^ | .812 | .906 | .726^***^ | .551 | .825 | .869^***^ | .814 | .907 |
| Cov. A | .841^***^ | .652 | .914 | .876^***^ | .824 | .912 | .720^*^ | .160 | .873 | .801^***^ | .718 | .860 |
| Cov. B | .750^***^ | .643 | .825 | .777^***^ | .685 | .843 | .622^**^ | .301 | .776 | .797^***^ | .712 | .856 |
| Cov. C | .876^***^ | .780 | .924 | .856^***^ | .796 | .898 | .801^***^ | .717 | .860 | .804^***^ | .723 | .862 |
| Cov. C’ | .484^***^ | .269 | .635 | .658^***^ | .516 | .758 | .409^**^ | .165 | .582 | .660^***^ | .519 | .760 |
| Cov. D | .692^***^ | .466 | .809 | .881^***^ | .832 | .916 | .719^***^ | .513 | .827 | .850^***^ | .787 | .894 |
| Exp. Var. | .848^***^ | .783 | .893 | .866^***^ | .810 | .905 | .848^***^ | .785 | .893 | .863^***^ | .806 | .903 |
| Mean Dur. | .895^***^ | .851 | .926 | .902^***^ | .862 | .931 | .906^***^ | .866 | .933 | .905^***^ | .865 | .933 |
| Mean Occ. | .909^***^ | .871 | .936 | .906^***^ | .868 | .934 | .898^***^ | .855 | .928 | .901^***^ | .859 | .930 |
| Mean GFP | .967^***^ | .953 | .977 | .968^***^ | .954 | .977 | .973^***^ | .962 | .981 | .973^***^ | .962 | .981 |
| A to B | .333^*^ | .055 | .529 | .328^*^ | .049 | .525 | .229^†^ | -.077 | .450 | .224^†^ | -.092 | .450 |
| A to C | .444^***^ | .215 | .607 | .485^***^ | .269 | .637 | .316^*^ | .029 | .518 | .384^**^ | .133 | .564 |
| A to C’ | .413^**^ | .173 | .584 | .239^†^ | -.074 | .461 | .040 | -.360 | .322 | .206^†^ | -.108 | .433 |
| A to D | .304^*^ | .014 | .508 | .264^*^ | -.044 | .481 | .276^*^ | -.022 | .487 | .441^***^ | .207 | .606 |
| B to A | .557^***^ | .374 | .687 | .492^***^ | .281 | .641 | .018 | -.330 | .284 | .311^*^ | .033 | .511 |
| B to C | .408^**^ | .163 | .581 | .264^*^ | -.044 | .481 | .295^*^ | .000 | .503 | .518^***^ | .318 | .660 |
| B to C’ | .176 | -.167 | .418 | .253^*^ | -.053 | .471 | -.002 | -.421 | .293 | .339^*^ | .064 | .533 |
| B to D | .352^**^ | .083 | .542 | .384^**^ | .129 | .564 | .077 | -.311 | .349 | .328^*^ | .048 | .525 |
| C to A | .055 | -.340 | .334 | .199 | -.137 | .435 | .246^†^ | -.069 | .468 | .086 | -.289 | .353 |
| C to B | .365^**^ | .099 | .552 | .405^**^ | .156 | .580 | .255^*^ | -.056 | .474 | .259^*^ | -.052 | .477 |
| C to C’ | .566^***^ | .386 | .693 | .382^**^ | .125 | .564 | .009 | -.398 | .298 | .494^***^ | .284 | .642 |
| C to D | .255^*^ | -.040 | .469 | .381^**^ | .126 | .562 | .102 | -.241 | .354 | .539^***^ | .347 | .674 |
| C’ to A | .093 | -.276 | .357 | .280^***^ | -.010 | .488 | .021 | -.337 | .291 | .047 | -.351 | .328 |
| C’ to B | .203^†^ | -.122 | .434 | .286^*^ | -.013 | .497 | -.229 | -.738 | .131 | .168 | -.180 | .413 |
| C’ to C | .601^***^ | .433 | .719 | .589^***^ | .418 | .710 | .612^***^ | .452 | .726 | .443^***^ | .209 | .607 |
| C’ to D | .695^***^ | .568 | .785 | .632^***^ | .478 | .740 | .604^***^ | .439 | .720 | .578^***^ | .402 | .702 |
| D to A | .057 | -.335 | .335 | .302^*^ | .016 | .506 | .145 | -.210 | .396 | .162 | -.190 | .409 |
| D to B | .189 | -.134 | .422 | .481^***^ | .268 | .633 | .492^***^ | .265 | .646 | .390^**^ | .138 | .568 |
| D to C | .447^***^ | .218 | .609 | .487^***^ | .275 | .638 | .309^*^ | .019 | .513 | .380^**^ | .120 | .562 |
| D to C’ | .647^***^ | .501 | .750 | .626^***^ | .471 | .736 | .581^***^ | .406 | .704 | .600^***^ | .434 | .717 |

*Note.* *n* = 129; ^***^ = *p* < .001, ^**^ = *p* < .01. ^*^ = *p* < .05. ^†^ = *p* < .10; k-means = k-means clustering, AAHC = atomize and agglomerate hierarchical clustering; ICC = Intraclass correlation coefficient (model = two-way, type = agreement, alpha = .05), LB = lower bound of 95% confidence interval, UB = upper bound of 95% confidence interval. Microstate characteristics: Dur. X = mean duration of microstate types, Occ. X = mean occurrences of microstate types, Cov. X = percentage coverages of microstate types, Exp. Var. = total explained variance by all microstate types, Mean Dur. = mean duration of all microstate types per second, Mean Occ. = mean occurrences of all microstate types per second, Mean GFP = mean global field power in standard deviations, X to X = transitions between microstate types. Shown are long-term retest-reliabilities of EEG microstate characteristics across pre- and post-measures (ICCs of day one and day two; interval = 8-30 days). Red (.00 < *ICC* < .50) = poor reliability; yellow (.50 < *ICC* < .75) = moderate reliability, light green (.75 < *ICC* < .90) = good reliability, dark green (*ICC* > .90) = excellent reliability.

**Table S12**

*Long-term retest-reliability of microstate characteristics across 1-3 months*

|  | **ICCs of day one and day two (GM fitting only)** | | | | | | | | | | | |
| --- | --- | --- | --- | --- | --- | --- | --- | --- | --- | --- | --- | --- |
|  | **Pre-measures** | | | | | | **Post-measures** | | | | | |
| **Parameter** | **k-means** | | | **AAHC** | | | **k-means** | | | **AAHC** | | |
|  | ICC | LB | UB | ICC | LB | UB | ICC | LB | UB | ICC | LB | UB |
| Dur. A | .825^***^ | .742 | .879 | .841^***^ | .778 | .886 | .822^***^ | .509 | .914 | .856^***^ | .800 | .897 |
| Dur. B | .860^***^ | .804 | .900 | .871^***^ | .820 | .907 | .752^***^ | .592 | .841 | .856^***^ | .799 | .897 |
| Dur. C | .757^***^ | .661 | .825 | .842^***^ | .779 | .886 | .833^***^ | .767 | .880 | .885^***^ | .840 | .918 |
| Dur. C’ | .756^***^ | .661 | .825 | .850^***^ | .791 | .892 | .807^***^ | .725 | .864 | .840^***^ | .777 | .885 |
| Dur. D | .830^***^ | .715 | .892 | .883^***^ | .837 | .916 | .815^***^ | .704 | .879 | .883^***^ | .836 | .916 |
| Occ. A | .857^***^ | .752 | .911 | .884^***^ | .838 | .917 | .791^**^ | .368 | .903 | .847^***^ | .787 | .891 |
| Occ. B | .843^***^ | .782 | .887 | .858^***^ | .802 | .898 | .745^***^ | .602 | .831 | .860^***^ | .806 | .900 |
| Occ. C | .766^***^ | .594 | .855 | .852^***^ | .794 | .894 | .694^***^ | .531 | .795 | .843^***^ | .781 | .887 |
| Occ. C’ | .694^***^ | .575 | .780 | .746^***^ | .647 | .818 | .739^***^ | .636 | .813 | .788^***^ | .705 | .848 |
| Occ. D | .807^***^ | .713 | .867 | .879^***^ | .831 | .913 | .775^***^ | .670 | .844 | .834^***^ | .769 | .881 |
| Cov. A | .792^***^ | .645 | .869 | .827^***^ | .759 | .876 | .668^*^ | .028 | .849 | .745^***^ | .646 | .817 |
| Cov. B | .759^***^ | .664 | .827 | .792^***^ | .710 | .851 | .483^**^ | .195 | .657 | .756^***^ | .660 | .825 |
| Cov. C | .803^***^ | .685 | .871 | .846^***^ | .785 | .889 | .750^***^ | .637 | .826 | .873^***^ | .823 | .909 |
| Cov. C’ | .650^***^ | .512 | .749 | .767^***^ | .675 | .833 | .687^***^ | .557 | .778 | .802^***^ | .724 | .858 |
| Cov. D | .790^***^ | .626 | .872 | .871^***^ | .821 | .908 | .725^***^ | .548 | .824 | .803^***^ | .725 | .858 |
| Exp. Var. | .718^***^ | .578 | .808 | .759^***^ | .650 | .832 | .831^***^ | .762 | .879 | .860^***^ | .805 | .899 |
| Mean Dur. | .892^***^ | .850 | .923 | .922^***^ | .891 | .944 | .939^***^ | .915 | .956 | .930^***^ | .903 | .950 |
| Mean Occ. | .932^***^ | .906 | .951 | .926^***^ | .897 | .947 | .942^***^ | .920 | .959 | .938^***^ | .914 | .956 |
| Mean GFP | .963^***^ | .948 | .973 | .964^***^ | .949 | .974 | .958^***^ | .942 | .970 | .958^***^ | .941 | .970 |
| A to B | .241^†^ | -.059 | .456 | .446^***^ | .228 | .602 | .332^**^ | .076 | .518 | .437^***^ | .219 | .595 |
| A to C | .108 | -.210 | .347 | .055 | -.318 | .322 | .207^†^ | -.087 | .424 | .476^***^ | .271 | .624 |
| A to C’ | .376^**^ | .136 | .551 | .195^†^ | -.118 | .421 | .049 | -.328 | .318 | .200^†^ | -.103 | .422 |
| A to D | .408^**^ | .175 | .576 | .146 | -.192 | .388 | .065 | -.304 | .329 | .021 | -.351 | .293 |
| B to A | .241^†^ | -.057 | .455 | .345^**^ | .089 | .530 | .220^†^ | -.069 | .433 | .182 | -.125 | .407 |
| B to C | .368^**^ | .119 | .547 | .421^***^ | .192 | .585 | .290^*^ | .014 | .489 | .292^*^ | .014 | .491 |
| B to C’ | .155 | -.180 | .395 | .404^**^ | .168 | .572 | -.126 | -.555 | .186 | .163 | -.161 | .397 |
| B to D | .415^***^ | .189 | .579 | .203^†^ | -.112 | .428 | .155 | -.165 | .389 | .020 | -.364 | .296 |
| C to A | .068 | -.294 | .330 | .155 | -.175 | .392 | .269^*^ | -.009 | .472 | .114 | -.235 | .365 |
| C to B | .237^†^ | -.063 | .453 | .419^***^ | .192 | .583 | .220^†^ | -.084 | .439 | .205^†^ | -.100 | .426 |
| C to C’ | .522^***^ | .336 | .657 | .553^***^ | .376 | .679 | .262^*^ | -.030 | .470 | .349^**^ | .093 | .532 |
| C to D | .645^***^ | .494 | .750 | .528^***^ | .344 | .661 | .383^**^ | .143 | .556 | .495^***^ | .296 | .638 |
| C’ to A | .331^**^ | .068 | .520 | .314^*^ | .052 | .504 | .029 | -.334 | .296 | .432^***^ | .208 | .593 |
| C’ to B | .079 | -.263 | .331 | .274^*^ | -.010 | .479 | .131 | -.205 | .374 | .270^*^ | -.018 | .476 |
| C’ to C | .472^***^ | .266 | .621 | .422^***^ | .197 | .585 | .478^***^ | .274 | .625 | .502^***^ | .306 | .643 |
| C’ to D | .552^***^ | .377 | .678 | .592^***^ | .432 | .707 | .521^***^ | .334 | .656 | .549^***^ | .373 | .676 |
| D to A | .151 | -.185 | .391 | .219^†^ | -.088 | .439 | .162 | -.169 | .400 | .182 | -.141 | .414 |
| D to B | .021 | -.368 | .298 | .355^**^ | .101 | .537 | .275^*^ | .001 | .475 | .032 | -.336 | .301 |
| D to C | .512^***^ | .319 | .650 | .431^***^ | .210 | .591 | .415^***^ | .184 | .580 | .501^***^ | .306 | .642 |
| D to C’ | .568^***^ | .400 | .689 | .604^***^ | .449 | .716 | .398^**^ | .160 | .568 | .270^*^ | -.011 | .474 |

*Note.* *n* = 142; ^***^ = *p* < .001, ^**^ = *p* < .01. ^*^ = *p* < .05. ^†^ = *p* < .10; k-means = k-means clustering, AAHC = atomize and agglomerate hierarchical clustering; ICC = Intraclass correlation coefficient (model = two-way, type = agreement, alpha = .05), LB = lower bound of 95% confidence interval, UB = upper bound of 95% confidence interval. Microstate characteristics: Dur. X = mean duration of microstate types, Occ. X = mean occurrences of microstate types, Cov. X = percentage coverages of microstate types, Exp. Var. = total explained variance by all microstate types, Mean Dur. = mean duration of all microstate types per second, Mean Occ. = mean occurrences of all microstate types per second, Mean GFP = mean global field power in standard deviations, X to X = transitions between microstate types. Shown are long-term retest-reliabilities of EEG microstate characteristics across pre- and post-measures (ICCs of day one and day two; interval = 31-90 days). Red (.00 < *ICC* < .50) = poor reliability; yellow (.50 < *ICC* < .75) = moderate reliability, light green (.75 < *ICC* < .90) = good reliability, dark green (*ICC* > .90) = excellent reliability.

**Table S13**

*Long-term retest-reliability of microstate characteristics across 3-6 months*

|  | **ICCs of day one and day two (GM fitting only)** | | | | | | | | | | | |
| --- | --- | --- | --- | --- | --- | --- | --- | --- | --- | --- | --- | --- |
|  | **Pre-measures** | | | | | | **Post-measures** | | | | | |
| **Parameter** | **k-means** | | | **AAHC** | | | **k-means** | | | **AAHC** | | |
|  | ICC | LB | UB | ICC | LB | UB | ICC | LB | UB | ICC | LB | UB |
| Dur. A | .858^***^ | .745 | .918 | .837^***^ | .738 | .899 | .798^***^ | .457 | .905 | .863^***^ | .779 | .915 |
| Dur. B | .872^***^ | .794 | .921 | .881^***^ | .810 | .926 | .778^***^ | .579 | .875 | .811^***^ | .695 | .883 |
| Dur. C | .858^***^ | .771 | .911 | .819^***^ | .710 | .888 | .762^***^ | .619 | .852 | .814^***^ | .699 | .885 |
| Dur. C’ | .807^***^ | .690 | .880 | .854^***^ | .765 | .909 | .845^***^ | .750 | .903 | .814^***^ | .701 | .884 |
| Dur. D | .890^***^ | .820 | .932 | .919^***^ | .870 | .950 | .850^***^ | .738 | .911 | .858^***^ | .772 | .912 |
| Occ. A | .845^***^ | .726 | .909 | .844^***^ | .749 | .903 | .838^***^ | .517 | .927 | .883^***^ | .812 | .927 |
| Occ. B | .780^***^ | .645 | .863 | .808^***^ | .692 | .880 | .620^***^ | .379 | .766 | .782^***^ | .649 | .865 |
| Occ. C | .834^***^ | .698 | .904 | .896^***^ | .833 | .936 | .681^***^ | .414 | .817 | .834^***^ | .733 | .897 |
| Occ. C’ | .710^***^ | .535 | .819 | .750^***^ | .598 | .844 | .674^***^ | .474 | .798 | .678^***^ | .484 | .800 |
| Occ. D | .901^***^ | .831 | .941 | .929^***^ | .887 | .956 | .854^***^ | .766 | .910 | .837^***^ | .723 | .902 |
| Cov. A | .803^***^ | .630 | .888 | .798^***^ | .675 | .875 | .698^*^ | .112 | .866 | .794^***^ | .669 | .872 |
| Cov. B | .710^***^ | .532 | .820 | .704^***^ | .525 | .816 | .314^†^ | -.082 | .568 | .604^***^ | .361 | .755 |
| Cov. C | .855^***^ | .738 | .916 | .882^***^ | .810 | .926 | .695^***^ | .510 | .810 | .817^***^ | .707 | .886 |
| Cov. C’ | .668^***^ | .466 | .794 | .738^***^ | .578 | .837 | .646^***^ | .431 | .780 | .636^***^ | .417 | .773 |
| Cov. D | .871^***^ | .771 | .925 | .902^***^ | .842 | .939 | .783^***^ | .651 | .865 | .767^***^ | .615 | .858 |
| Exp. Var. | .805^***^ | .650 | .886 | .836^***^ | .723 | .901 | .642^***^ | .423 | .778 | .697^***^ | .511 | .812 |
| Mean Dur. | .947^***^ | .915 | .967 | .939^***^ | .902 | .962 | .893^***^ | .823 | .934 | .888^***^ | .818 | .930 |
| Mean Occ. | .945^***^ | .912 | .966 | .948^***^ | .917 | .968 | .941^***^ | .905 | .963 | .930^***^ | .887 | .956 |
| Mean GFP | .981^***^ | .969 | .988 | .982^***^ | .970 | .989 | .948^***^ | .917 | .968 | .947^***^ | .916 | .967 |
| A to B | .454^**^ | .119 | .662 | .241 | -.218 | .528 | .206 | -.287 | .508 | .159 | -.361 | .479 |
| A to C | .405^*^ | .040 | .631 | .649^***^ | .438 | .782 | -.088 | -.724 | .317 | .434^*^ | .086 | .649 |
| A to C’ | .377^*^ | .001 | .611 | .330^†^ | -.082 | .585 | .283^†^ | -.149 | .554 | .132 | -.406 | .463 |
| A to D | .327^†^ | -.090 | .583 | .397^*^ | .029 | .626 | .258 | -.201 | .541 | .325^†^ | -.089 | .582 |
| B to A | .268^†^ | -.168 | .543 | .294^†^ | -.110 | .554 | .161 | -.317 | .471 | .151 | -.360 | .471 |
| B to C | .335^*^ | -.069 | .586 | .209 | -.277 | .510 | .475^**^ | .152 | .675 | .470^**^ | .150 | .670 |
| B to C’ | .281^†^ | -.162 | .554 | .447^**^ | .108 | .657 | .247 | -.215 | .533 | -.165 | -.889 | .279 |
| B to D | .360^*^ | -.023 | .600 | .186 | -.311 | .494 | .024 | -.564 | .392 | .147 | -.377 | .471 |
| C to A | .047 | -.543 | .410 | .348^*^ | -.034 | .591 | .284^†^ | -.157 | .556 | .156 | -.364 | .477 |
| C to B | .107 | -.444 | .447 | .462^**^ | .131 | .667 | .098 | -.453 | .440 | .178 | -.327 | .490 |
| C to C’ | .370^*^ | -.017 | .609 | .507^**^ | .203 | .694 | .221 | -.244 | .513 | .395^*^ | .030 | .624 |
| C to D | .452^**^ | .119 | .660 | .590^***^ | .340 | .745 | .302^†^ | -.127 | .567 | .431^*^ | .082 | .647 |
| C’ to A | .069 | -.472 | .415 | .072 | -.507 | .426 | .349^*^ | -.048 | .596 | .283^†^ | -.141 | .551 |
| C’ to B | .325^*^ | -.060 | .574 | .267 | -.183 | .546 | .008 | -.589 | .381 | .227 | -.240 | .519 |
| C’ to C | .377^*^ | .010 | .610 | .417^*^ | .058 | .639 | .422^*^ | .077 | .639 | .241 | -.229 | .531 |
| C’ to D | .575^***^ | .316 | .736 | .548^***^ | .276 | .718 | .370^*^ | -.011 | .608 | .338^*^ | -.063 | .588 |
| D to A | .454^**^ | .117 | .662 | .540^***^ | .256 | .715 | .175 | -.316 | .484 | .249 | -.216 | .535 |
| D to B | .067 | -.511 | .423 | .181 | -.301 | .487 | .442^**^ | .098 | .654 | .283^†^ | -.161 | .556 |
| D to C | .585^***^ | .331 | .743 | .652^***^ | .439 | .784 | .327^*^ | -.067 | .578 | .641^***^ | .425 | .776 |
| D to C’ | .601^***^ | .334 | .758 | .598^***^ | .356 | .749 | .178 | -.267 | .474 | .389^*^ | .022 | .619 |

*Note.* *n* = 70; ^***^ = *p* < .001, ^**^ = *p* < .01. ^*^ = *p* < .05. ^†^ = *p* < .10; k-means = k-means clustering, AAHC = atomize and agglomerate hierarchical clustering; ICC = Intraclass correlation coefficient (model = two-way, type = agreement, alpha = .05), LB = lower bound of 95% confidence interval, UB = upper bound of 95% confidence interval. Microstate characteristics: Dur. X = mean duration of microstate types, Occ. X = mean occurrences of microstate types, Cov. X = percentage coverages of microstate types, Exp. Var. = total explained variance by all microstate types, Mean Dur. = mean duration of all microstate types per second, Mean Occ. = mean occurrences of all microstate types per second, Mean GFP = mean global field power in standard deviations, X to X = transitions between microstate types. Shown are long-term retest-reliabilities of EEG microstate characteristics across pre- and post-measures (ICCs of day one and day two; interval = 91-180 days). Red (.00 < *ICC* < .50) = poor reliability; yellow (.50 < *ICC* < .75) = moderate reliability, light green (.75 < *ICC* < .90) = good reliability, dark green (*ICC* > .90) = excellent reliability.

**Table S14**

*Long-term retest-reliability of microstate characteristics across more than 6 months*

|  | **ICCs of day one and day two (GM fitting only)** | | | | | | | | | | | |
| --- | --- | --- | --- | --- | --- | --- | --- | --- | --- | --- | --- | --- |
|  | **Pre-measures** | | | | | | **Post-measures** | | | | | |
| **Parameter** | **k-means** | | | **AAHC** | | | **k-means** | | | **AAHC** | | |
|  | ICC | LB | UB | ICC | LB | UB | ICC | LB | UB | ICC | LB | UB |
| Dur. A | .797^***^ | .576 | .898 | .879^***^ | .772 | .935 | .769^***^ | .499 | .886 | .817^***^ | .658 | .903 |
| Dur. B | .839^***^ | .697 | .914 | .857^***^ | .733 | .924 | .803^***^ | .618 | .897 | .871^***^ | .757 | .931 |
| Dur. C | .944^***^ | .804 | .977 | .943^***^ | .890 | .970 | .917^***^ | .812 | .960 | .932^***^ | .872 | .964 |
| Dur. C’ | .773^***^ | .575 | .879 | .785^***^ | .597 | .886 | .766^***^ | .562 | .875 | .767^***^ | .561 | .876 |
| Dur. D | .834^***^ | .683 | .912 | .878^***^ | .771 | .935 | .825^***^ | .665 | .908 | .809^***^ | .640 | .898 |
| Occ. A | .844^***^ | .675 | .921 | .877^***^ | .771 | .934 | .761^**^ | .358 | .894 | .854^***^ | .727 | .922 |
| Occ. B | .899^***^ | .808 | .946 | .921^***^ | .850 | .959 | .684^***^ | .411 | .831 | .831^***^ | .682 | .910 |
| Occ. C | .706^**^ | .255 | .866 | .828^***^ | .679 | .908 | .701^***^ | .386 | .848 | .709^***^ | .452 | .845 |
| Occ. C’ | .705^***^ | .446 | .843 | .694^***^ | .424 | .837 | .782^***^ | .594 | .883 | .798^***^ | .620 | .892 |
| Occ. D | .909^***^ | .805 | .954 | .939^***^ | .887 | .968 | .876^***^ | .744 | .937 | .875^***^ | .766 | .933 |
| Cov. A | .752^***^ | .490 | .874 | .765^***^ | .558 | .875 | .576^*^ | -.030 | .806 | .702^***^ | .440 | .841 |
| Cov. B | .833^***^ | .688 | .911 | .854^***^ | .726 | .923 | .576^**^ | .216 | .772 | .761^***^ | .550 | .873 |
| Cov. C | .875^**^ | .393 | .956 | .919^***^ | .842 | .958 | .785^***^ | .510 | .896 | .864^***^ | .747 | .928 |
| Cov. C’ | .643^***^ | .337 | .809 | .678^***^ | .394 | .829 | .642^***^ | .337 | .808 | .645^***^ | .329 | .811 |
| Cov. D | .836^***^ | .656 | .918 | .893^***^ | .800 | .943 | .706^***^ | .429 | .846 | .714^***^ | .463 | .848 |
| Exp. Var. | .568^**^ | .182 | .771 | .647^***^ | .327 | .813 | .903^***^ | .820 | .948 | .917^***^ | .845 | .956 |
| Mean Dur. | .965^***^ | .933 | .981 | .960^***^ | .925 | .979 | .967^***^ | .939 | .982 | .946^***^ | .899 | .971 |
| Mean Occ. | .955^***^ | .915 | .976 | .956^***^ | .918 | .976 | .956^***^ | .918 | .976 | .937^***^ | .882 | .966 |
| Mean GFP | .979^***^ | .960 | .989 | .977^***^ | .956 | .988 | .951^***^ | .907 | .974 | .949^***^ | .905 | .973 |
| A to B | .450^*^ | -.003 | .702 | .517^*^ | .111 | .740 | -.034 | -.879 | .439 | -.034 | -.978 | .454 |
| A to C | .156 | -.358 | .507 | .030 | -.849 | .487 | .588^**^ | .224 | .781 | .651^***^ | .348 | .813 |
| A to C’ | .564^**^ | .175 | .769 | .200 | -.340 | .545 | .378^†^ | -.133 | .663 | .142 | -.537 | .531 |
| A to D | .197 | -.484 | .569 | .116 | -.665 | .530 | .352^†^ | -.183 | .650 | .242 | -.374 | .588 |
| B to A | .301 | -.226 | .613 | .472^*^ | .036 | .715 | -.155 | -1.05 | .365 | .230 | -.454 | .591 |
| B to C | .560^**^ | .188 | .763 | .245 | -.416 | .597 | .221 | -.473 | .586 | .408^*^ | -.077 | .679 |
| B to C’ | -.167 | -1.22 | .382 | .169 | -.566 | .558 | .370^†^ | -.191 | .666 | .438^*^ | -.023 | .695 |
| B to D | .540^**^ | .140 | .754 | .481^*^ | .028 | .723 | .189 | -.547 | .571 | -.239 | -1,351 | .343 |
| C to A | .083 | -.619 | .494 | .035 | -.771 | .479 | -.243 | -1.34 | .339 | .405 | -.077 | .677 |
| C to B | .066 | -.776 | .505 | .591^**^ | .233 | .782 | .183 | -.462 | .553 | -.498 | -1,903 | .215 |
| C to C’ | .511^*^ | .077 | .740 | .329 | -.276 | .645 | .357^†^ | -.223 | .659 | .292 | -.311 | .620 |
| C to D | .572^**^ | .209 | .770 | .502^*^ | .060 | .735 | .628^**^ | .300 | .802 | .589^**^ | .223 | .782 |
| C’ to A | .663^***^ | .375 | .820 | .331^†^ | -.217 | .637 | -.233 | -1.26 | .335 | .377^*^ | -.155 | .666 |
| C’ to B | -.074 | -1.06 | .434 | .386^†^ | -.164 | .675 | -.048 | -1.00 | .446 | -.013 | -.928 | .464 |
| C’ to C | .512^*^ | .075 | .741 | .523^*^ | .099 | .747 | -.408 | -1.72 | .260 | .153 | -.440 | .522 |
| C’ to D | .619^**^ | .284 | .797 | .493^*^ | .040 | .731 | .206 | -.429 | .567 | .141 | -.487 | .521 |
| D to A | .187 | -.545 | .569 | .419^*^ | -.093 | .691 | -.407 | -1.72 | .262 | .432^*^ | -.042 | .694 |
| D to B | .296 | -.311 | .623 | .177 | -.569 | .565 | .411^*^ | -.102 | .685 | .540^**^ | .129 | .756 |
| D to C | .508^*^ | .092 | .735 | .273 | -.365 | .612 | .171 | -.580 | .561 | .182 | -.558 | .568 |
| D to C’ | .620^**^ | .296 | .796 | .603^**^ | .249 | .789 | .055 | -.761 | .495 | .473^*^ | .011 | .719 |

*Note.* *n* = 41; ^***^ = *p* < .001, ^**^ = *p* < .01. ^*^ = *p* < .05. ^†^ = *p* < .10; k-means = k-means clustering, AAHC = atomize and agglomerate hierarchical clustering; ICC = Intraclass correlation coefficient (model = two-way, type = agreement, alpha = .05), LB = lower bound of 95% confidence interval, UB = upper bound of 95% confidence interval. Microstate characteristics: Dur. X = mean duration of microstate types, Occ. X = mean occurrences of microstate types, Cov. X = percentage coverages of microstate types, Exp. Var. = total explained variance by all microstate types, Mean Dur. = mean duration of all microstate types per second, Mean Occ. = mean occurrences of all microstate types per second, Mean GFP = mean global field power in standard deviations, X to X = transitions between microstate types. Shown are long-term retest-reliabilities of EEG microstate characteristics across pre- and post-measures (ICCs of day one and day two; interval of 181 days and more). Red (.00 < *ICC* < .50) = poor reliability; yellow (.50 < *ICC* < .75) = moderate reliability, light green (.75 < *ICC* < .90) = good reliability, dark green (*ICC* > .90) = excellent reliability.

**Table S15**

*Differences between average ICCs of groups with different intervals between day one and day two*

| **Pre/k-means** | | | | |
| --- | --- | --- | --- | --- |
| **Group comparisons** | **Durations** | **Occurrences** | **Coverages** | **Transitions** |
| 1 vs 2 | <.001 | .006 | .006 | .183 |
| 1 vs 3 | .004 | .117 | .117 | .054 |
| 1 vs 4 | .500 | .480 | .480 | .092 |
| 1 vs 5 | .337 | .355 | .359 | .215 |
| 2 vs 3 | .012 | .096 | .096 | .241 |
| 2 vs 4 | <.001 | .007 | .007 | .224 |
| 2 vs 5 | <.001 | .002 | .002 | .455 |
| 3 vs 4 | .004 | .127 | .127 | .392 |
| 3 vs 5 | .014 | .060 | .060 | .207 |
| 4 vs 5 | .337 | .340 | .340 | .294 |
| **Post/k-means** | | | | |
| 1 vs 2 | .001 | .004 | .371 | .068 |
| 1 vs 3 | .100 | .245 | .349 | .091 |
| 1 vs 4 | .126 | .234 | .154 | .088 |
| 1 vs 5 | .358 | .468 | .287 | .001 |
| 2 vs 3 | .029 | .027 | .477 | .439 |
| 2 vs 4 | .021 | .029 | .245 | .446 |
| 2 vs 5 | .002 | .003 | .408 | .058 |
| 3 vs 4 | .444 | .485 | .264 | .493 |
| 3 vs 5 | .178 | .221 | .431 | .042 |
| 4 vs 5 | .217 | .210 | .325 | .043 |
| **Pre/AAHC** | | | | |
| 1 vs 2 | <.001 | .478 | .233 | .364 |
| 1 vs 3 | .242 | .286 | .233 | .166 |
| 1 vs 4 | .448 | .149 | .299 | .469 |
| 1 vs 5 | .270 | .022 | .052 | .135 |
| 2 vs 3 | .001 | .305 | .500 | .266 |
| 2 vs 4 | <.001 | .162 | .421 | .393 |
| 2 vs 5 | <.001 | .025 | .185 | .226 |
| 3 vs 4 | .285 | .316 | .421 | .186 |
| 3 vs 5 | .095 | .074 | .185 | .448 |
| 4 vs 5 | .228 | .167 | .137 | .153 |
| **Post/AAHC** | | | | |
| 1 vs 2 | <.001 | .061 | .224 | .397 |
| 1 vs 3 | .123 | .351 | .392 | .087 |
| 1 vs 4 | .002 | .061 | .002 | .058 |
| 1 vs 5 | .022 | .124 | .008 | .025 |
| 2 vs 3 | <.001 | .123 | .314 | .135 |
| 2 vs 4 | .003 | .500 | .017 | .095 |
| 2 vs 5 | <.001 | .349 | .048 | .045 |
| 3 vs 4 | .036 | .123 | .004 | .416 |
| 3 vs 5 | .195 | .220 | .016 | .276 |
| 4 vs 5 | .172 | .349 | .320 | .351 |

*n* = 525. Groups: 1 = interval of 1-7 days, 2 = interval of 8-30 days, 3 = interval of 31-90 days, 4 = interval of 91-180 days, 5 = interval of 181 days and more. Pre = pre-measures, Post = post-measures, k-means = k-means clustering, AAHC = atomize and agglomerate hierarchical clustering. Shown are p-values of z-tests for the comparison of average ICCs of microstate durations, occurrences, coverages, and transitions across types between the five groups. Although there were significant differences between the retest-reliability of specific microstate characteristics between specific groups, there was no systematic decrease of the reliability with increasing intervals between day one and day two.

**Table S16**

*Methodological consistency of microstate characteristics across clustering procedures (day one)*

|  | **ICCs of day one k-means and Day one AAHC** | | | | | | | | | | | |
| --- | --- | --- | --- | --- | --- | --- | --- | --- | --- | --- | --- | --- |
|  | **Pre-measures** | | | | | | **Post-measures** | | | | | |
| **Parameter** | **GM fitting** | | | **Ind fitting** | | | **GM fitting** | | | **Ind fitting** | | |
|  | ICC | LB | UB | ICC | LB | UB | ICC | LB | UB | ICC | LB | UB |
| Dur. A | .976^***^ | .930 | .988 | .871^***^ | .848 | .891 | .981^***^ | .945 | .990 | .883^***^ | .863 | .901 |
| Dur. B | .970^***^ | .820 | .988 | .813^***^ | .779 | .841 | .972^***^ | .829 | .990 | .546^***^ | .466 | .614 |
| Dur. C | .905^***^ | .766 | .949 | .886^***^ | .815 | .923 | .915^***^ | .788 | .955 | .879^***^ | .793 | .922 |
| Dur. C’ | .820^***^ | .514 | .910 | .845^***^ | .756 | .895 | .839^***^ | .540 | .921 | .859^***^ | .773 | .905 |
| Dur. D | .935^***^ | .888 | .958 | .216^**^ | .080 | .333 | .938^***^ | .901 | .958 | .856^***^ | .752 | .907 |
| Occ. A | .983^***^ | .914 | .993 | .750^***^ | .702 | .789 | .984^***^ | .866 | .994 | .782^***^ | .741 | .816 |
| Occ. B | .974^**^ | .509 | .993 | .668^***^ | .588 | .731 | .974^**^ | .550 | .992 | .773^***^ | .707 | .821 |
| Occ. C | .641 | -.262 | .875 | .722^***^ | .509 | .825 | .675 | -.237 | .893 | .690^***^ | .530 | .783 |
| Occ. C’ | .849^**^ | .402 | .936 | .674^***^ | .388 | .802 | .870^**^ | .547 | .941 | .697^***^ | .423 | .818 |
| Occ. D | .920^***^ | .905 | .932 | .714^***^ | .635 | .772 | .911^***^ | .895 | .924 | .822^***^ | .761 | .863 |
| Cov. A | .969^***^ | .745 | .989 | .148^*^ | .000 | .274 | .971^**^ | .618 | .991 | .148^*^ | -.001 | .274 |
| Cov. B | .949^*^ | .116 | .986 | -.027 | -.196 | .120 | .948^*^ | .098 | .986 | .180^**^ | .035 | .304 |
| Cov. C | .796^*^ | -.142 | .932 | .216^*^ | -.041 | .399 | .807^*^ | -.158 | .938 | .176^†^ | -.046 | .344 |
| Cov. C’ | .743^*^ | .126 | .887 | .049 | -.089 | .174 | .765^**^ | .222 | .895 | .079 | -.078 | .214 |
| Cov. D | .882^***^ | .843 | .908 | .227^**^ | .041 | .372 | .875^***^ | .843 | .899 | .180^*^ | -.002 | .325 |
| Exp. Var. | .989^**^ | .671 | .997 | .990^**^ | .440 | .997 | .982^**^ | .517 | .995 | .984^*^ | .253 | .996 |
| Mean Dur. | .988^***^ | .984 | .990 | .980^***^ | .964 | .987 | .990^***^ | .988 | .992 | .987^***^ | .967 | .993 |
| Mean Occ. | .996^***^ | .995 | .996 | .991^***^ | .983 | .994 | .996^***^ | .996 | .997 | .994^***^ | .986 | .996 |
| Mean GFP | 1.00^***^ | 1.00 | 1.00 | 1.00^***^ | 1.00 | 1.00 | 1.00^***^ | .998 | 1.00 | .998^***^ | .997 | .999 |
| A to B | .862^***^ | .838 | .883 | .393^***^ | .286 | .484 | .880^***^ | .859 | .898 | .426^***^ | .324 | .512 |
| A to C | .696^***^ | .484 | .803 | .260^***^ | .130 | .370 | .728^***^ | .600 | .806 | .377^***^ | .267 | .471 |
| A to C’ | .641^***^ | .578 | .695 | .213^**^ | .074 | .331 | .568^***^ | .491 | .633 | .351^***^ | .237 | .449 |
| A to D | .737^***^ | .630 | .805 | .239^***^ | .105 | .353 | .723^***^ | .634 | .785 | .363^***^ | .251 | .459 |
| B to A | .878^***^ | .856 | .896 | .392^***^ | .285 | .483 | .872^***^ | .849 | .891 | .352^***^ | .238 | .449 |
| B to C | .695^***^ | .537 | .787 | .373^***^ | .263 | .467 | .709^***^ | .539 | .802 | .267^***^ | .138 | .376 |
| B to C’ | .577^***^ | .474 | .657 | .084^***^ | -.073 | .218 | .586^***^ | .507 | .651 | .241^***^ | .108 | .354 |
| B to D | .714^***^ | .664 | .757 | .246^***^ | .113 | .359 | .712^***^ | .660 | .756 | .421^***^ | .319 | .508 |
| C to A | .740^***^ | .694 | .779 | .317^***^ | .196 | .420 | .699^***^ | .645 | .744 | .313^***^ | .192 | .416 |
| C to B | .628^***^ | .562 | .684 | .258^***^ | .128 | .369 | .669^***^ | .611 | .719 | .211^**^ | .073 | .329 |
| C to C’ | .761^***^ | .707 | .804 | .386^***^ | .278 | .479 | .699^***^ | .646 | .744 | .321^***^ | .201 | .423 |
| C to D | .747^***^ | .640 | .815 | .359^***^ | .243 | .457 | .714^***^ | .654 | .763 | .414^***^ | .311 | .502 |
| C’ to A | .597^***^ | .524 | .658 | .302^***^ | .179 | .406 | .631^***^ | .565 | .686 | .312^***^ | .190 | .415 |
| C’ to B | .687^***^ | .631 | .734 | .194^**^ | .052 | .315 | .648^***^ | .585 | .701 | .205^**^ | .065 | .324 |
| C’ to C | .779^***^ | .709 | .828 | .370^***^ | .258 | .464 | .730^***^ | .603 | .807 | .339^***^ | .220 | .439 |
| C’ to D | .807^***^ | .770 | .838 | .433^***^ | .331 | .519 | .810^***^ | .774 | .840 | .525^***^ | .441 | .596 |
| D to A | .689^***^ | .466 | .800 | .204^**^ | .060 | .325 | .752^***^ | .592 | .836 | .248^***^ | .116 | .360 |
| D to B | .749^***^ | .677 | .802 | .196^**^ | .055 | .316 | .708^***^ | .628 | .767 | .290^***^ | .165 | .397 |
| D to C | .687^**^ | .336 | .823 | .393^***^ | .278 | .488 | .691^***^ | .471 | .801 | .401^***^ | .294 | .492 |
| D to C’ | .740^***^ | .603 | .818 | .495^***^ | .406 | .571 | .775^***^ | .682 | .835 | .470^***^ | .376 | .549 |

*Note.* *n* = 583; ^***^ = *p* < .001, ^**^ = *p* < .01. ^*^ = *p* < .05. ^†^ = *p* < .10; k-means = k-means clustering, AAHC = atomize and agglomerate hierarchical clustering; GM fitting = grand-mean fitting, Ind fitting = individual fitting; ICC = Intraclass correlation coefficient (model = two-way, type = agreement, alpha = .05), LB = lower bound of 95% confidence interval, UB = upper bound of 95% confidence interval. Microstate characteristics: Dur. X = mean duration of microstate types, Occ. X = mean occurrences of microstate types, Cov. X = percentage coverages of microstate types, Exp. Var. = total explained variance by all microstate types, Mean Dur. = mean duration of all microstate types per second, Mean Occ. = mean occurrences of all microstate types per second, Mean GFP = mean global field power in standard deviations, X to X = transitions between microstate types. Shown is the methodological consistency of EEG microstate characteristics across clustering procedures for pre- and post-measures of day one (ICCs of microstate characteristics obtained from k-means clustering and AAHC). Red (.00 < *ICC* < .50) = poor reliability; yellow (.50 < *ICC* < .75) = moderate reliability, light green (.75 < *ICC* < .90) = good reliability, dark green (*ICC* > .90) = excellent reliability.

**Table S17**

*Methodological consistency of microstate characteristics across clustering procedures (day two)*

|  | **ICCs of day two k-means and day two AAHC** | | | | | | | | | | | |
| --- | --- | --- | --- | --- | --- | --- | --- | --- | --- | --- | --- | --- |
|  | **Pre-measures** | | | | | | **Post-measures** | | | | | |
| **Parameter** | **GA fit** | | | **Ind fitting** | | | **GA fit** | | | **Ind fitting** | | |
|  | ICC | LB | UB | ICC | LB | UB | ICC | LB | UB | ICC | LB | UB |
| Dur. A | .919^**^ | .520 | .970 | .790^***^ | .710 | .843 | .840^*^ | .127 | .941 | .792^***^ | .561 | .882 |
| Dur. B | .983^***^ | .944 | .992 | .812^***^ | .777 | .841 | .927^***^ | .898 | .946 | .823^***^ | .738 | .874 |
| Dur. C | .871^*^ | .018 | .959 | .825^***^ | .373 | .923 | .869^**^ | .388 | .948 | .825^***^ | .617 | .902 |
| Dur. C’ | .943^***^ | .762 | .976 | .825^***^ | .787 | .856 | .939^***^ | .846 | .968 | .813^***^ | .778 | .843 |
| Dur. D | .992^***^ | .979 | .996 | .812^***^ | .777 | .841 | .984^***^ | .966 | .991 | .857^***^ | .831 | .879 |
| Occ. A | .955^*^ | .117 | .988 | .597^***^ | .371 | .725 | .889^*^ | -.033 | .967 | .609^*^ | .021 | .803 |
| Occ. B | .979^***^ | .792 | .993 | .649^***^ | .584 | .704 | .929^***^ | .916 | .940 | .680^***^ | .608 | .737 |
| Occ. C | .595 | -.116 | .872 | .647^***^ | -.083 | .843 | .559 | -.235 | .842 | .711^***^ | .250 | .854 |
| Occ. C’ | .958^*^ | .245 | .988 | .653^***^ | .512 | .744 | .967^***^ | .852 | .986 | .625^***^ | .516 | .704 |
| Occ. D | .991^***^ | .890 | .997 | .670^***^ | .610 | .721 | .985^***^ | .879 | .995 | .691^***^ | .634 | .739 |
| Cov. A | .920^*^ | -.055 | .979 | -.072 | -.255 | .086 | .790^†^ | -.191 | .934 | .038 | -.091 | .157 |
| Cov. B | .972^**^ | .536 | .992 | .040^***^ | -.137 | .189 | .878^***^ | .852 | .899 | .061 | -.094 | .196 |
| Cov. C | .727^†^ | -.071 | .927 | .050^***^ | -.107 | .190 | .675 | -.226 | .895 | .123 | -.138 | .321 |
| Cov. C’ | .940^*^ | .161 | .983 | .133^***^ | -.023 | .266 | .949^***^ | .690 | .981 | .018 | -.149 | .163 |
| Cov. D | .990^**^ | .686 | .997 | .188^***^ | .039 | .315 | .982^**^ | .755 | .994 | .140^*^ | -.018 | .274 |
| Exp. Var. | .979^**^ | .428 | .994 | .984^*^ | .154 | .996 | .972^**^ | .328 | .992 | .980^*^ | .106 | .995 |
| Mean Dur. | .985^***^ | .978 | .989 | .975^***^ | .933 | .987 | .987^***^ | .983 | .989 | .978^***^ | .939 | .989 |
| Mean Occ. | .994^***^ | .992 | .995 | .989^***^ | .962 | .995 | .994^***^ | .992 | .995 | .990^***^ | .969 | .995 |
| Mean GFP | .999^***^ | .992 | 1.00 | .999^***^ | .993 | .999 | .999^***^ | .995 | 1.00 | .999^***^ | .994 | .999 |
| A to B | .854^***^ | .828 | .877 | .401^***^ | .270 | .507 | .680^***^ | .621 | .730 | .409^***^ | .281 | .512 |
| A to C | .696^*^ | .139 | .854 | .248^***^ | .111 | .365 | .480^**^ | .105 | .669 | .136^*^ | -.019 | .267 |
| A to C’ | .732^***^ | .619 | .804 | .342^***^ | .220 | .444 | .502^***^ | .375 | .600 | .145^*^ | -.008 | .275 |
| A to D | .835^***^ | .775 | .876 | .271^***^ | .138 | .385 | .723^***^ | .671 | .766 | .267^***^ | .132 | .381 |
| B to A | .872^***^ | .849 | .892 | .391^***^ | .279 | .485 | .687^***^ | .622 | .739 | .384^***^ | .271 | .479 |
| B to C | .805^***^ | .558 | .893 | .264^***^ | .129 | .377 | .659^***^ | .558 | .732 | .286^***^ | .156 | .397 |
| B to C’ | .836^***^ | .764 | .881 | .436^***^ | .327 | .526 | .734^***^ | .685 | .775 | .340^***^ | .218 | .442 |
| B to D | .906^***^ | .839 | .940 | .257^***^ | .121 | .372 | .742^***^ | .616 | .818 | .371^***^ | .255 | .468 |
| C to A | .805^***^ | .769 | .835 | .205^***^ | .059 | .327 | .648^***^ | .581 | .703 | .138^***^ | -.020 | .272 |
| C to B | .866^***^ | .841 | .887 | .310^***^ | .183 | .417 | .618^***^ | .547 | .677 | .167^***^ | .013 | .296 |
| C to C’ | .865^***^ | .840 | .886 | .251^***^ | .113 | .367 | .680^***^ | .621 | .730 | .256^***^ | .119 | .371 |
| C to D | .859^***^ | .829 | .883 | .301^***^ | .172 | .409 | .751^***^ | .705 | .790 | .315^***^ | .189 | .422 |
| C’ to A | .767^***^ | .696 | .819 | .344^***^ | .219 | .449 | .501^***^ | .389 | .590 | .137^*^ | -.019 | .269 |
| C’ to B | .857^***^ | .815 | .887 | .371^***^ | .256 | .469 | .700^***^ | .644 | .747 | .172^*^ | .021 | .299 |
| C’ to C | .805^***^ | .527 | .897 | .440^***^ | .310 | .543 | .632^***^ | .406 | .754 | .277^***^ | .141 | .392 |
| C’ to D | .918^***^ | .898 | .933 | .508^***^ | .418 | .585 | .880^***^ | .854 | .901 | .335^***^ | .213 | .438 |
| D to A | .841^***^ | .725 | .898 | .180^***^ | .033 | .306 | .664^***^ | .538 | .748 | .178^*^ | .028 | .305 |
| D to B | .904^***^ | .844 | .936 | .290^***^ | .161 | .400 | .719^***^ | .667 | .763 | .352^***^ | .233 | .453 |
| D to C | .797^*^ | .192 | .916 | .332^***^ | .190 | .447 | .625^***^ | .380 | .753 | .132^*^ | -.024 | .265 |
| D to C’ | .909^***^ | .873 | .932 | .464^***^ | .365 | .547 | .861^***^ | .834 | .883 | .357^***^ | .238 | .457 |

*Note.* *n* = 542; ^***^ = *p* < .001, ^**^ = *p* < .01. ^*^ = *p* < .05. ^†^ = *p* < .10; k-means = k-means clustering, AAHC = atomize and agglomerate hierarchical clustering; GM fitting = grand-mean fitting, Ind fitting = individual fitting; ICC = Intraclass correlation coefficient (model = two-way, type = agreement, alpha = .05), LB = lower bound of 95% confidence interval, UB = upper bound of 95% confidence interval. Microstate characteristics: Dur. X = mean duration of microstate types, Occ. X = mean occurrences of microstate types, Cov. X = percentage coverages of microstate types, Exp. Var. = total explained variance by all microstate types, Mean Dur. = mean duration of all microstate types per second, Mean Occ. = mean occurrences of all microstate types per second, Mean GFP = mean global field power in standard deviations, X to X = transitions between microstate types. Shown is the methodological consistency of EEG microstate characteristics across clustering procedures for pre- and post-measures of day two (ICCs of microstate characteristics obtained from k-means clustering and AAHC). Red (.00 < *ICC* < .50) = poor reliability; yellow (.50 < *ICC* < .75) = moderate reliability, light green (.75 < *ICC* < .90) = good reliability, dark green (*ICC* > .90) = excellent reliability.

**Table S18**

*Methodological consistency of microstate characteristics across fitting procedures (day one)*

|  | **ICCs day one GM fitting and day one Ind fitting** | | | | | | | | | | | |
| --- | --- | --- | --- | --- | --- | --- | --- | --- | --- | --- | --- | --- |
|  | **Pre-measures** | | | | | | **Post-measures** | | | | | |
| **Parameter** | **k-means** | | | **AAHC** | | | **k-means** | | | **AAHC** | | |
|  | ICC | LB | UB | ICC | LB | UB | ICC | LB | UB | ICC | LB | UB |
| Dur. A | .818^***^ | .785 | .845 | .824^***^ | .793 | .850 | .832^***^ | .801 | .857 | .834^***^ | .804 | .859 |
| Dur. B | .788^***^ | .751 | .820 | .846^***^ | .819 | .869 | .495^***^ | .405 | .570 | .854^***^ | .828 | .876 |
| Dur. C | .744^***^ | .688 | .788 | .721^***^ | .672 | .763 | .775^***^ | .732 | .811 | .761^***^ | .718 | .796 |
| Dur. C’ | .832^***^ | .788 | .865 | .783^***^ | .744 | .815 | .833^***^ | .799 | .861 | .801^***^ | .765 | .831 |
| Dur. D | .277^***^ | .150 | .385 | .787^***^ | .750 | .819 | .828^***^ | .798 | .854 | .802^***^ | .767 | .832 |
| Occ. A | .777^***^ | .737 | .810 | .748^***^ | .704 | .786 | .809^***^ | .775 | .838 | .733^***^ | .684 | .774 |
| Occ. B | .801^***^ | .766 | .831 | .730^***^ | .682 | .771 | .815^***^ | .783 | .843 | .775^***^ | .735 | .809 |
| Occ. C | .510^***^ | .424 | .584 | -.063 | -.237 | .089 | .566^***^ | .490 | .631 | .053 | -.088 | .178 |
| Occ. C’ | .816^***^ | .783 | .844 | .585^***^ | .512 | .648 | .806^***^ | .772 | .835 | .605^***^ | .535 | .664 |
| Occ. D | .745^***^ | .700 | .783 | .701^***^ | .648 | .746 | .757^***^ | .714 | .794 | .739^***^ | .692 | .779 |
| Cov. A | .406^***^ | .301 | .495 | .171^*^ | .026 | .295 | .487^***^ | .397 | .564 | .065 | -.097 | .204 |
| Cov. B | .542^***^ | .461 | .611 | .150^*^ | .002 | .276 | .503^***^ | .415 | .577 | .222^**^ | .085 | .338 |
| Cov. C | .504^***^ | .416 | .579 | .026 | -.130 | .163 | .513^***^ | .427 | .586 | .064 | -.086 | .195 |
| Cov. C’ | .666^***^ | .606 | .717 | .118^†^ | -.037 | .250 | .589^***^ | .516 | .650 | .090 | -.070 | .225 |
| Cov. D | .506^***^ | .418 | .580 | .168^*^ | .023 | .293 | .423^***^ | .321 | .509 | .201^**^ | .062 | .320 |
| Exp. Var. | .856^**^ | .454 | .937 | .857^**^ | .410 | .940 | .852^*^ | .130 | .947 | .844^*^ | .116 | .944 |
| Mean Dur. | .965^***^ | .958 | .971 | .960^***^ | .943 | .971 | .973^***^ | .967 | .977 | .972^***^ | .951 | .982 |
| Mean Occ. | .988^***^ | .986 | .990 | .984^***^ | .973 | .990 | .990^***^ | .988 | .992 | .989^***^ | .976 | .994 |
| Mean GFP | .996^***^ | .996 | .997 | .864^***^ | .840 | .884 | .998^***^ | .998 | .998 | .999^***^ | .998 | .999 |
| A to B | .572^***^ | .454 | .660 | .499^***^ | .325 | .619 | .571^***^ | .466 | .652 | .453^***^ | .298 | .567 |
| A to C | .391^***^ | .284 | .483 | .171^*^ | .021 | .297 | .411^***^ | .307 | .499 | .270^***^ | .123 | .391 |
| A to C’ | .486^***^ | .395 | .563 | .359^***^ | .246 | .455 | .397^***^ | .290 | .487 | .286^***^ | .160 | .393 |
| A to D | .481^***^ | .389 | .558 | .317^***^ | .197 | .420 | .417^***^ | .313 | .504 | .271^***^ | .143 | .380 |
| B to A | .586^***^ | .502 | .655 | .495^***^ | .361 | .596 | .530^***^ | .424 | .614 | .479^***^ | .331 | .589 |
| B to C | .448^***^ | .350 | .531 | .168^*^ | .024 | .292 | .477^***^ | .385 | .556 | .225^**^ | .088 | .341 |
| B to C’ | .426^***^ | .325 | .512 | .233^***^ | .097 | .348 | .388^***^ | .280 | .480 | .366^***^ | .254 | .461 |
| B to D | .425^***^ | .323 | .511 | .366^***^ | .254 | .461 | .499^***^ | .411 | .575 | .405^***^ | .300 | .494 |
| C to A | .425^***^ | .324 | .512 | .342^***^ | .223 | .442 | .423^***^ | .322 | .510 | .390^***^ | .282 | .481 |
| C to B | .371^***^ | .260 | .466 | .262^***^ | .133 | .372 | .429^***^ | .328 | .514 | .252^***^ | .120 | .363 |
| C to C’ | .652^***^ | .591 | .704 | .388^***^ | .280 | .479 | .611^***^ | .542 | .669 | .417^***^ | .315 | .505 |
| C to D | .620^***^ | .545 | .682 | .547^***^ | .467 | .615 | .663^***^ | .602 | .715 | .435^***^ | .335 | .519 |
| C’ to A | .490^***^ | .400 | .567 | .363^***^ | .251 | .459 | .399^***^ | .293 | .490 | .354^***^ | .240 | .451 |
| C’ to B | .519^***^ | .434 | .591 | .358^***^ | .245 | .455 | .462^***^ | .367 | .542 | .323^***^ | .204 | .425 |
| C’ to C | .539^***^ | .458 | .609 | .436^***^ | .336 | .521 | .601^***^ | .531 | .661 | .531^***^ | .448 | .602 |
| C’ to D | .689^***^ | .634 | .736 | .536^***^ | .447 | .610 | .540^***^ | .459 | .609 | .653^***^ | .579 | .712 |
| D to A | .421^***^ | .319 | .508 | .378^***^ | .268 | .471 | .484^***^ | .393 | .562 | .426^***^ | .324 | .512 |
| D to B | .482^***^ | .390 | .559 | .367^***^ | .255 | .462 | .405^***^ | .300 | .494 | .455^***^ | .359 | .537 |
| D to C | .531^***^ | .441 | .605 | .498^***^ | .409 | .574 | .578^***^ | .501 | .642 | .379^***^ | .269 | .472 |
| D to C’ | .625^***^ | .559 | .681 | .568^***^ | .487 | .635 | .627^***^ | .561 | .683 | .441^***^ | .339 | .528 |

*Note.* *n* = 583; ^***^ = *p* < .001, ^**^ = *p* < .01. ^*^ = *p* < .05. ^†^ = *p* < .10; GM fitting = grand-mean fitting, Ind fitting = individual fitting, k-means = k-means clustering, AAHC = atomize and agglomerate hierarchical clustering; ICC = Intraclass correlation coefficient (model = two-way, type = agreement, alpha = .05), LB = lower bound of 95% confidence interval, UB = upper bound of 95% confidence interval. Microstate characteristics: Dur. X = mean duration of microstate types, Occ. X = mean occurrences of microstate types, Cov. X = percentage coverages of microstate types, Exp. Var. = total explained variance by all microstate types, Mean Dur. = mean duration of all microstate types per second, Mean Occ. = mean occurrences of all microstate types per second, Mean GFP = mean global field power in standard deviations, X to X = transitions between microstate types. Shown is the methodological consistency of EEG microstate characteristics across fitting procedures for pre- and post-measures of day one (ICCs of microstate characteristics obtained from GM fitting and Ind fitting). Red (.00 < *ICC* < .50) = poor reliability; yellow (.50 < *ICC* < .75) = moderate reliability, light green (.75 < *ICC* < .90) = good reliability, dark green (*ICC* > .90) = excellent reliability.

**Table S19**

*Methodological consistency of microstate characteristics across fitting procedures (day two)*

|  | **ICCs of day two GM fitting and day two Ind fitting** | | | | | | | | | | | |
| --- | --- | --- | --- | --- | --- | --- | --- | --- | --- | --- | --- | --- |
|  | **Pre-measures** | | | | | | **Post-measures** | | | | | |
| **Parameter** | **k-means** | | | **AAHC** | | | **k-means** | | | **AAHC** | | |
|  | ICC | LB | UB | ICC | LB | UB | ICC | LB | UB | ICC | LB | UB |
| Dur. A | .757^***^ | .712 | .796 | .766^***^ | .723 | .803 | .837^***^ | .804 | .865 | .786^***^ | .747 | .819 |
| Dur. B | .805^***^ | .765 | .838 | .794^***^ | .756 | .826 | .818^***^ | .785 | .847 | .835^***^ | .804 | .860 |
| Dur. C | .791^***^ | .712 | .843 | .782^***^ | .742 | .816 | .827^***^ | .787 | .859 | .790^***^ | .751 | .822 |
| Dur. C’ | .780^***^ | .739 | .815 | .717^***^ | .665 | .761 | .772^***^ | .730 | .808 | .752^***^ | .706 | .791 |
| Dur. D | .722^***^ | .672 | .766 | .756^***^ | .711 | .794 | .774^***^ | .733 | .809 | .757^***^ | .712 | .795 |
| Occ. A | .688^***^ | .630 | .736 | .660^***^ | .598 | .713 | .834^***^ | .804 | .860 | .704^***^ | .650 | .750 |
| Occ. B | .655^***^ | .592 | .708 | .659^***^ | .596 | .712 | .646^***^ | .581 | .701 | .711^***^ | .658 | .756 |
| Occ. C | .567^***^ | .488 | .634 | -.108 | -.365 | .098 | .690^***^ | .633 | .738 | .081 | -.081 | .219 |
| Occ. C’ | .574^***^ | .496 | .640 | .412^***^ | .304 | .504 | .587^***^ | .511 | .651 | .372^***^ | .257 | .470 |
| Occ. D | .697^***^ | .641 | .744 | .637^***^ | .570 | .693 | .715^***^ | .662 | .759 | .657^***^ | .591 | .712 |
| Cov. A | .332^***^ | .210 | .436 | .083 | -.085 | .226 | .642^***^ | .577 | .698 | .138^*^ | -.020 | .272 |
| Cov. B | .364^***^ | .247 | .462 | -.007 | -.190 | .149 | .278^***^ | .145 | .389 | .123^†^ | -.037 | .258 |
| Cov. C | .439^***^ | .330 | .529 | -.051 | -.225 | .101 | .500^***^ | .405 | .579 | .028 | -.126 | .163 |
| Cov. C’ | .424^***^ | .319 | .514 | .034 | -.143 | .183 | .403^***^ | .293 | .496 | .068 | -.103 | .212 |
| Cov. D | .376^***^ | .261 | .473 | .115^†^ | -.045 | .251 | .402^***^ | .292 | .495 | .098 | -.063 | .235 |
| Exp. Var. | .884^*^ | -.101 | .967 | .862^*^ | -.069 | .957 | .872^*^ | -.136 | .965 | .845^*^ | -.135 | .953 |
| Mean Dur. | .967^***^ | .957 | .974 | .964^***^ | .914 | .981 | .976^***^ | .971 | .980 | .965^***^ | .932 | .979 |
| Mean Occ. | .986^***^ | .982 | .989 | .983^***^ | .939 | .992 | .990^***^ | .988 | .992 | .983^***^ | .953 | .992 |
| Mean GFP | .999^***^ | .998 | .999 | .999^***^ | .998 | .999 | .999^***^ | .999 | .999 | .999^***^ | .998 | .999 |
| A to B | .545^***^ | .461 | .616 | .515^***^ | .335 | .636 | .558^***^ | .477 | .626 | .519^***^ | .397 | .612 |
| A to C | .381^***^ | .267 | .477 | .320^**^ | .129 | .461 | .406^***^ | .297 | .498 | .314^***^ | .178 | .426 |
| A to C’ | .390^***^ | .278 | .484 | .382^***^ | .268 | .478 | .577^***^ | .500 | .643 | .281^***^ | .149 | .393 |
| A to D | .454^***^ | .354 | .539 | .337^***^ | .216 | .440 | .469^***^ | .372 | .552 | .318^***^ | .194 | .424 |
| B to A | .547^***^ | .455 | .622 | .512^***^ | .375 | .613 | .581^***^ | .502 | .648 | .508^***^ | .411 | .589 |
| B to C | .264^***^ | .128 | .378 | .266^***^ | .131 | .380 | .329^***^ | .206 | .433 | .381^***^ | .255 | .484 |
| B to C’ | .363^***^ | .246 | .462 | .388^***^ | .276 | .483 | .420^***^ | .314 | .510 | .375^***^ | .260 | .472 |
| B to D | .266^***^ | .132 | .380 | .251^***^ | .114 | .368 | .429^***^ | .324 | .517 | .389^***^ | .277 | .483 |
| C to A | .341^***^ | .220 | .443 | .246^***^ | .109 | .362 | .532^***^ | .446 | .604 | .285^***^ | .154 | .396 |
| C to B | .382^***^ | .268 | .477 | .244^***^ | .106 | .361 | .327^***^ | .203 | .431 | .154^*^ | .000 | .284 |
| C to C’ | .455^***^ | .355 | .540 | .382^***^ | .269 | .478 | .487^***^ | .393 | .567 | .201^**^ | .056 | .325 |
| C to D | .357^***^ | .240 | .457 | .338^***^ | .216 | .441 | .454^***^ | .354 | .539 | .424^***^ | .318 | .514 |
| C’ to A | .414^***^ | .307 | .505 | .373^***^ | .257 | .470 | .483^***^ | .388 | .563 | .425^***^ | .319 | .514 |
| C’ to B | .263^***^ | .129 | .377 | .292^***^ | .163 | .401 | .416^***^ | .309 | .506 | .319^***^ | .195 | .424 |
| C’ to C | .522^***^ | .434 | .596 | .490^***^ | .396 | .569 | .501^***^ | .409 | .578 | .382^***^ | .269 | .477 |
| C’ to D | .587^***^ | .511 | .651 | .575^***^ | .479 | .652 | .563^***^ | .483 | .631 | .580^***^ | .494 | .650 |
| D to A | .342^***^ | .222 | .444 | .375^***^ | .259 | .472 | .427^***^ | .322 | .516 | .410^***^ | .301 | .501 |
| D to B | .379^***^ | .265 | .475 | .411^***^ | .302 | .502 | .472^***^ | .375 | .554 | .382^***^ | .269 | .478 |
| D to C | .499^***^ | .407 | .576 | .249^***^ | .112 | .365 | .417^***^ | .310 | .507 | .438^***^ | .333 | .526 |
| D to C’ | .576^***^ | .499 | .642 | .541^***^ | .449 | .616 | .596^***^ | .522 | .659 | .536^***^ | .450 | .608 |

*Note.* *n* = 542; ^***^ = *p* < .001, ^**^ = *p* < .01. ^*^ = *p* < .05. ^†^ = *p* < .10; GM fitting = grand-mean fitting, Ind fitting = individual fitting, k-means = k-means clustering, AAHC = atomize and agglomerate hierarchical clustering; ICC = Intraclass correlation coefficient (model = two-way, type = agreement, alpha = .05), LB = lower bound of 95% confidence interval, UB = upper bound of 95% confidence interval. Microstate characteristics: Dur. X = mean duration of microstate types, Occ. X = mean occurrences of microstate types, Cov. X = percentage coverages of microstate types, Exp. Var. = total explained variance by all microstate types, Mean Dur. = mean duration of all microstate types per second, Mean Occ. = mean occurrences of all microstate types per second, Mean GFP = mean global field power in standard deviations, X to X = transitions between microstate types. Shown is the methodological consistency of EEG microstate characteristics across fitting procedures for pre- and post-measures of day two (ICCs of microstate characteristics obtained from GM fitting and Ind fitting). Red (.00 < *ICC* < .50) = poor reliability; yellow (.50 < *ICC* < .75) = moderate reliability, light green (.75 < *ICC* < .90) = good reliability, dark green (*ICC* > .90) = excellent reliability.

A

B

**Figure S1.** Electrode configurations of day one and day two

A: Electrode configuration of the 64-electrode system (Brain Products, Gilching, Germany) used for day one EEG recordings. B: Electrode configuration of the 30-electrode system (BioSemi B. V., Amsterdam, Netherlands) used for day two EEG recordings.
